# Impact of Threading State on the Mechanics of γ‐Cyclodextrin–Pyrene Gels

**DOI:** 10.1002/anie.1958506

**Published:** 2026-08-22

**Authors:** Jongwon Oh, Heyi Liang, Natsumi Nitta, Jongmin Kim, Yuan Tian, Guancen Liu, Jerald E. Hertzog, Juan J. de Pablo, Stuart J. Rowan

**Affiliations:** ^1^ Pritzker School of Molecular Engineering University of Chicago Chicago Illinois USA; ^2^ Department of Chemistry University of Chicago Chicago Illinois USA; ^3^ Department of Chemical and Biomolecular Engineering, Tandon School of Engineering New York University Brooklyn New York USA; ^4^ Department of Computer Science, Courant Institute of Mathematics, Computing, and Data Sciences New York University New York New York USA; ^5^ Department of Physics New York University New York New York USA; ^6^ Chemical Science and Engineering Division and Center for Molecular Engineering Argonne National Laboratory Chicago Illinois USA

**Keywords:** [4]rotaxane, host–guest, mechanically interlocked polymers, slide‐ring networks, stretchable materials

## Abstract

Conventional covalent and supramolecular crosslinks impose inherent trade‐offs between mechanical robustness and environmental adaptability in polymer networks. Mechanically interlocked junctions offer an alternative route by enabling topological motion without compromising structural integrity. Here, a modular approach is reported to construct topology‐engineered gels by copolymerizing 2‐hydroxyethyl acrylate (HEA) with doubly threaded (dt‐) pseudo[4]rotaxane crosslinkers, formed via the complexation of γ‐cyclodextrin (γ‐CD) and acrylate‐end‐capped pyrene derivatives. By tuning the HEA/water ratio in pre‐gel formulations, the supramolecular assembly can be shifted from dt‐ to singly threaded (st‐) entities, enabling access to gels with the same chemical composition but different architectures—dt‐slide ring network versus st‐polyrotaxane covalent network. The dt‐network exhibits solvent‐dependent fluorescence, swelling, and viscoelasticity that is not observed in either the st‐network or a covalent control gel. Mechanical testing of the bulk networks reveals that the dt‐network exhibits high extensibility (>1050% strain at break), strain rate‐dependent stiffening and rupture, and a favorable combination of high toughness and comparatively low hysteresis under cyclic deformation that is not observed in the networks lacking the dt‐architecture. Overall, these results establish a structure–property relationship dictated by crosslinking topology, demonstrating how dt‐interlocked motifs can encode both mechanical performance and environmental responsiveness in soft materials.

## Graphical abstract

Tuning the threading state of discrete γ‐cyclodextrin (γ‐CD)–pyrene pseudorotaxane crosslinkers controls gel topology and ring mobility. The resulting doubly threaded slide‐ring networks exhibit solvent‐switchable dissipative dynamics, high extensibility, rate‐dependent mechanics, and low hysteresis, revealing how crosslink topology governs macroscopic mechanical response through the junction mobility.

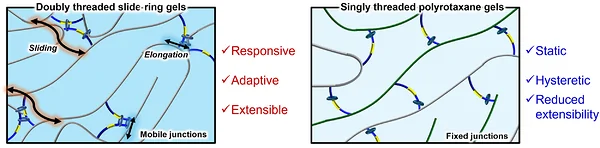

## 1 Introduction

Designing soft materials that integrate mechanical robustness with adaptive functionality remains one of the central challenges in materials science. Crosslinked polymer networks constitute a foundational architecture for a diverse range of such materials, offering structural robustness (e.g., shape retention) [1, 2, 3]. The nature of the crosslinks that couple the polymer chains serves as one of the critical factors that affect the macroscopic properties of polymer networks, including viscoelasticity, swelling behavior, extensibility, and mechanical toughness [3, 4, 5, 6, 7, 8, 9]. Among the available crosslinking strategies, covalent crosslinks, constructed through irreversible chemical bonds, are generally employed on account of their ability to impart high mechanical strength and chemical stability toward organic solvents [10, 11]. However, the static and permanent nature of these linkages intrinsically restricts network adaptability, often leading to brittle fracture and poor processability. To overcome these limitations, dynamic crosslinking strategies, including both dynamic covalent bonds and supramolecular interactions, have emerged as promising alternatives [12, 13, 14, 15, 16]. For example, supramolecular crosslinks, formed through non‐covalent interactions such as hydrogen bonding [17, 18, 19, 20], metal–ligand coordination [21, 22, 23], host–guest complexation [24, 25, 26, 27], and π–π stacking [28, 29, 30], can be rearranged through reversible bond dissociation and reformation under relatively mild conditions (e.g., moderate temperature changes, pH variations, or solvent exposure) on account of their relatively low bonding energies, facilitating stress relaxation [31, 32, 33], reprocessability [34, 35, 36], and in some cases, self‐healing capabilities [36, 37, 38, 39]. However, this intrinsic reversible nature results in the reduction of structural robustness, leading to challenges in balancing adaptability with long‐term durability.

In one approach to overcome these limitations, significant efforts have recently been devoted to exploring topological crosslinking approaches, particularly through the use of mechanically interlocked moieties [40, 41, 42, 43] such as catenanes [44, 45, 46] and rotaxanes [47, 48, 49, 50], which preserve network structural integrity while allowing mobility without bond breaking. Such interlocked molecules, composed of two or more molecular components bound together through mechanical bonds, can exhibit relative component mobility through sliding, elongation, and rotation without bond dissociation. When employed as crosslinks in polymer networks, these mechanically interlocked crosslinks (MICs) can facilitate uniform stress distribution and energy dissipation, resulting in materials with mechanical resilience, improved extensibility, and enhanced toughness [51, 52, 53, 54, 55, 56].

Slide‐ring gel networks (SRGs), which can be fabricated by covalent bonding of singly threaded (st‐) rings on two different main‐chain polyrotaxane chains, exemplify the concept of MIC‐based networks. For example, covalently bonded rings can function as mobile figure‐of‐eight crosslinks capable of sliding along the polymer chains [57, 58, 59, 60, 61, 62]. Other types of related rotaxane‐ or polyrotaxane‐crosslinked networks [63, 64, 65, 66, 67] have also been reported. Generally, these materials exhibit highly extensible and elastic behavior on account of crosslink mobility, including what has been termed the pulley effect, where sliding of rings along the backbone allows for uniform stress distribution across the network. Recent advances have extended this concept through the design of the doubly threaded (dt‐) architectures, where a single large macrocycle interlocks two separate polymer strands, forming dt‐mechanically interlocked polymers (dt‐MIPs) [68, 69, 70, 71, 72, 73, 74, 75]. Among these, dt‐MIPs constructed via metal–templating strategies [72, 73, 74, 75] have demonstrated that ring mobility can be modulated through the addition/removal of metal cations (e.g., Zn^2+^ and Cu^2+^), leading to reversible alteration of macroscopic properties such as viscoelasticity, stress relaxation, and swelling behavior. Despite their potential, dt‐MIPs remain relatively underexplored due to synthetic challenges, particularly the need for specialized large macrocyclic ligands that limit scalability. To address these challenges, Ke and coworkers have recently reported a synthetically accessible strategy for constructing dt‐MIPs using the crystalline‐domain‐reinforced pro‐slide‐ring crosslinkers [71]. These supramolecular crosslinkers are formed via kinetically driven crystallization of preassembled dt‐γ‐cyclodextrin (γ‐CD) complexes with azobenzene‐terminated telechelic poly(ethylene glycol) (PEG) axles, leading to rapid gelation and 3D‐printable hydrogel formation. While this polymeric crosslinker‐based approach improves scalability and mechanical robustness, it leaves unresolved questions regarding how discrete threading topology and corresponding ring mobility at the molecular level influence macroscopic material properties.

The goal of this work is to establish a molecularly defined platform based on a discrete γ‐CD–pyrene pseudorotaxane crosslinker that enables systematic control over threading topology and ring mobility in the gel networks (Figure 1). Pyrene, a polycyclic aromatic, exhibits strong affinity toward CDs in aqueous environments on account of favorable hydrophobic interactions, forming a stable inclusion complex through the penetration of pyrene moieties into the hydrophobic CD cavities [76, 77, 78, 79, 80, 81]. Specifically, the relatively large internal cavity of γ‐CD allows for the simultaneous accommodation of two pyrene moieties, enabling the formation of dt‐host–guest complexes [79, 80, 81]. As this host–guest topology is sensitive to local solvation and hydrophobicity, this study aims to regulate the supramolecular species in the pre‐gel solution and translate it into polymer network architectures. This strategy enables the synthesis of a series of topological networks and provides a framework to examine how molecular‐level control over crosslink architecture translates into macroscopic material properties.

**FIGURE 1:**
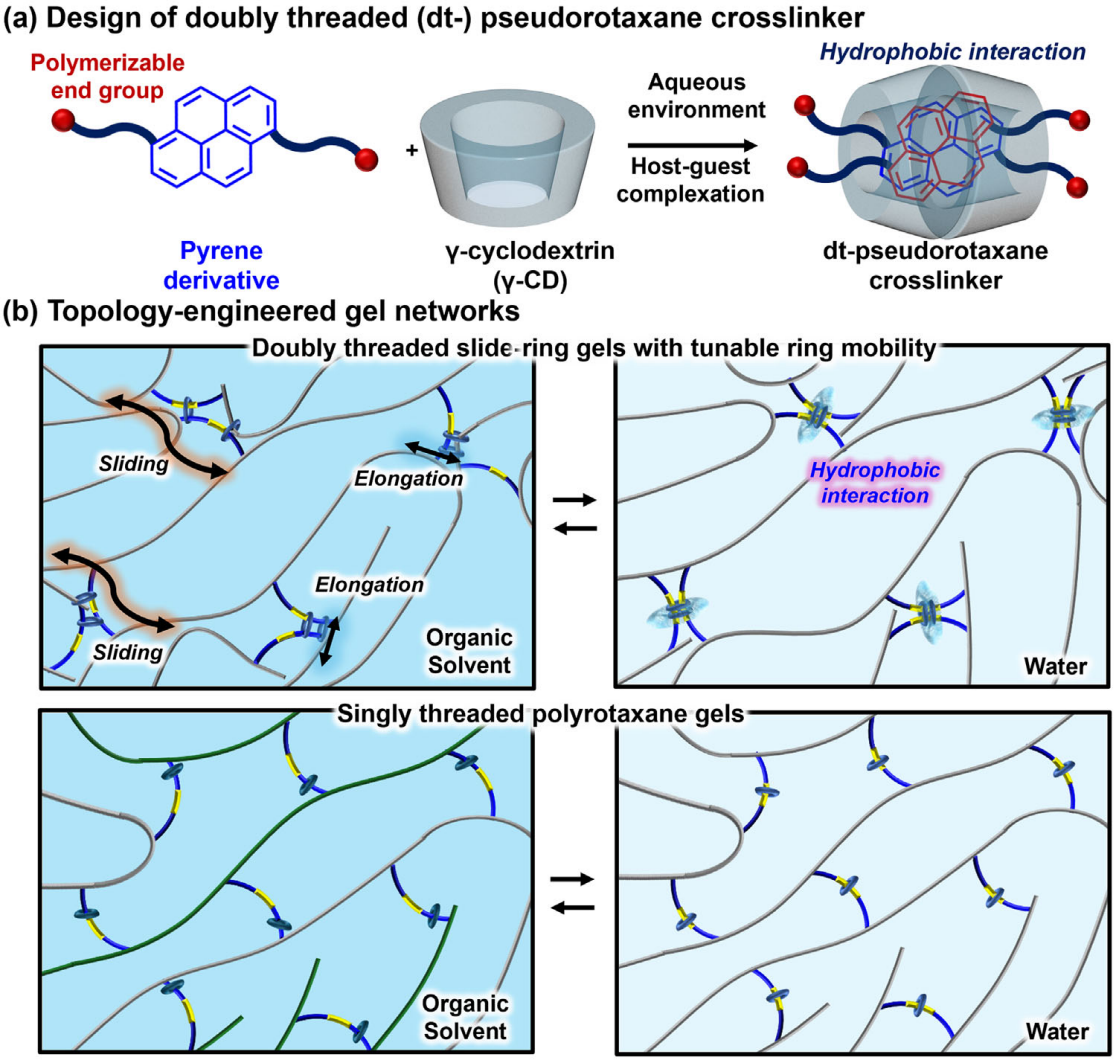
Modular construction of topology‐engineered gel networks based on discrete doubly threaded (dt‐) pseudorotaxane crosslinkers. (a) Schematic illustration of host–guest complexation between a polymerizable pyrene thread and γ‐CD in aqueous media, forming a discrete dt‐pseudorotaxane crosslinker driven by hydrophobic interactions. (b) Conceptual representation of gel networks with distinct crosslinking topologies programmed by the nature of supramolecular crosslinking species. dt‐slide‐ring gels can be accessed through polymerization of a dt‐pseudo[4]rotaxane while polymerization of pseudo[2]rotaxane yields singly threaded (st‐) polyrotaxane gels.

## 2 Results and Discussion

To rationally design a pyrene derivative capable of forming doubly threaded (dt‐) γ‐CD inclusion complexes while remaining compatible with aqueous polymerization, three key molecular features were targeted: (i) a hydrophobic aromatic core to drive host–guest complexation, (ii) hydrophilic substituents to enhance aqueous solubility, and (iii) terminal polymerizable groups to enable network formation. Based on previous reports describing dt‐inclusion complex formation of pyrene dimers with γ‐CD [79, 80, 81], the pyrene core was selected as the central hydrophobic aromatic unit. As a first step, the complexation behavior of a phenyl‐extended pyrene core with γ‐CD was examined computationally using a minimal model, **1^1^OH** (where n in **1^n^** denotes the number of ethylene oxide (EO) units on each arm), consisting of a pyrene core bearing two *p*‐phenoxyethanol moieties on the 1 and 6 positions (Figure 2a). Accordingly, the binding process was modeled using a dimeric species of **1^1^OH** (Figure 2a). The corresponding free energy surface was calculated as a function of the axial separation between the **1^1^OH** dimer and the γ‐CD narrow rim (See Supporting Information for details). The resulting free energy profile reveals two distinct minima, in which the phenoxy moieties at one end of the **1^1^OH** dimer are included within the γ‐CD cavity, while the bulky pyrene cores remain only partially encapsulated near either the wide rim (global minimum) or the narrow rim (local minimum) of the γ‐CD (Figure 2b). Configurations between the two minima, corresponding to the accommodation of the pyrene moiety of the **1^1^OH** dimer by γ‐CD, are energetically disfavored due to steric crowding. Notably, in both low‐energy configurations, the central region of the **1^1^OH** dimer remains exposed and sterically accessible, suggesting the possibility of accommodating an additional γ‐CD molecule. To probe the binding of a second γ‐CD, which would yield a 2:2 complex, the corresponding free energy surface was obtained by sampling two collective variables (CVs): the axial separation between the two γ‐CD rings (CV1) and the axial displacement of the pyrene dimer relative to the first‐bound γ‐CD (CV2) (see Supporting Information for details). The resulting free energy landscape reveals a broad region of low free energy at short γ‐CD separations, indicating that configurations in which the two γ‐CD rings are closely spaced around the pyrene dimer are energetically favored (Figure 2c). These results highlight the critical role of the phenyl substituents in mediating favorable host–guest interactions and stabilizing the 2:2 inclusion complex geometry, underscoring their importance in the molecular design of the aromatic core.

**FIGURE 2:**
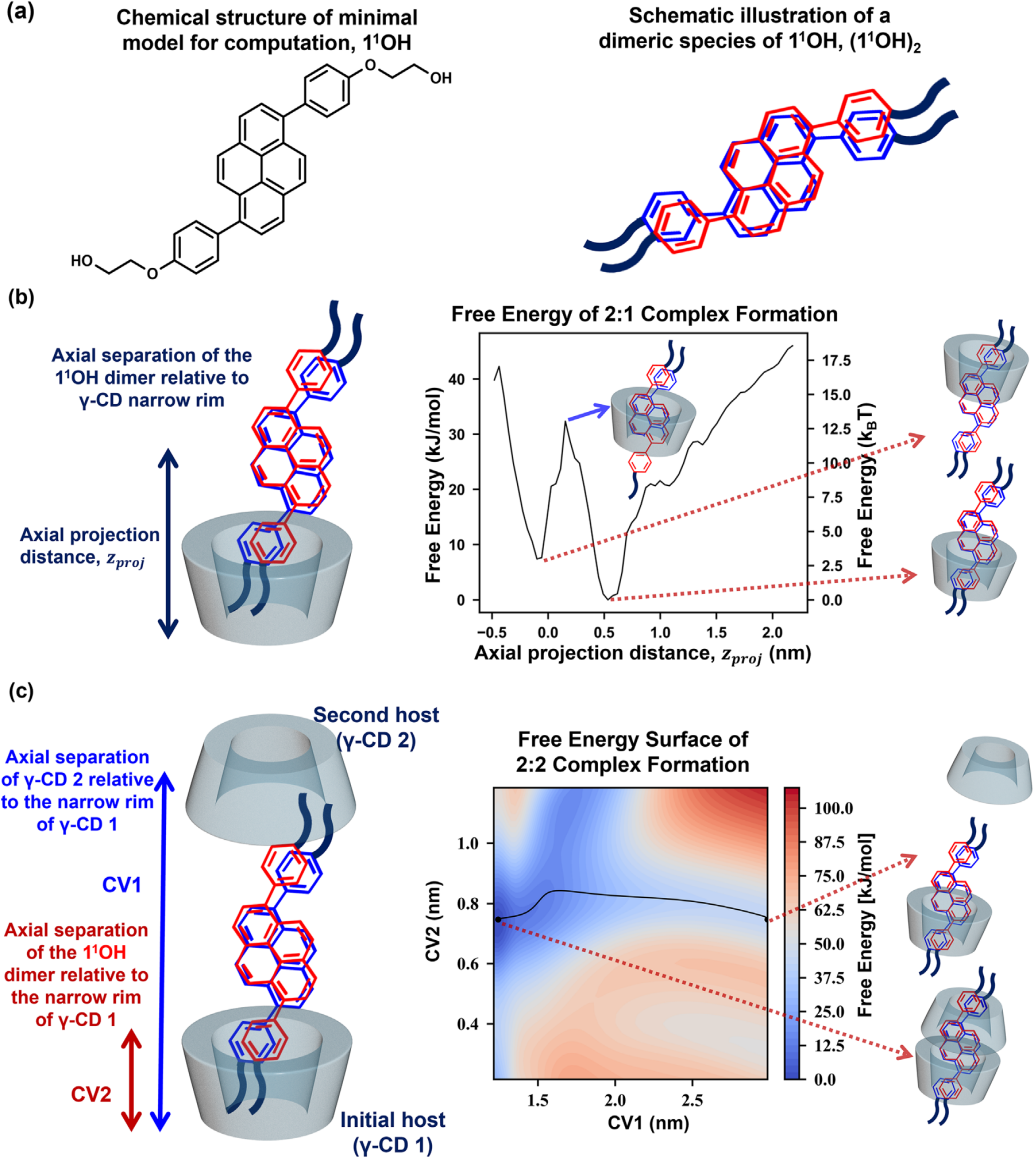
Computational free‐energy analysis of γ‐CD–pyrene complex formation using **1^1^OH** as a minimal thread model. (a) Chemical structure of pyrene thread **1^1^OH** (left) and schematic illustration of its dimeric species (right). (b) One‐dimensional free energy of γ‐CD and the **1^1^OH** dimer as a function of the axial projection distance, *z_proj_*, defined as the projection of the vector between the center of mass of the **1^1^OH** dimer and the center of γ‐CD narrow rim onto the central axis of γ‐CD. (c) Two‐dimensional free energy surface of 2:2 complex formation sampled using collective variables: CV1, the axial separation between two γ‐CD rings, and CV2, the axial separation of the **1^1^OH** dimer relative to the narrow rim of γ‐CD 1. Representative configurations along the complexation pathway are shown. See Supporting Information for detailed definition of the collective variables.

Guided by these computational insights, an acrylate end‐capped pyrene derivative (**1^9^Acr**) was synthesized by incorporating nine ethylene glycol (EG) units with terminal acrylate groups on both sides of the phenyl pyrene core. Specifically, the phenyl extension was used to enhance γ‐CD association through an expanded aromatic binding surface, while the EG spacers and the terminal acrylate groups impart the necessary aqueous compatibility and polymerizability, respectively. The diacrylate **1^9^Acr** was prepared via a two‐step process involving etherification of 1,6‐bis(hydroxyphenyl)pyrene with mono‐tosylated nonaethylene glycol, followed by esterification with acryloyl chloride (Figures S1–S3; see Supporting Information for full synthetic details).

The complexation behavior of **1^9^Acr** with γ‐CD was initially examined via fluorescence spectroscopy in aqueous media (Figure 3a,b, see Supporting Information for details). γ‐CD (2.0 mM) was titrated into an aqueous solution of **1^9^Acr** (0.2 mM) while maintaining a constant concentration of **1^9^Acr**. Upon titration, the emission maximum gradually blue‐shifted from ca. 480 nm to ca. 450 nm along with an intensity enhancement, reaching saturation at [γ‐CD]/[**1^9^Acr**] = 8. To interpret this spectral evolution, the fluorescence of **1^9^Acr** was compared across different solvent environments (Figure 3c). In dimethyl sulfoxide (DMSO, a good solvent), **1^9^Acr** exhibits a monomeric emission at ca. 420 nm, whereas in water it shows a broad aggregate band at ca. 480 nm. Notably, the saturated emission at ca. 450 nm is distinct from both the monomeric (ca. 420 nm) and aggregated (ca. 480 nm) states, indicating the emergence of a new emissive environment driven by γ‐CD‐mediated disruption of pyrene aggregates and subsequent host–guest complexation.

**FIGURE 3:**
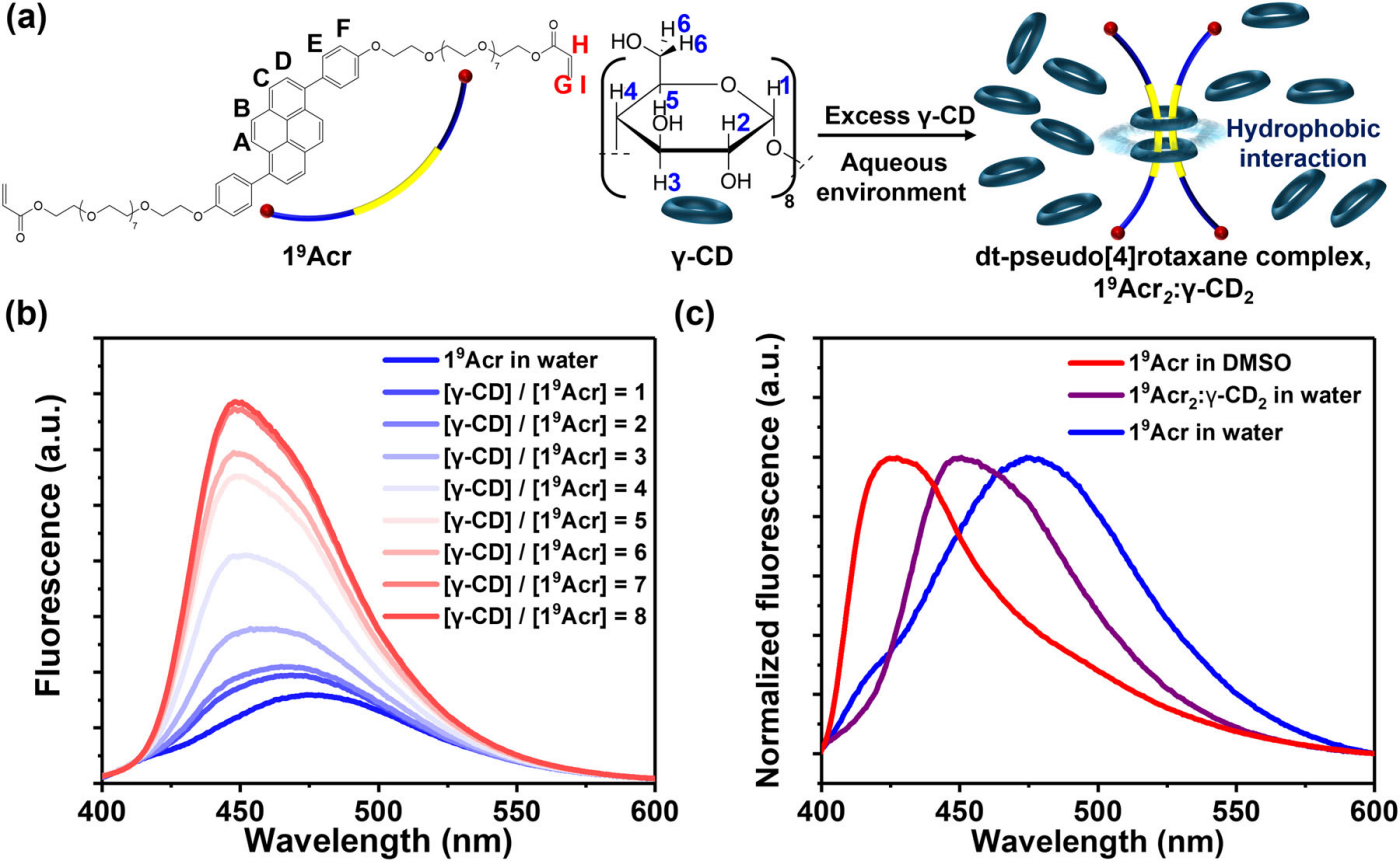
Fluorescence characterization of dt‐pseudorotaxane inclusion complexes formed between **1^9^Acr** and γ‐CD. (a) Chemical structure of **1^9^Acr** and γ‐CD, and schematic illustration of the proposed dt‐inclusion complex (**1^9^Acr_2_:γ‐CD_2_**). (b) Fluorescence spectra of **1^9^Acr** upon titration with γ‐CD (2 mM) at a constant concentration of **1^9^Acr** (0.2 mM). (c) Normalized fluorescence spectra of **1^9^Acr** in water and DMSO, and of the **1^9^Acr_2_:γ‐CD_2_** inclusion complex in water. All spectra were recorded at an excitation wavelength of 375 nm.

To gain further insight into the complexation process, proton nuclear magnetic resonance (^1^H NMR) analyses were carried out by titrating γ‐CD into an aqueous solution containing **1^9^Acr** (2.0 mM) while maintaining the concentration of **1^9^Acr** (Figure 4a, see Supporting Information for details). For **1^9^Acr** in D_2_O, signals at 6.20, 5.95, and 5.68 ppm are observed corresponding to the unbound acrylate protons of G, H, and I (shown in red), while no aromatic peaks are observed, consistent with the formation of π‐π pyrene‐based aggregates of **1^9^Acr**. Upon addition of the γ‐CD, new aromatic signals (A‐F; shown in black) and bound acrylate signals at 6.50, 6.28, and 6.05 ppm (G, H, and I; shown in black) appeared, indicative of formation of an inclusion complex (Figures 4a, S4, and S5). In line with the fluorescence results, at a [γ‐CD]/[**1^9^Acr**] = 8, signals for the unbound thread at 6.20, 5.95, and 5.68 ppm completely disappeared, suggesting that nearly all **1^9^Acr** molecules are bound within the γ‐CD. In addition, a new γ‐CD peak is also observed at 4.98 ppm and is assigned to the bound γ‐CD H_1_ peak. Integration of the bound H_C_ peak in **1^9^Acr** (8.25 ppm) and bound H_1_ peak in γ‐CD (4.98 ppm) confirmed an equimolar complexation stoichiometry, in agreement with the formation of n:n complexes (Figure S6). It is important to note that a substantial fraction of γ‐CD remains uncomplexed under these conditions. The ratio of bound to unbound γ‐CD, estimated from the average integrals of the H_1_ resonances across three independent samples was ca. 1:7 (Figure S7). This requirement for excess γ‐CD likely reflects the need to disrupt pre‐associated aggregates of **1^9^Acr** in water and shift the equilibrium toward inclusion complex formation. Under these conditions ([γ‐CD]/[**1^9^Acr**] = 8, [**1^9^Acr**] = 2 mM), diffusion‐ordered spectroscopy (DOSY) NMR measurements demonstrated that the bound signals of **1^9^Acr** (H_A‐I_) and γ‐CD (H_1_) exhibited nearly identical diffusion coefficients (ca. 1.26 × 10^−10^ m^2^/s), which is lower than that of H_1_ in unbound γ‐CD (ca. 2.26 × 10^−10^ m^2^/s) (Figure S8). This change in diffusion behavior provides direct evidence that the observed bound resonances arise from a single, slower‐diffusing inclusion complex, thereby differentiating them from free γ‐CD species.

**FIGURE 4:**
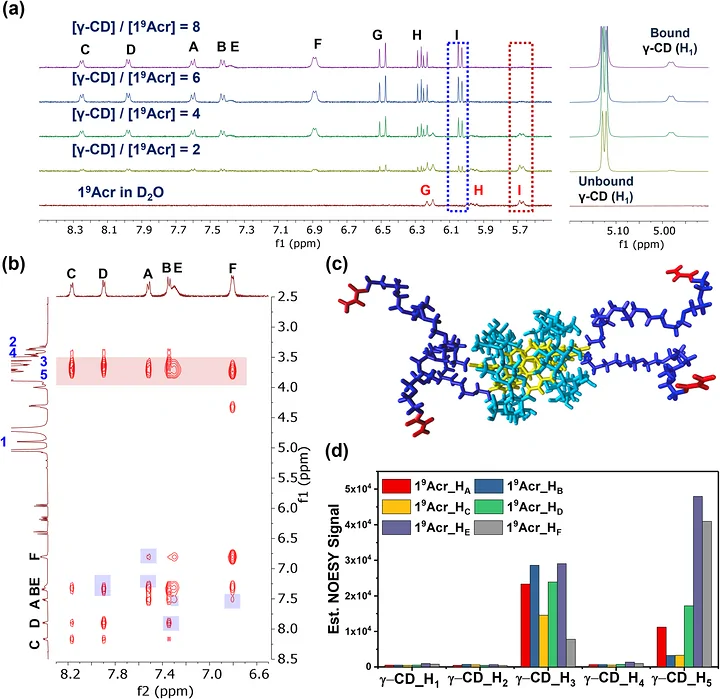
NMR and structural analysis of dt‐pseudorotaxane inclusion complexes formed between **1^9^Acr** and γ‐CD. (a) ^1^H NMR spectra (500 MHz, 25 °C, D_2_O) of **1^9^Acr** (2.0 mM) at varying [γ‐CD]/[**1^9^Acr**] molar ratios (0–8, bottom to top), showing disappearance of unbound acrylate signals (G­I, red) and emergence of bound resonances from both guest **1^9^Acr** (A‐F, G‐I, black) and host γ‐CD (H_1_). (b) 2D NOESY spectrum of **1^9^Acr** with γ‐CD ([**1^9^Acr**] = 2 mM, [γ‐CD]/[**1^9^Acr**] = 8), showing γ‐CD cavity (H_3_/H_5_)–aromatic H_A_–H_F_ correlation (red) and inter‐aromatic H_A_–H_F_ cross‐peaks indicative of dimer formation (light blue). (c) Atomistic molecular model of the **1^9^Acr_2_:γ‐CD_2_** complex illustrating the dt‐configuration and positioning of aromatic units (yellow) and γ‐CD units (cyan) in explicit water (not shown). The 9EG segments are shown in blue and the acrylate chain ends are shown in red. (d) Estimated NOESY signal between γ‐CD protons (H_1_–H_5_) and aromatic protons of **1^9^Acr** (H_A_–H_F_). The proton assignments for **1^9^Acr** (A–I) and γ‐CD (H_1_–H_6_) are shown in Figure 3a.

The resulting complex was further explored by nuclear Overhauser effect spectroscopy (NOESY) (Figure 4b). Distinct cross‐peaks were observed between the aromatic protons of **1^9^Acr** and the interior H_3_/H_5_ protons of γ‐CD (highlighted in red), confirming the formation of γ‐CD inclusion complexes. Moreover, inter‐aromatic cross‐peaks between thread protons from **1^9^Acr** (H_A_–H_E_, H_A_–H_F_, H_B_–H_D_ highlighted in blue) are observed. The peaks are absent in the unbound state (Figure S9), consistent with two threads in close spatial proximity within the complex and suggesting the formation of a 2:2 complex. Additional atomistic simulations with explicit water were conducted to further investigate these 2:2 complexes (Figure 4c). The NOESY signals from simulations were estimated to be proportional to the ensemble average 〈*r* ^−6^〉, where *r* is the inter‐proton distance. Computational NOESY analysis reveals strong interactions between H_3_/H_5_ of γ‐CD and the aromatic protons of **1^9^Acr** (with signals for H_A_, H_B_, H_D_, H_E_ with H_3_ and intense signals for H_E_, H_F_ with H_5_), which is broadly consistent with experimental observations (Figure 4d). In addition, the simulations predict enhanced inter‐aromatic H cross‐peaks between protons located on different regions of the phenyl pyrene core, for example between proton A on the pyrene ring and proton E on the phenyl ring. These correlations suggest that the two phenyl pyrene cores adopt a closely stacked arrangement within the 2:2 complex (Figure S10), which may also enhance intramolecular A–E proximity by restricting rotation between the pyrene and phenyl rings. Although the precise pattern of aromatic–aromatic correlations differ somewhat between simulation and experiment (with A–E prominent in both, B–D and A–F dominant in the experimental spectra, and D–E in the simulations), both data sets consistently support close spatial proximity between the two phenyl pyrene cores (Figures 4b and S10). These NOESY results, taken together with the complex stoichiometry, DOSY data and fluorescence data, support the conclusion that two thread units are simultaneously encapsulated within γ‐CD cavities, consistent with a dt‐pseudo[4]rotaxane complex, **1^9^Acr_2_**:**γ‐CD_2_** predicted by the computational modeling above.

Having demonstrated the formation of the dt‐inclusion complex, the next step was to prepare pre‐gel solutions by mixing an aqueous solution of the **1^9^Acr_2_**:**γ‐CD_2_** with the 2‐hydroxyethyl acrylate (HEA) comonomer (Figure 5a). Given that the molecular assembly of the **1^9^Acr_2_**:**γ‐CD_2_** is driven primarily by hydrophobic interactions, it was expected that the addition of significant amounts of the HEA comonomer would influence the host–guest complexation (Figure 5b). To investigate the effect of HEA content on the threading assembly, fluorescence spectroscopy was employed to monitor changes in pre‐gel solutions prepared with varying HEA‐to‐water ratios. An aqueous solution containing **1^9^Acr** (1.12 mM) and γ‐CD (8.96 mM) was titrated with increasing amounts of HEA while maintaining constant concentrations of **1^9^Acr** and γ‐CD (see Supporting Information for details). At a low HEA‐to‐water ratio (11 vol%:89 vol%), the emission maximum is centered at ca. 450 nm, consistent with the inclusion complexes largely retaining the dt‐structures **1^9^Acr_2_:γ‐CD_2_** (Figure 5c). Upon increasing HEA content, the emission band at ca. 450 nm gradually decreased and is replaced with a new emission at ca. 420 nm. This spectral evolution is consistent with a progressive dissociation of the **1^9^Acr_2_:γ‐CD_2_** complexes, likely arising from the co‐solvent effect of the HEA, which weakens hydrophobic associations and favors the formation of less‐associated pyrene species, such as singly threaded (st‐) or unthreaded (free) threads. To provide a semi‐quantitative description of the solvent‐dependent spectral evolution, a relative fluorescence index (FI), calculated as *I*_450_/(*I*_420_ + *I*_450_), was extracted from the normalized fluorescence spectra (Figure 5c, inset). The relative FI decreases continuously with increasing HEA content, consistent with the progressive evolution toward less‐associated pyrene species.

**FIGURE 5:**
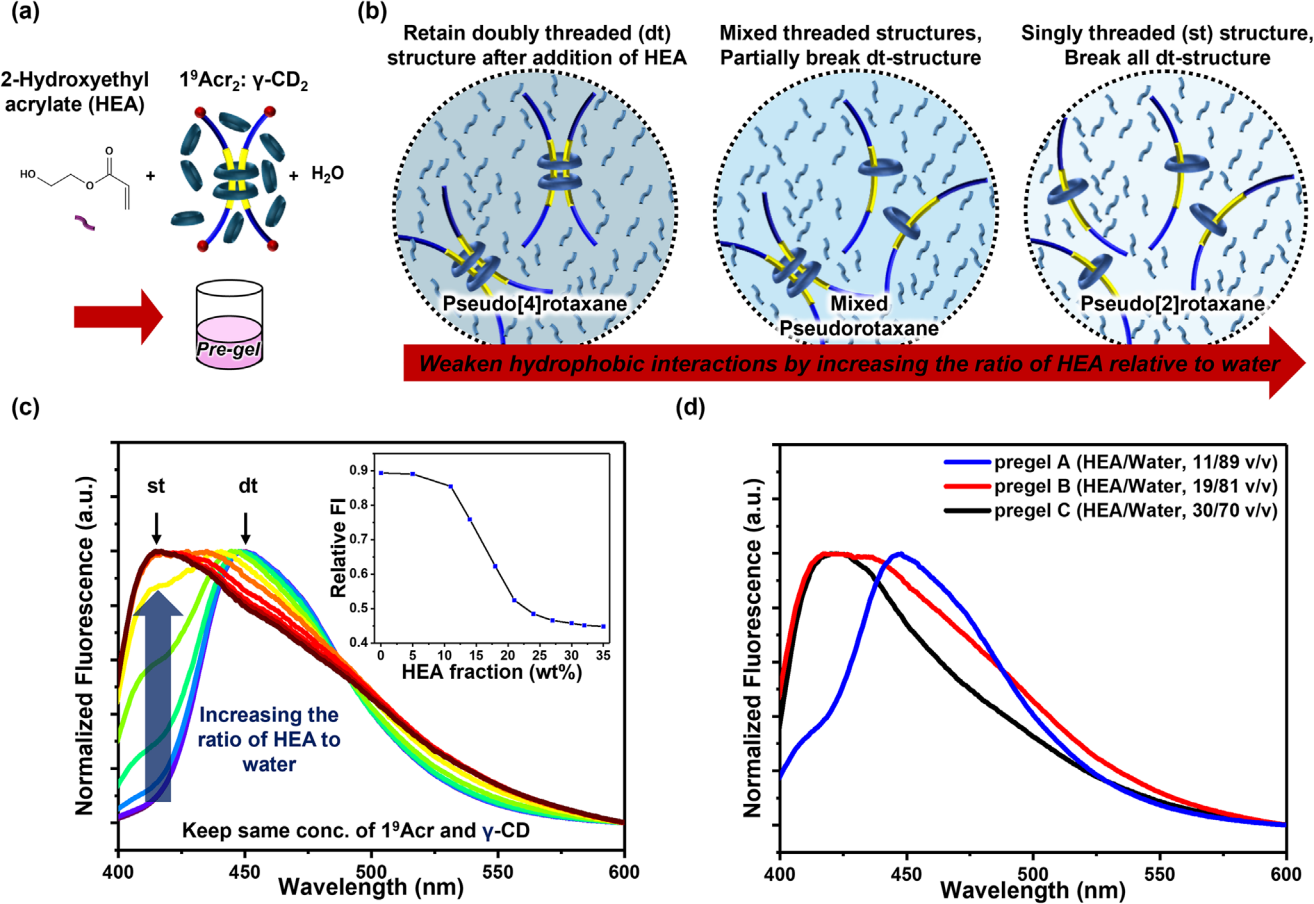
Control of threading topology in pre‐gel solutions by tuning the HEA‐to‐water ratio. (a) Schematic illustration of pre‐gel formulations comprising **1^9^Acr**, γ‐CD, and 2‐hydroxyethyl acrylate (HEA) in water. (b) Conceptual illustrations of threading topology as a function of HEA‐to‐water ratio, showing a transition from dt‐pseudo[4]rotaxane to st‐pseudo[2]rotaxane structures with increasing HEA fraction. (c) Normalized fluorescence spectra of pre‐gels with increasing HEA‐to‐water ratios while maintaining constant concentrations of **1^9^Acr** and γ‐CD. The inset shows the relative fluorescence index (FI), calculated as *I*_450_/(*I*_420_ + *I*_450_) from the normalized emission intensities. (d) Normalized fluorescence spectra of three pregel representative formulations (pre‐gel A‐C, see Table S1) with varying water content. All spectra were recorded at an excitation wavelength of 375 nm.

The dependence of the threaded structure on co‐solvent composition was then examined by NMR spectroscopy. To enable direct spectral observation without interference from HEA resonances, DMSO‐d_6_/D_2_O mixtures were prepared in which HEA is replaced by DMSO‐d_6_. Thus, the samples A–C were prepared with different DMSO‐d_6_:D_2_O ratios using a stock DMSO‐d_6_ solution of **1^9^Acr** and γ‐CD ([γ‐CD]/[**1^9^Acr**] = 8), splitting it into three samples, and adding different amounts of D_2_O. The resulting three samples: A (DMSO‐d_6_/D_2_O, 11/89 v/v), B (19/81 v/v), and C (30/70 v/v) had a constant [γ‐CD]/[**1^9^Acr**] ratio of 8 with [**1^9^Acr**] = 0.50, 0.91, and 1.41 mM, respectively, based on the total DMSO‐d_6_/D_2_O volume. As the DMSO‐d_6_ ratio increased, the emission maximum shifted to shorter wavelength, similar to the trend observed upon increasing the HEA fraction in the HEA/water systems, suggesting that the two co‐solvents induce similar effects on the assembly (Figure S11). The corresponding DMSO‐d_6_/D_2_O solutions also exhibited distinct ^1^H NMR spectra. Two additional intermediate solvent compositions (15/85 and 25/75, v/v) were prepared between Samples A, B, and C to better understand the solvent‐dependent evolution of the threading state (Figure S12). In sample A, the acrylate resonances are sharp and well‐resolved at the chemical shifts related to the bound state (6.5, 6.28, and 6.05 ppm). In sample B, these bound peaks are still present, but additional broader signals appear at different chemical shifts, consistent with a mixture of dt‐ and less‐threaded species. In sample C (DMSO‐d_6_/D_2_O, 30/70 v/v), the spectra are dominated by broadened acrylate signals, with only weak features remaining at the bound chemical shifts. The intermediate compositions exhibited gradual spectral changes between Samples A, B, and C, further supporting the solvent‐dependent evolution of the threading state. This systematic evolution is consistent with a solvent‐induced weakening of the dt‐structure, wherein competitive solvation by DMSO‐d_6_ progressively disrupts the host–guest complexation.

NOESY measurements on sample C revealed weak γ‐CD (H_3_/H_5_)–pyrene NOE correlations but no well‐defined inter‐aromatic NOEs between different pyrene units (Figure S13a). Importantly, in neat DMSO‐d_6_, no γ‐CD–pyrene NOE correlations were detected, consistent with the absence of inclusion complexes in this solvating medium (Figure S14). The persistence of γ‐CD–pyrene NOE cross‐peaks in sample C therefore supports the presence of singly threaded inclusion complexes rather than only decomplexed free pyrene. Temperature‐dependent NOESY of sample C exhibited increasingly pronounced γ‐CD–pyrene cross‐peaks upon cooling, consistent with strengthened host–guest interactions and slower exchange dynamics. Notably, inter‐thread NOE cross‐peaks, characteristic of the formation of a stacked 2:2 geometry, were not observed over the entire temperature range despite excess γ‐CD ([γ‐CD]/[**1^9^Acr**] = 8), supporting a predominantly st‐state under these conditions (Figure S13).

On the basis of this spectroscopic analysis, three HEA‐based pre‐gel formulations were prepared at HEA/water ratios corresponding to the DMSO‐d_6_/D_2_O model systems described above. The ratio of **1^9^Acr** (1 equivalent), γ‐CD (8 equivalents) and HEA (2000 equivalents) is kept constant and the water content was varied to yield the pre‐gels A, B, and C, (Table S1; with HEA/H_2_O of 11/89, 19/81 and 30/70 and [**1^9^Acr**] = 0.50, 0.91, and 1.41 mM, respectively, calculated based on the total volume of HEA and water). Each formulation exhibited a distinct emission profile consistent with a different supramolecular assembly: (i) pre‐gel A (HEA/water, 11/89 v/v) showed features characteristic of a predominantly dt‐pseudo[4]rotaxane state, (ii) pre‐gel B (HEA/water, 19/81 v/v) displayed intermediate features corresponding to a mixture of dt‐ and st‐species, and (iii) pre‐gel C (HEA/water, 30/70 v/v) was dominated by monomer‐like pyrene species, consistent with predominantly st‐species (Figure 5d).

Lithium phenyl‐2,4,6‐trimethylbenzoylphosphinate (LAP) photoinitiator was added to each of the pre‐gels and they were photopolymerized with long‐wavelength ultraviolet light (wavelength: 390–500 nm). The goal was to access three topology‐engineered gel networks containing distinct ratios of st‐ to dt‐slide ring crosslinks (see Supporting Information for details). The resulting gel networks were designated as: (i) **2a** ‐ obtained from the dt‐pre‐gel A; (ii) **2b** ‐ obtained from the mixed pre‐gel B; and (iii) **2c** ‐ obtained from the st‐structures in pre‐gel C (Figure 6a). After thorough washing with methanol followed by water to remove unincorporated γ‐CD, all three gels exhibited a high gel fraction (ca. 90 wt%, Figure S15 and see Supporting Information for details).

**FIGURE 6:**
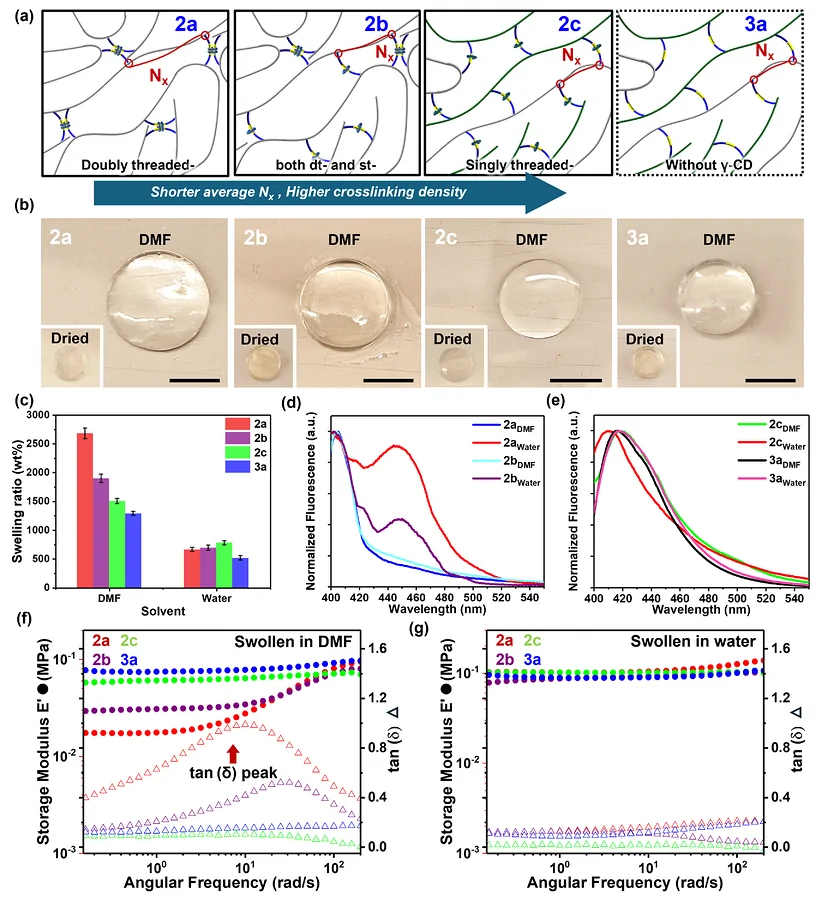
Structural, swelling, optical, and viscoelastic behavior of gel networks with varying topologies. (a) Schematic illustration of **2a**, **2b**, **2c**, and **3a** (no γ‐CD), highlighting differences in average distance between crosslinking points (*N_x_*) and effective crosslinking density. (b) Optical images of dried and DMF‐swollen gels. Scale bars: 10 mm. (c) Swelling ratios of **2a**, **2b**, **2c**, and **3a** in DMF and water (error bars represent standard deviations from five independent measurements). (d) Normalized fluorescence spectra of **2a** and **2b** in DMF and water. (e) Normalized fluorescence spectra of **2c** and **3a** in DMF and water. (f) Frequency‐dependent storage modulus (E′) and loss factor (tan δ) of gels swollen in DMF. (g) Corresponding viscoelastic profiles of gels swollen in water.

To assess the extent of γ‐CD incorporation into each gel network, analysis of the soluble fractions collected during methanol/water washing process was undertaken. The amount of γ‐CD in each soluble fraction was quantified by comparison with an external thymol reference using ^1^H NMR spectroscopy (Figure S16 and see Supporting Information for details). Of the eight equivalents (relative to **1^9^Acr**) of γ‐CD initially present in the pre‐gel formulations, approximately seven equivalents were detected in the collected soluble fractions, corresponding to the uncomplexed excess γ‐CD present prior to gelation. Thus, each of the three gels contains the same ratio of γ‐CD:**1^9^Acr** consistent with the presence of either **1^9^Acr:γ‐CD** or **1^9^Acr_2_:γ‐CD_2_** assemblies in the pre‐gels and, in turn with **2a** being a dt‐slide ring gel, **2c** being a st‐main‐chain polyrotaxane network, while **2b** contained a mixture of both topologies. It is important to note that although these assemblies are noncovalent pseudorotaxane complexes in the pre‐gel state, copolymerization of the acrylate termini of **1^9^Acr** into the poly(HEA) network allows the surrounding polymer chains to act as macromolecular stoppers that prevent dethreading of the interlocked γ‐CDs. Consistent with this, even after 10 days of exposure to dimethylformamide (DMF) at 80°C, no additional γ‐CD was leached out, demonstrating the robust incorporation and topological stability of the gel networks (Figure S17).

Having confirmed the structural stability of the gel networks, their physicochemical properties were next investigated to elucidate the role of the threading topologies. To this end, a pyrene‐containing covalent gel without γ‐CD (**3a**) was synthesized using the same HEA and **1^9^Acr** composition and employed as a control (see Supporting Information for details). The swelling behavior of the four gel networks (**2a**, **2b**, **2c**, and **3a**) was evaluated in both DMF and water (Figure 6b,c). Given the fact that two pyrene moieties can bind more strongly with the γ‐CD in aqueous environments, it was hypothesized that if dt‐slide ring crosslinks were present in **2a**, this would impart solvent‐dependent mobility to the γ‐CD rings (Figure 1b), leading to more significant changes in the gel networks’ physicochemical behavior as well as their mechanical properties, relative to the other gels [73, 74, 75].

All four gel networks exhibited greater swelling in DMF than in water, consistent with more favorable solvent–polymer interactions in the organic medium (Figures 6b,c and S18). While their swelling ratios in water were comparable, the extent of swelling in DMF varied substantially depending on the crosslinking topology. Notably, **2a** displayed the highest swelling ratio in DMF, a behavior consistent with its high content of dt‐slide ring crosslinks. These mobile junctions could enable the network strands to reorganize and extend in response to solvent uptake without accumulating localized stress, owing to their ability to slide along the polymer backbone. **2c** and **3a** showed minimal solvent‐dependent swelling, consistent with the lack of any slide ring crosslinking, while **2b** exhibited intermediate swelling behavior, consistent with it containing a mixture of dt‐slide ring crosslinks and st‐rings.

In addition to ring mobility, differences in crosslinking density arising from the network topology presumably also contribute to the observed swelling trends. **2a** was synthesized using 4000 equivalents of HEA monomers per **1^9^Acr_2_:γ‐CD_2_** crosslinker, leading to long HEA‐based linear segments between adjacent crosslinks. Given that the molecular length of the crosslinker is significantly shorter than the linear HEA segments, the dt‐slide ring crosslinks can be reasonably approximated as point‐like junctions for determining the number of repeat units between crosslinks (*N_x_*) (Figure S19a,b, see Supporting Information for details). In the idealized case, a 2:2 complex could be treated as an *f* = 8 junction. However, because the acrylate groups within the dt‐**1^9^Acr_2_:γ‐CD_2_** crosslinker are in close spatial proximity, two nearby acrylates may become incorporated into the same network strand rather than forming fully independent strand connections. As a result, the effective junction functionality of **2a** is not necessarily fixed at *f* = 8, and lower effective values such as *f* = 6 or even *f* = 4 are also possible depending on the local mode of network incorporation (Figure S19c). Accordingly, an *N_x_* for **2a** was estimated to be ca. 1333 repeat units using an intermediate effective functionality value of f_eff_ = 6 (Figure S19d, see Supporting Information for details). In contrast, the dissociation of **1^9^Acr_2_:γ‐CD_2_** in **2c** produces two equivalents of st‐**1^9^Acr:γ‐CD** junctions (Figure S20a,b). Unlike the dt‐junction in **2a**, whose effective functionality depends on how spatially proximal acrylates are incorporated into the network, each **1^9^Acr:γ‐CD** can be more straightforwardly treated as an *f* = 4 junction because each acrylate is incorporated into a separate polymer strand, giving an estimated *N_x_* of ca. 1000 repeat units (Figure S20c, see Supporting Information for details). Because **3a** was designed as a covalent analogue of **2c** without γ‐CD, it is likewise expected to possess a comparable crosslinking density and a similar *N_x_* value. These *N_x_* estimates provide a relative comparison of effective crosslink density, with **2a** expected to have a lower effective crosslink density than **2c** and **3a**, and **2b** expected to be intermediate, consistent with the reduced swelling ratios observed in DMF for **2c** and **3a** relative to **2a**.

Fluorescence analysis can provide information on the binding state of the pyrene moieties in these gels (Figure 6d,e). Both **2a** and **2b** exhibited solvent‐responsive emission behavior that supports the presence of dt‐slide ring crosslinks. In water, a fluorescence maximum was observed at ca. 450 nm, consistent with the formation of pyrene dimers encapsulated within γ‐CD cavities. Upon solvent exchange to DMF, the emission maximum shifted to ca. 410 nm, which is consistent with isolated pyrene moieties that have dethreaded from the γ‐CD. In contrast, **3a** exhibited negligible solvent‐dependent changes, consistently showing monomeric pyrene emission at ca. 410 nm. For **2c**, a small shift in the emission maximum was observed between DMF and water (Δ*ƛ*_max_ = ca. 6 nm). This can be attributed to changes in the local microenvironment of the pyrene moieties, where they are singly threaded in the γ‐CD cavity in water versus unbound in DMF. The absence of characteristic excimer emission at ca. 450 nm in both **2c** and **3a**, irrespective of solvent, supports the lack of dt‐slide ring crosslinks in these networks.

Dynamic mechanical analysis (DMA) of the fully swollen gels in DMF and water was conducted to investigate their viscoelastic properties (Figure 6f,g). The elastic modulus was measured under compressive axial forces within the linear viscoelastic regime via frequency sweeps, where storage (*E′*) and loss (*E″*) moduli were recorded (Figures 6f and S21). In DMF, the plateau storage modulus (*E′*_plateau_) at angular frequency ω < 1 rad/s decreased systematically with increasing amounts of dt‐slide ring species: **2a_DMF_** (17 kPa) < **2b_DMF_** (30 kPa) < **2c_DMF_** (60 kPa) < **3a_DMF_** (74 kPa). This trend correlates well with the swelling behavior and mesh size of each gel, where the highly swollen **2a_DMF_** is the softest and the minimally swollen **3a_DMF_** is the stiffest. At the molecular level, DMF acts as a good swelling solvent for the poly(HEA) network and weakens the hydrophobic driving force for γ‐CD–pyrene association relative to water. In **2a**, which contains predominantly dt‐slide ring crosslinks, these effects increase the conformational freedom and mobility of the crosslinking junctions, thereby reducing the effective elastic response. By contrast, **2c**, which is dominated by st‐junctions, and **3a**, the covalent control, do not contain the same dt‐slide ring crosslinks and therefore retain higher *E′* values. While these modulus differences could be rationalized by the combined effects of swelling, effective network density, solvent quality, and crosslink topology, the emergence of a *tan* δ peak (*tan* δ = *E″*/*E′*) in **2a_DMF_** provides a distinct rheological signature consistent with an additional sliding‐enabled dissipative mode associated with dt‐slide ring crosslinks [73, 74, 75, 82, 83]. **2a_DMF_** exhibits this *tan* δ peak at 10 rad/s, which is not observed in **2a_water_**, consistent with the dt‐slide ring crosslinks becoming effectively immobilized in the water and functioning more akin to covalent‐like junctions across the timescales studied on account of hydrophobic interactions between the pyrene moieties and γ‐CD cavities (Figure 6g). This solvent‐switchable *tan* δ response supports the activation and suppression of a sliding‐associated dissipative mode in **2a**. Accordingly, **2a_water_** exhibited a markedly higher storage modulus (ca. 100 kPa), consistent with reduced ring mobility and restricted swelling under aqueous conditions. **2b**, on the other hand, showed modest solvent dependence with a weak *tan* δ peak at 30 rad/s in DMF, and both **2c** and **3a** exhibited minimal solvent‐dependent changes in viscoelasticity. In DMF, **2c_DMF_** and **3a_DMF_** showed weak frequency dependence in *E′* and low *tan* δ values across the measured range, without a discernible *tan* δ peak, indicating the absence of a sliding‐associated dissipative mode. Upon exposure to water, **2b_water_**, **2c_water_**, and **3a_water_** displayed viscoelastic responses comparable to **2a_water_**, reflecting their similar swelling ratios and absence (or effective suppression) of crosslink mobility. Complementary stress relaxation measurements were conducted on representative gel networks (**2a** and **2c**) (Figure S22). **2a** exhibited a markedly faster decay of the normalized relaxation modulus, E(t)/E_0_, in DMF than in water, whereas **2c** showed only weak solvent dependence. These results are consistent with an additional solvent‐activated dissipative contribution in **2a_DMF_** associated with the dt‐slide ring crosslink topology. Overall, the combined rheological data demonstrate that **2a** exhibits the most pronounced solvent‐responsive viscoelastic behavior, highlighting how dt‐slide ring crosslinks enable solvent‐dependent switching of dissipative dynamics in topological gel networks.

With a better understanding of the behavior of the gels in place, the next step was to investigate the properties of the dried networks. Although tensile testing of fully swollen gels would provide complementary insight into topology‐dependent large‐strain deformation under solvated conditions, reliable quantitative comparison is complicated by the high solvent content and associated gripping and dimensional instabilities of swollen specimens, particularly under DMF‐swollen conditions. Accordingly, tensile testing was conducted on the four dried networks (complete removal of residual solvent was confirmed by thermogravimetric analysis (TGA), (Figures S23 and 7a). While **2a–2c** have similar chemical composition, at a fixed strain rate of 20% L_0_/min these three networks exhibited distinct stress–strain behaviors and mechanical properties, demonstrating a clear structure–property relationship arising from differences in crosslinking architecture and associated topological mobility (Figure 7b,c and Table S2). **2a** exhibited the highest extensibility (>1050% strain at break), a low initial modulus (ca. 0.67 MPa), and the highest toughness (ca. 3.39 MJ/m^3^). These features are consistent with the sliding motion of the dt‐rings, which is expected to promote a pulley‐like redistribution of stress along the polymer chains, thereby mitigating strain localization. **2b** showed reduced extensibility (ca. 770%) and increased stiffness (ca. 1.15 MPa), consistent with a lower content of dt‐slide ring crosslinks, which reduces sliding‐mediated stress redistribution within the network. **2c** and **3a** ruptured at significantly lower strains (ca. 500% and ca. 430%, respectively) and exhibited higher moduli (ca. 1.72 MPa and ca. 1.32 MPa), reflecting the absence of dt‐slide ring crosslinks, together with a shorter average number of repeat units between junctions (lower *N_x_*). To further examine the influence of effective crosslink density on the tensile response, additional covalent control networks with larger estimated *N_x_* values (**3b**, *N_x_* = ca. 1333, and **3c**, *N_x_* = ca. 2000) were prepared. As *N_x_* increased, these controls exhibited reduced Young's moduli together with increased fracture strain and toughness (Figure S24 and Table S3). Despite its larger estimated *N_x_*, **3c** ruptured at a lower strain and exhibited lower toughness than **2a** (*N_x_* = ca. 1333). These comparisons suggest that the enhanced toughness and delayed network rupture of **2a** cannot be attributed solely to differences in effective crosslink density.

**FIGURE 7:**
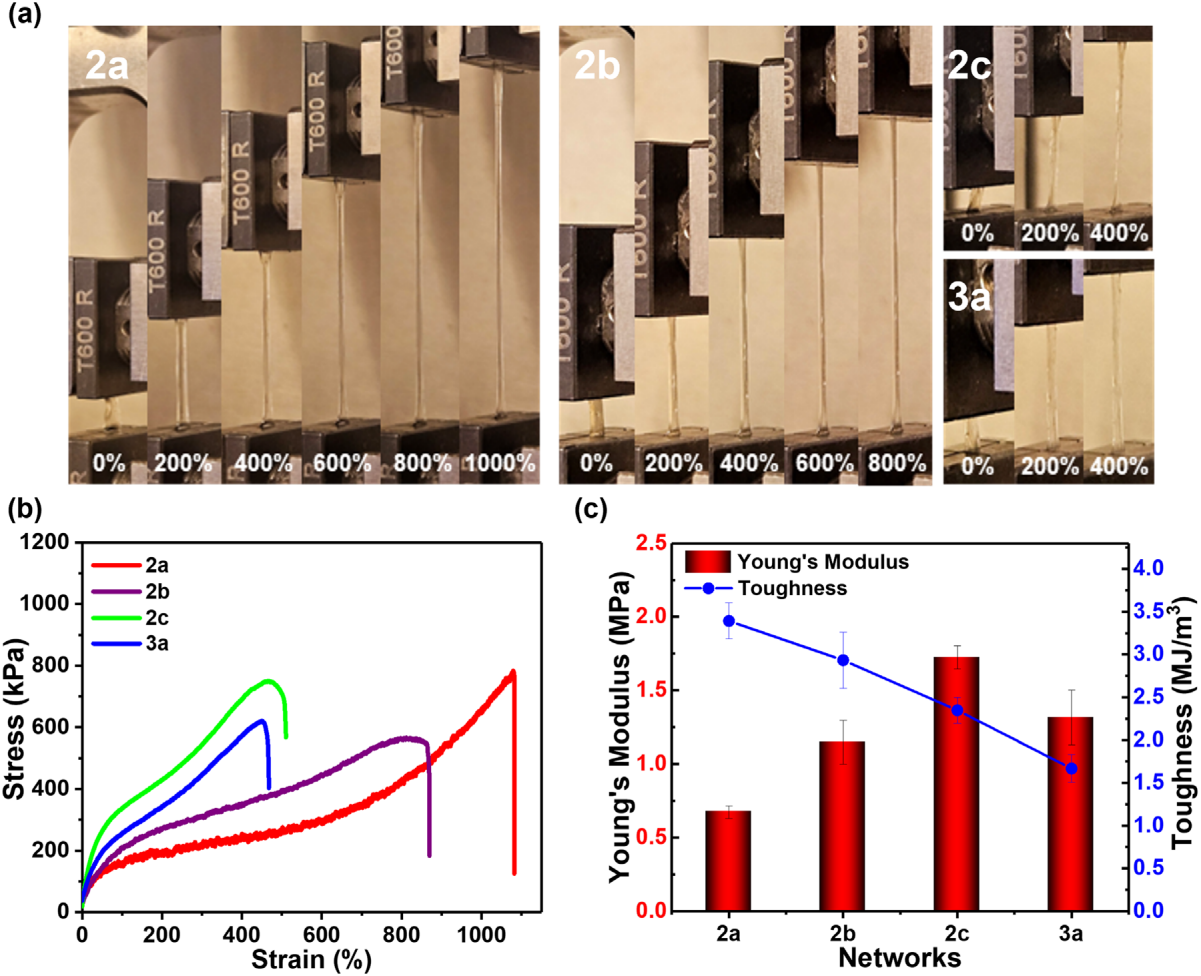
Mechanical performance of the dried networks. (a) Optical images of **2a**, **2b**, **2c**, and **3a** during uniaxial tensile testing. (b) Representative stress–strain curves of each network. (c) Young's modulus (bars, left *y*‐axis) and toughness (line, right *y‐*axis) of each network (see Table S2 for quantitative values). Experiments were performed at a strain rate of 20% L_0_/min.

The effect of crosslinking topology on mechanical performance was further examined by strain rate dependent tensile testing. **2a** did exhibit an earlier onset of strain stiffening and a significant reduction in rupture strain as the deformation rate increased from 20% to 100% L_0_/min (Figure 8a; Movies S1 and S2). This behavior indicates the kinetic suppression of ring mobility at higher deformation rates, which limits stress redistribution and promotes localized stress concentration, leading to higher stress and more brittle‐like rupture. At slower deformation rates, however, the dt‐slide ring crosslinks can retain sufficient mobility to facilitate continuous stress relaxation and uniform deformation, thereby preserving high extensibility. In contrast, both **2c** and **3a** showed minimal rate sensitivity under the same conditions, consistent with their static crosslinking architectures (Figures 8b, and S25; Movies S3 and S4). These results highlight how dt‐slide ring crosslinks can modulate their mechanical response through kinetic regulation of junction dynamics, in contrast to covalent and st‐ring crosslinks.

**FIGURE 8:**
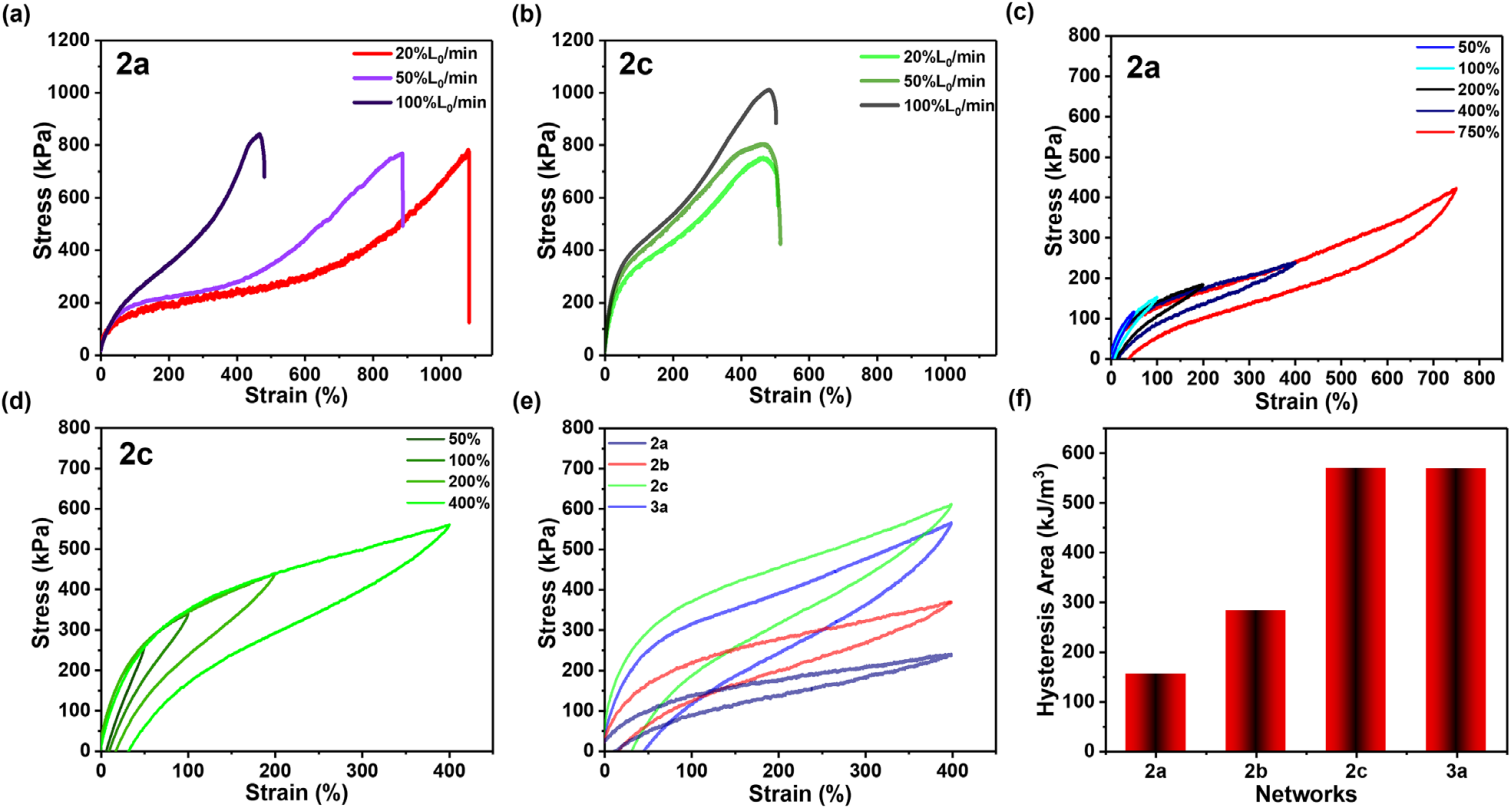
Rate‐dependent and cyclic tensile behavior of the dried networks. (a, b) Strain rate‐dependent tensile behavior of (a) **2a** and (b) **2c** at 20%, 50%, and 100% L_0_/min. (c, d) Cyclic tensile profiles of (c) **2a** and (d) **2c** at increasing strain amplitudes. (e) Direct comparison of the first cyclic loading–unloading curves of dried **2a**–**2c** and **3a** at a fixed maximum strain of 400%. (f) Hysteresis areas of dried **2a**–**2c** and **3a** calculated from the first loading–unloading cycle at 400% maximum strain. Experiments in (c)–(e) were performed at a strain rate of 20% L_0_/min.

To further assess how crosslinking topology influences mechanical resilience under repeated deformation, cyclic tensile tests were performed on the dried networks (Figure 8c,d). For **2a**, the loading‐unloading loops broadened with increasing maximum strain from 50% to 750%, but the increase in hysteresis remained comparatively modest, whereas **2c** showed a more pronounced hysteretic response. For example, at 750% strain, **2a** exhibited a lower hysteretic response than **2c** at 400% strain, despite the substantially higher maximum strain applied to **2a** (Figure S26). For a direct comparison of the hysteretic response under identical deformation conditions, dried **2a**–**2c** and **3a** were subjected to a loading–unloading cycle at a fixed maximum strain of 400% (Figure 8e,f). Among these networks, **2a** showed the lowest hysteresis area. To further quantify the repeated cyclic response under identical deformation conditions, fixed‐strain cyclic tensile tests were performed on **2a** and **2c** at 400% strain for seven consecutive cycles (Figure S27 and Table S4). Under these conditions, **2a** showed lower hysteresis areas and hysteresis ratios than **2c** in both cycle 1 and cycle 7, including a substantially lower hysteresis area after repeated cycling, while the residual strain and recovery efficiency became comparable by cycle 7. Together, these observations are consistent with reversible rearrangement of the dt‐slide ring crosslinks in **2a**. This rearrangement can accommodate cyclic deformation through sliding‐mediated stress redistribution, reduce strain localization, and thereby limit hysteresis. Cyclic tensile tests performed on the same specimens before and after 1 day of rest yielded comparable loading‐unloading loops for both **2a** (750% strain) and **2c** (400% strain) (Figure S28), with no clear indication of permanent damage under these conditions. These results suggest that hysteresis in both networks arises primarily from reversible deformation processes rather than irreversible bond scission. Such reversible deformation processes may also include matrix‐derived contributions, such as segmental friction, relaxation of dangling or elastically inactive chains, and reversible hydrogen‐bonding interactions associated with the hydroxyl‐rich poly(HEA) network. However, because this hydroxyl‐rich poly(HEA) matrix is common to all of the networks, the comparatively lower hysteresis of **2a** under identical cyclic deformation is consistent with the dt‐slide ring crosslink topology modulating the matrix‐derived hysteretic response by redistributing stress during loading–unloading. Taken together with the tensile results to rupture, these observations indicate that the dt‐topology enables a combination of high toughness and comparatively low hysteresis that is not readily achieved within a covalent network architecture.

## 3 Conclusion

A modular strategy was developed for constructing topology‐engineered gel networks by leveraging doubly threaded (dt‐) pseudo[4]rotaxane crosslinkers formed through hydrophobic inclusion complexation between γ‐cyclodextrin and 1,6‐bisarylpyrene‐based threads functionalized with polymerizable acrylate moieties. By precisely tuning the supramolecular assembly in the pre‐gel from predominantly dt‐ to singly threaded (st‐) states, a series of compositionally similar but topologically distinct networks (**2a–2c**) could be prepared along with a control non‐threaded covalent network **3a**. Comprehensive characterization of the swollen networks **2a**‐**2c** and **3a** indicates that incorporation of dt‐slide ring crosslinks is a key factor in dictating macroscopic material properties. In the swollen state, the dt‐network **2a** exhibited pronounced solvent‐responsive fluorescence and viscoelastic signatures, whereas the st‐**2c** and non‐threaded **3a** networks showed minimal solvent‐dependent responses. Tensile and cyclic tests on the corresponding dried networks (**2a–2c** and **3a**) further revealed that dt‐junctions in **2a** enable high extensibility, strain‐rate‐dependent stiffening, and high toughness together with comparatively low hysteresis relative to networks lacking such architectures. Importantly, the strain‐rate dependence supports a kinetic regulation of junction mobility, which determines whether the junctions enable sliding‐associated stress relaxation or promote stress localization. In contrast, **2c** and **3a** displayed limited extensibility and more pronounced hysteresis under cyclic deformation, underscoring the critical role of topological mobility in determining the mechanical response. Together, these findings establish a direct structure–property relationship governed by crosslinking topology and demonstrate topological design as a powerful strategy for engineering mechanically robust and stimulus‐responsive polymer networks. This modular platform provides a versatile foundation for next‐generation soft materials across applications including flexible electronics, soft robotics, polymer binders, and biomedical devices that demand materials combining robustness with programmable adaptability.

## Author Contributions

**Jongwon Oh**: conceptualization, methodology, writing – review and editing, writing – original draft, formal analysis, investigation, and data curation. **Heyi Liang**: writing – review and editing, investigation, software, and data curation. **Natsumi Nitta**: writing – review and editing, investigation. **Jongmin Kim**: investigation, writing – review and editing. **Yuan Tian**: writing – review and editing, investigation, and software. **Guancen Liu**: writing – review and editing, investigation. **Jerald E. Hertzog**: investigation, writing – review and editing. **Juan J. de Pablo**: project administration, supervision, resources, and writing – review and editing. **Stuart J. Rowan**: conceptualization, writing – review and editing, project administration, supervision, and resources.

## Conflicts of Interest

The authors declare no conflicts of interest.

## Supporting information



Synthesis and characterization of monomers, pseudorotaxane crosslinkers, pre‐gels, gels, and dried networks are detailed in the Supporting Information. The authors have cited additional references within the Supporting Information.**Supporting File 1**: anie74248‐sup‐0001‐SuppMat.pdf.

**Supporting File 2**: anie74248‐sup‐0002‐VideoS1‐S4.zip.

## Data Availability

The data that support the findings of this study are available on request from the corresponding author. The data are not publicly available due to privacy or ethical restrictions.

## References

[1] W. W. Graessley , in Synthesis and Degradation Rheology and Extrusion (Springer, 2005), 67–117, 10.1007/BFb0038532.

[2] T. Kaiser , “Highly Crosslinked Polymers,” Progress in Polymer Science 14 (1989): 373–450, 10.1016/0079-6700(89)90007-5.

[3] X. Zhao , X. Chen , H. Yuk , S. Lin , X. Liu , and G. Parada , “Soft Materials by Design: Unconventional Polymer Networks Give Extreme Properties,” Chemical Reviews 121 (2021): 4309–4372, 10.1021/acs.chemrev.0c01088. PMC9217625 33844906

[4] L. Debertrand , J. Zhao , C. Creton , and T. Narita , “Swelling and Mechanical Properties of Polyacrylamide‐Derivative Dual‐Crosslink Hydrogels Having Metal–Ligand Coordination Bonds as Transient Crosslinks,” Gels 7 (2021): 72, 10.3390/gels7020072. PMC8293112 34203901

[5] T. Katashima , “Rheological Studies on Polymer Networks With Static and Dynamic Crosslinks,” Polymer Journal 53 (2021): 1073–1082, 10.1038/s41428-021-00505-y.

[6] V. A. Kong , T. A. Staunton , and J. E. Laaser , “Effect of Cross‐Link Homogeneity on the High‐Strain Behavior of Elastic Polymer Networks,” Macromolecules 57 (2024): 4670–4679, 10.1021/acs.macromol.3c02565. PMC11140753 38827963

[7] C. Norioka , Y. Inamoto , C. Hajime , A. Kawamura , and T. Miyata , “A Universal Method to Easily Design Tough and Stretchable Hydrogels,” NPG Asia Materials 13 (2021): 34, 10.1038/s41427-021-00302-2.

[8] S. Pruksawan , J. W. R. Lim , Y. L. Lee , et al., “Enhancing Hydrogel Toughness by Uniform Cross‐Linking Using Modified Polyhedral Oligomeric Silsesquioxane,” Communications Materials 4 (2023): 75, 10.1038/s43246-023-00402-2.

[9] D. Zhong , Z. Wang , J. Xu , et al., “A Strategy for Tough and Fatigue‐Resistant Hydrogels via Loose Cross‐Linking and Dense Dehydration‐Induced Entanglements,” Nature Communications 15 (2024): 5896, 10.1038/s41467-024-50364-3. PMC11246433 39003311

[10] C. W. H. Rajawasam , O. J. Dodo , M. A. S. N. Weerasinghe , et al., “Educational Series: Characterizing Crosslinked Polymer Networks,” Polymer Chemistry 15 (2024): 219–247, 10.1039/D3PY00914A.

[11] J. Song , S. Chen , L. Sun , et al., “Mechanically and Electronically Robust Transparent Organohydrogel Fibers,” Advanced Materials 32 (2020): 1906994, 10.1002/adma.201906994. 31957099

[12] H.‐Q. Peng , W. Zhu , W.‐J. Guo , et al., “Supramolecular Polymers: Recent Advances Based on the Types of Underlying Interactions,” Progress in Polymer Science 137 (2023): 101635, 10.1016/j.progpolymsci.2022.101635.

[13] S. Seiffert and J. Sprakel , “Physical Chemistry of Supramolecular Polymer Networks,” Chemical Society Reviews 41 (2012): 909–930, 10.1039/C1CS15191F. 21909565

[14] R. J. Wojtecki , M. A. Meador , and S. J. Rowan , “Using the Dynamic Bond to Access Macroscopically Responsive Structurally Dynamic Polymers,” Nature Materials 10 (2011): 14–27, 10.1038/nmat2891. 21157495

[15] V. Zhang , B. Kang , J. V. Accardo , and J. A. Kalow , “Structure–Reactivity–Property Relationships in Covalent Adaptable Networks,” Journal of the American Chemical Society 144 (2022): 22358–22377, 10.1021/jacs.2c08104. PMC9812368 36445040

[16] N. Zheng , Y. Xu , Q. Zhao , and T. Xie , “Dynamic Covalent Polymer Networks: A Molecular Platform for Designing Functions Beyond Chemical Recycling and Self‐Healing,” Chemical Reviews 121 (2021): 1716–1745, 10.1021/acs.chemrev.0c00938. 33393759

[17] G. Armstrong and M. Buggy , “Hydrogen‐Bonded Supramolecular Polymers: A Literature Review,” Journal of Materials Science 40 (2005): 547–559, 10.1007/s10853-005-6288-7.

[18] L. M. de Espinosa , G. L. Fiore , C. Weder , E. Johan Foster , and Y. C. Simon , “Healable Supramolecular Polymer Solids,” Progress in Polymer Science 49‐50 (2015): 60–78, 10.1016/j.progpolymsci.2015.04.003.

[19] J. K. Hirschberg , L. Brunsveld , A. Ramzi , J. A. Vekemans , R. P. Sijbesma , and E. Meijer , “Helical Self‐Assembled Polymers From Cooperative Stacking of Hydrogen‐Bonded Pairs,” Nature 407 (2000): 167–170, 10.1038/35025027. 11001050

[20] S. Sivakova , D. A. Bohnsack , M. E. Mackay , P. Suwanmala , and S. J. Rowan , “Utilization of a Combination of Weak Hydrogen‐Bonding Interactions and Phase Segregation to Yield Highly Thermosensitive Supramolecular Polymers,” Journal of the American Chemical Society 127 (2005): 18202–18211, 10.1021/ja055245w. 16366573

[21] M. Burnworth , L. Tang , J. R. Kumpfer , et al., “Optically Healable Supramolecular Polymers,” Nature 472 (2011): 334–337, 10.1038/nature09963. 21512571

[22] L. He , Y. Jiang , J. Wei , et al., “Highly Robust Supramolecular Polymer Networks Crosslinked by a Tiny Amount of Metallacycles,” Nature Communications 15 (2024): 3050, 10.1038/s41467-024-47333-1. PMC11004166 38594237

[23] J. R. Kumpfer , J. J. Wie , J. P. Swanson , F. L. Beyer , M. E. Mackay , and S. J. Rowan , “Influence of Metal Ion and Polymer Core on the Melt Rheology of Metallosupramolecular Films,” Macromolecules 45 (2012): 473–480, 10.1021/ma201659d.

[24] J. Park , J. Park , J. Lee , C. Lim , and D. W. Lee , “Size Compatibility and Concentration Dependent Supramolecular Host–Guest Interactions at Interfaces,” Nature Communications 13 (2022): 112, 10.1038/s41467-021-27659-w. PMC8748952 35013244

[25] G. Sinawang , M. Osaki , Y. Takashima , H. Yamaguchi , and A. Harada , “Supramolecular Self‐Healing Materials From Non‐Covalent Cross‐Linking Host–Guest Interactions,” Chemical Communications 56 (2020): 4381–4395, 10.1039/D0CC00672F. 32249859

[26] X. Tang , Z. Huang , H. Chen , Y. Kang , J. F. Xu , and X. Zhang , “Supramolecularly Catalyzed Polymerization: From Consecutive Dimerization to Polymerization,” Angewandte Chemie International Edition 57 (2018): 8545–8549, 10.1002/anie.201803749. 29756289

[27] B. Zheng , F. Wang , S. Dong , and F. Huang , “Supramolecular Polymers Constructed by Crown Ether‐Based Molecular Recognition,” Chemical Society Reviews 41 (2012): 1621–1636, 10.1039/C1CS15220C. 22012256

[28] T. Fukushima , K. Tamaki , A. Isobe , et al., “Diarylethene‐Powered Light‐Induced Folding of Supramolecular Polymers,” Journal of the American Chemical Society 143 (2021): 5845–5854, 10.1021/jacs.1c00592. 33755463

[29] F. Wang , R. Liao , and F. Wang , “Pathway Control of π‐Conjugated Supramolecular Polymers by Incorporating Donor‐Acceptor Functionality,” Angewandte Chemie International Edition 62 (2023): e202305827, 10.1002/anie.202305827. 37431813

[30] Y. S. Wong , M. Ng , M. C. Yeung , and V. W. Yam , “Platinum(II)‐Based Host–Guest Coordination‐Driven Supramolecular Co‐Assembly Assisted by Pt···Pt and π–π Stacking Interactions: A Dual‐Selective Luminescence Sensor for Cations and Anions,” Journal of the American Chemical Society 143 (2021): 973–982, 10.1021/jacs.0c11162. 33403853

[31] D. J. Lundberg , C. M. Brown , E. O. Bobylev , et al., “Nested Non‐Covalent Interactions Expand the Functions of Supramolecular Polymer Networks,” Nature Communications 15 (2024): 3951, 10.1038/s41467-024-47666-x. PMC11087514 38730254

[32] D. Xu , B. D. Olsen , and S. L. Craig , “Relaxation Dynamics of Supramolecular Polymer Networks With Mixed Cross‐Linkers,” Journal of Rheology 66 (2022): 1193–1201, 10.1122/8.0000421.

[33] T. Yan , K. Schröter , F. Herbst , W. H. Binder , and T. Thurn‐Albrecht , “Unveiling the Molecular Mechanism of Self‐Healing in a Telechelic, Supramolecular Polymer Network,” Scientific Reports 6 (2016): 32356, 10.1038/srep32356. PMC5007665 27581380

[34] D. Görl , S. Haraguchi , Y. Hryshunin , et al., “Supramolecular Modification of Sustainable High‐Molar‐Mass Polymers for Improved Processing and Performance,” Nature Communications 16 (2025): 217, 10.1038/s41467-024-55166-1. PMC11696020 39746956

[35] J. Sautaux , F. Marx , I. Gunkel , C. Weder , and S. Schrettl , “Mechanically Robust Supramolecular Polymer Co‐Assemblies,” Nature Communications 13 (2022): 356, 10.1038/s41467-022-28017-0. PMC8766479 35042887

[36] P. Cordier , F. Tournilhac , C. Soulié‐Ziakovic , and L. Leibler , “Self‐Healing and Thermoreversible Rubber From Supramolecular Assembly,” Nature 451 (2008): 977–980, 10.1038/nature06669. 18288191

[37] J. C. Lai , X. Y. Jia , D. P. Wang , et al., “Thermodynamically Stable Whilst Kinetically Labile Coordination Bonds Lead to Strong and Tough Self‐Healing Polymers,” Nature Communications 10 (2019): 1164, 10.1038/s41467-019-09130-z. PMC6411951 30858371

[38] L. N. Neumann , E. Oveisi , A. Petzold , et al., “Dynamics and Healing Behavior of Metallosupramolecular Polymers,” Science Advances 7 (2021): eabe4154, 10.1126/sciadv.abe4154. PMC8081362 33910908

[39] Q. Zhang , C.‐Y. Shi , D.‐H. Qu , Y.‐T. Long , B. L. Feringa , and H. Tian , “Exploring a Naturally Tailored Small Molecule for Stretchable, Self‐Healing, and Adhesive Supramolecular Polymers,” Science Advances 4 (2018): eaat8192, 10.1126/sciadv.aat8192. PMC6063538 30062126

[40] C. J. Bruns and J. F. Stoddart , in The Nature of the Mechanical Bond: From Molecules to Machines (Wiley, 2016), 10.1002/9781119044123.

[41] J. P. Sauvage , “From Chemical Topology to Molecular Machines (Nobel Lecture),” Angewandte Chemie International Edition 56 (2017): 11080–11093, 10.1002/anie.201702992. 28632333

[42] J. F. Stoddart , “The Chemistry of the Mechanical Bond,” Chemical Society Reviews 38 (2009): 1802, 10.1039/b819333a. 19587969

[43] J. F. Stoddart , “Mechanically Interlocked Molecules (MIMs)—Molecular Shuttles, Switches, and Machines (Nobel Lecture),” Angewandte Chemie International Edition 56 (2017): 11094–11125, 10.1002/anie.201703216. 28815900

[44] C. P. Collier , G. Mattersteig , E. W. Wong , et al., “A [2]Catenane‐Based Solid State Electronically Reconfigurable Switch,” Science 289 (2000): 1172–1175, 10.1126/science.289.5482.1172. 10947980

[45] R. S. Forgan , J. J. Gassensmith , D. B. Cordes , et al., “Self‐Assembly of a [2]Pseudorota[3]catenane in Water,” Journal of the American Chemical Society 134 (2012): 17007–17010, 10.1021/ja3085115. 22998143

[46] G. Gil‐Ramírez , D. A. Leigh , and A. J. Stephens , “Catenanes: Fifty Years of Molecular Links,” Angewandte Chemie International Edition 54 (2015): 6110–6150, 10.1002/anie.201411619. PMC4515087 25951013

[47] S. J. Cantrill , S. J. Rowan , and J. F. Stoddart , “Rotaxane Formation Under Thermodynamic Control,” Organic Letters 1 (1999): 1363–1366, 10.1021/ol990966j.

[48] J. J. Danon , D. A. Leigh , P. R. McGonigal , J. W. Ward , and J. Wu , “Triply Threaded [4]Rotaxanes,” Journal of the American Chemical Society 138 (2016): 12643–12647, 10.1021/jacs.6b07733. 27570875

[49] S. J. Rowan , S. J. Cantrill , and J. F. Stoddart , “Triphenylphosphonium‐Stoppered [2]Rotaxanes,” Organic Letters 1 (1999): 129–132, 10.1021/ol990049d.

[50] J. A. Wisner , P. D. Beer , M. G. Drew , and M. R. Sambrook , “Anion‐Templated Rotaxane Formation,” Journal of the American Chemical Society 124 (2002): 12469–12476, 10.1021/ja027519a. 12381188

[51] M. I. Bardot , C. W. Weyhrich , Z. Shi , et al., “Mechanically Interlocked Two‐Dimensional Polymers,” Science 387 (2025): 264–269, 10.1126/science.ads4968. 39818896

[52] L. F. Hart , J. E. Hertzog , P. M. Rauscher , B. W. Rawe , M. M. Tranquilli , and S. J. Rowan , “Material Properties and Applications of Mechanically Interlocked Polymers,” Nature Reviews Materials 6 (2021): 508–530, 10.1038/s41578-021-00278-z.

[53] Y. Wang , Z. Zhang , H. Zhang , et al., “Mechanically Interlocked [an]Daisy Chain Networks,” Chemistry 9 (2023): 2206–2221, 10.1016/j.chempr.2023.04.005.

[54] M. Yang , S. Chen , Z. Zhang , et al., “Stimuli‐Responsive Mechanically Interlocked Polymer Wrinkles,” Nature Communications 15 (2024): 5760, 10.1038/s41467-024-49750-8. PMC11233622 38982046

[55] Z. Zhang , J. Zhao , Z. Guo , et al., “Mechanically Interlocked Networks Cross‐Linked by a Molecular Necklace,” Nature Communications 13 (2022): 1393, 10.1038/s41467-022-29141-7. PMC8927564 35296669

[56] S. W. Zhou , D. Zhou , R. Gu , C. S. Ma , C. Yu , and D. H. Qu , “Mechanically Interlocked [c2]Daisy Chain Backbone Enabling Advanced Shape‐Memory Polymeric Materials,” Nature Communications 15 (2024): 1690, 10.1038/s41467-024-45980-y. PMC10894290 38402228

[57] Y. Okumura and K. Ito , “The Polyrotaxane Gel: A Topological Gel by Figure‐of‐Eight Cross‐links,” Advanced Materials 13 (2001): 485–487, 10.1002/1521-4095(200104)13:7<485::AID-ADMA485>3.0.CO;2-T.

[58] K. Ito , “Novel Entropic Elasticity of Polymeric Materials: Why is Slide‐Ring Gel So Soft?,” Polymer Journal 44 (2012): 38–41, 10.1038/pj.2011.85.

[59] C. Liu , H. Kadono , K. Mayumi , K. Kato , H. Yokoyama , and K. Ito , “Unusual Fracture Behavior of Slide‐Ring Gels With Movable Cross‐Links,” ACS Macro Letters 6 (2017): 1409–1413, 10.1021/acsmacrolett.7b00729. 35650803

[60] L. Jiang , C. Liu , K. Mayumi , K. Kato , H. Yokoyama , and K. Ito , “Highly Stretchable and Instantly Recoverable Slide‐Ring Gels Consisting of Enzymatically Synthesized Polyrotaxane With Low Host Coverage,” Chemistry of Materials 30 (2018): 5013–5019, 10.1021/acs.chemmater.8b01208.

[61] Y. Yasuda , T. Masumoto , K. Mayumi , et al., “Molecular Dynamics Simulation and Theoretical Model of Elasticity in Slide‐Ring Gels,” ACS Macro Letters 9 (2020): 1280–1285, 10.1021/acsmacrolett.0c00194. 35638618

[62] S. Ando and K. Ito , “Recent Progress and Future Perspective in Slide‐Ring Based Polymeric Materials,” Macromolecules 58 (2025): 2157–2177, 10.1021/acs.macromol.4c02021.

[63] A. Bin Imran , K. Esaki , H. Gotoh , et al., “Extremely Stretchable Thermosensitive Hydrogels by Introducing Slide‐Ring Polyrotaxane Cross‐Linkers and Ionic Groups Into the Polymer Network,” Nature Communications 5 (2014): 5124, 10.1038/ncomms6124. PMC4214411 25296246

[64] H. Gotoh , C. Liu , A. B. Imran , et al., “Optically Transparent, High‐Toughness Elastomer Using a Polyrotaxane Cross‐Linker as a Molecular Pulley,” Science Advances 4 (2018): eaat7629, 10.1126/sciadv.aat7629. PMC6184743 30333989

[65] J. Sawada , D. Aoki , H. Otsuka , and T. Takata , “A Guiding Principle for Strengthening Crosslinked Polymers: Synthesis and Application of Mobility‐Controlling Rotaxane Crosslinkers,” Angewandte Chemie International Edition 58 (2019): 2765–2768, 10.1002/anie.201813439. 30600883

[66] Y. Akae , J. Sawada , K. Nakajima , and T. Takata , “The Effect of the Axle End Structure and Number of Through‐Space Bonds on the Properties of Rotaxane Crosslinked Polymers,” Angewandte Chemie International Edition 62 (2023): e202303341, 10.1002/anie.202303341. 37158760

[67] S. Liu , T. Hayashi , M. Hara , T. Seki , K. Ito , and Y. Takeoka , “Optimal Conditions for the Use of Polyrotaxane as a Cross‐Linker in Preparing Elastomers With High Toughnesses,” Polymer Journal 56 (2024): 589–598, 10.1038/s41428-024-00896-8.

[68] J. E. Hertzog , G. Liu , B. W. Rawe , et al., “Balancing Ring and Stopper Group Size to Control the Stability of Doubly Threaded [3]Rotaxanes,” Organic & Biomolecular Chemistry 21 (2023): 6969–6978, 10.1039/D3OB01123B. 37581904

[69] J. E. Hertzog , V. J. Maddi , L. F. Hart , et al., “Metastable Doubly Threaded [3]Rotaxanes With a Large Macrocycle,” Chemical Science 13 (2022): 5333–5344, 10.1039/D2SC01486F. PMC9093191 35655545

[70] S. Huang , Z. Wang , J. Wu , et al., “A Molecular Sheaf: Doubly Threaded [6]Rotaxane,” Chemical Communications 60 (2024): 5622–5625, 10.1039/D4CC00178H. 38715529

[71] M. Tang , D. Zheng , J. Samanta , et al., “Reinforced Double‐Threaded Slide‐Ring Networks for Accelerated Hydrogel Discovery and 3D Printing,” Chemistry 9 (2023): 3515–3531, 10.1016/j.chempr.2023.07.020.

[72] Q. Wu , P. M. Rauscher , X. Lang , et al., “Poly[*n*]catenanes: Synthesis of Molecular Interlocked Chains,” Science 358 (2017): 1434–1439, 10.1126/science.aap7675. 29192134

[73] J. Oh , G. Liu , H. Kim , J. E. Hertzog , N. Nitta , and S. J. Rowan , “Exploring the Impact of Ring Mobility on the Macroscopic Properties of Doubly Threaded Slide‐Ring Gel Networks,” Angewandte Chemie International Edition 63 (2024): e202411172, 10.1002/anie.202411172. 39158508

[74] L. F. Hart , W. R. Lenart , J. E. Hertzog , et al., “Doubly Threaded Slide‐Ring Polycatenane Networks,” Journal of the American Chemical Society 145 (2023): 12315–12323, 10.1021/jacs.3c02837. 37227296

[75] G. Liu , J. Oh , Y. Tian , et al., “Structure–Property Relationships of Responsive Doubly‐Threaded Slide‐Ring Polycatenane Networks,” Chemical Science 16 (2025): 19192–19204, 10.1039/D5SC05459A. PMC12442903 40969167

[76] A. Munoz de la Pena , T. T. Ndou , J. B. Zung , K. L. Greene , D. H. Live , and I. M. Warner , “Alcohol Size as a Factor in the Ternary Complexes Formed With Pyrene and Beta‐Cyclodextrin,” Journal of the American Chemical Society 113 (1991): 1572–1577, 10.1021/ja00005a019.

[77] S. Hashimoto and J. Thomas , “Fluorescence Study of Pyrene and Naphthalene in Cyclodextrin–Amphiphile Complex Systems,” Journal of the American Chemical Society 107 (1985): 4655–4662, 10.1021/ja00302a010.

[78] W. Xu , J. Demas , B. DeGraff , and M. Whaley , “Interactions of Pyrene With Cyclodextrins and Polymeric Cyclodextrins,” Journal of Physical Chemistry 97 (1993): 6546–6554, 10.1021/j100126a035.

[79] M. Inouye , K. Hayashi , Y. Yonenaga , et al., “A Doubly Alkynylpyrene‐Threaded [4]Rotaxane That Exhibits Strong Circularly Polarized Luminescence From the Spatially Restricted Excimer,” Angewandte Chemie International Edition 53 (2014): 14392–14396, 10.1002/anie.201408193. 25349047

[80] K. Kano , H. Matsumoto , S. Hashimoto , M. Sisido , and Y. Imanishi , “A Chiral Pyrene Excimer in .Gamma‐Cyclodextrin Cavity,” Journal of the American Chemical Society 107 (1985): 6117–6118, 10.1021/ja00307a055.

[81] K. Nishioki , A. Kimura , Y. Ohishi , et al., “High‐Yield Syntheses of Doubly 2,7‐Dialkynylpyrene‐Threaded Photostable [8]Rotaxanes Exhibiting Extremely Bright Circularly Polarized Luminescence,” Chemical Science 16 (2025): 20414–20421, 10.1039/D5SC06304C. PMC12495304 41050579

[82] G. Fleury , G. Schlatter , C. Brochon , et al., “Topological Polymer Networks With Sliding Cross‐Link Points: The “Sliding Gels”. Relationship Between Their Molecular Structure and the Viscoelastic as Well as the Swelling Properties,” Macromolecules 40 (2007): 535–543, 10.1021/ma0605043.

[83] K. Kato , T. Yasuda , and K. Ito , “Viscoelastic Properties of Slide‐Ring Gels Reflecting Sliding Dynamics of Partial Chains and Entropy of Ring Components,” Macromolecules 46 (2013): 310–316, 10.1021/ma3021135.

