# Supplementary material for: Impact of Threading State on the Mechanics of γ‐Cyclodextrin–Pyrene Gels
Source: Angew Chem Int Ed Engl. 2026 Aug 22;65(42):e1958506. doi: 10.1002/anie.1958506 (PMC13653618; doi:10.1002/anie.1958506)
Supplement: Supplementary file 1 [file ANIE-65-e1958506-s001.pdf]

Impact of Threading State on the Mechanics of γ-Cyclodextrin–Pyrene Gels

Authors: Jongwon Oh[a], Heyi Liang[a], Natsumi Nitta[a], Jongmin Kim[b], Yuan Tian[ac], Guancen Liu[b], Jerald E. Hertzog[a], Juan J. de Pablo*[acde], and Stuart J. Rowan*[abf]

a Pritzker School of Molecular Engineering, University of Chicago, Chicago, IL 60637, USA
b Department of Chemistry, University of Chicago, Chicago, IL 60637, USA
c Department of Chemical and Biomolecular Engineering, Tandon School of Engineering, New York University, Brooklyn, NY 11201, USA
d Department of Computer Science, Courant Institute of Mathematics, Computing, and Data Sciences, New York University, New York, NY 10012, USA
e Department of Physics, New York University, New York, NY 10003, USA
f Chemical Science and Engineering Division and Center for Molecular Engineering, Argonne National Laboratory, 9700 S. Cass Ave., Lemont, IL 60434, USA

Materials and methods<br>• Materials<br>• Instruments and methods<br>• Computational details	Pages 2-6
Synthesis of acrylate end-capped pyrene thread (**1⁹Acr**)<br>• Synthesis of mono-tosylated nonaethylene glycol<br>• Synthesis of 1,6-di(4'-hydroxyphenyl)pyrene (**PyPh-OH**)<br>• Synthesis of photo-curable pyrene thread (**1⁹Acr**)	Pages 7–9<br>•Fig. S1<br>•Fig. S2<br>•Fig. S3
Host-guest complexation experiments<br>• Fluorescence titration of γ-CD into **1⁹Acr** in aqueous solution<br>• NMR titration of γ-CD into **1⁹Acr** in aqueous solution<br>• Comparison of NOESY spectrum between free **1⁹Acr** and **1⁹Acr/γ-CD** complex<br>• Simulated NOESY spectra for free **1⁹Acr** and **1⁹Acr₂:γ-CD₂** complex	Pages 9–13<br><br>•Figs. S4–8<br>•Fig. S9<br><br>•Fig. S10
Fluorescence titration of pregel with varying HEA/water ratios	Page 13
Preparation of **1⁹Acr/γ-CD** in DMSO-d_6/D_2O mixtures for NMR analysis<br>• Fluorescence and NMR spectra of **1⁹Acr/γ-CD** in DMSO-d_6/D_2O mixtures<br>• NOESY measurements of **1⁹Acr/γ-CD** in DMSO-d_6/D_2O mixtures	Pages 13–15<br>•Figs. S11–12<br><br>•Figs. S13–14
Preparation of representative pregel formulations (pregel A-C)	Page 16<br>•Table S1
Fabrication of topology engineered gel networks<br>• Gel fraction of topology engineered networks	Pages 16–18<br>•Fig. S15
Characterization of these gel networks<br>• Quantification of γ-CD content in topological gel networks<br>• Swelling behavior of these topological gel networks<br>• Crosslinking density analysis<br>• Viscoelastic properties of these gel networks<br>• Characterization of corresponding dried networks	Pages 18–27<br>•Figs. S16–17<br>•Fig. S18<br>•Figs. S19–20<br>•Figs. S21–22<br>•Figs. S23–28, Tables S2–4
Captions for movies	Page 27
References	Pages 27–28

Materials and methods

Materials

All chemicals were purchased from Sigma-Aldrich and used without further purification unless otherwise noted. Chromatography solvents were obtained from Fisher Scientific. Thymol standard (TraceCERT®) was acquired from Sigma-Aldrich and stored in a nitrogen-filled desiccator. Nonaethylene glycol and 1H,1H,2H,2H-perfluoro octyl trichlorosilane (PFOTS) were purchased from AA Blocks. Deuterated solvents were purchased from ACROS Organics and Sigma-Aldrich. Tetrahydrofuran (THF) was dried over sodium/benzophenone, dichloromethane was distilled over calcium hydride, and *N*,*N*-dimethylformamide (DMF) was dried over activated 4 Å molecular sieves. The synthesis of 1,6-di(4'-hydroxyphenyl)pyrene (**PyPh-OH**) was conducted according to previously reported literature procedure[9]. All synthesized components were stored in a freezer at -40 °C prior to use.

Instruments and methods

Nuclear magnetic resonance spectroscopy (NMR): NMR experiments were conducted utilizing a Bruker Ascend AVANCE III 500 MHz spectrometer (126 MHz for ^{13}C), a Bruker AVANCE II+ 500 MHz spectrometer (126 MHz for ^{13}C), or a Bruker DRX 400 MHz spectrometer (101 MHz for ^{13}C) at the University of Chicago Chemistry Department's NMR Facility. ^{1}H NMR spectra were referenced to the residual protonated solvent signal, and ^{13}C NMR spectra were referenced to the deuterated solvent carbon resonance signal.

Diffusion-ordered NMR spectroscopy (DOSY NMR): DOSY NMR experiments were carried out with dilute solutions in 5 mm NMR tubes at 25 °C using the 2D Bruker pulse program dstebpgp3s[1,2], which incorporates a double stimulated echo for convection compensation, a longitudinal echo delay, bipolar gradient pulses, and three spoil gradients. The corresponding 1D pulse sequence dstebpgp3d1d was employed to optimize the parameters D20 ("big delta", the major diffusion delay) and P30 ("little delta", the diffusion gradient length), following the manufacturer-recommended method[3]. Data acquisition involved a linear array of 32 diffusion gradient strengths (GPZ6 values) from 5% to 95%.

Matrix-assisted laser desorption/ionization time-of-flight mass spectrometry (MALDI-TOF MS): MALDI-TOF MS analyses were performed on a Bruker autoflex max MALDI-TOF spectrometer at the University of Chicago Mass Spectrometry Facility in linear mode using a dithranol as the matrix and sodium trifluoroacetate as the ionizer (or no ionizer).

Fluorescence Spectrometry: Fluorescence spectra were measured using a Duetta 3-in-1 spectrofluorometer (HORIBA Scientific) with an excitation wavelength of 375 nm. Gels (fully swollen in DMF or water) were cut into a circular shape (diameter: 10 mm) and placed onto a quartz glass slide.

Oscillatory Compression Rheology: Oscillatory compression rheology experiments were performed using a TA Instruments RSA G2 with the 12 mm compression plate geometry. Gel samples (fully swollen in DMF or water) were cut using a 10 mm die punch and preloaded with 0.20 N to ensure uniform contact between the sample and plates. Frequency sweeps were performed from 1000 rad/s to 0.05 rad/s with a strain amplitude of 1%.

Compressive Stress Relaxation: Compressive stress relaxation experiments were carried out using a TA Instruments RSA G2 with the 12 mm compression plate geometry. Gel samples (fully swollen in DMF or water) were cut using a 10 mm die punch and preloaded with 0.20 N to ensure uniform contact between the sample and plates. A step compressive strain of 4% was applied.

Tensile testing: Tensile testing experiments were performed on a Zwick Roell zwickiLine Z0.5 materials testing machine equipped with a 200 N load cell at room temperature (25 °C) under ambient conditions. Rectangular specimens were cut from bulk films with dimensions of 3.0 mm width and 12.0 mm overall length. The thickness of each specimen was measured using digital calipers prior to testing. Films were loaded between metal tensile grips with minimal handling to avoid pre-stress. Tensile testing measurements were performed at a strain rate of 20% L_0/min until failure. Young's modulus was determined from the linear elastic region at low strain (< 1%).

Strain rate-dependent tensile testing: The same experimental conditions as described above were used, except that tensile tests were conducted at multiple strain rates of 20%, 50%, and 100% L_0/min to investigate the rate-dependent mechanical behavior. For **2a**, **2c**, and **3a**, stress-strain curves were recorded at each strain rate to evaluate the tensile response and strain rate sensitivity.

Cyclic tensile testing: The same experimental conditions as described above were used, except that cyclic loading-unloading tests were performed at increasing strain amplitudes of 50%, 100%, 200%, 400%, and 750% for **2a**, and 50%, 100%, 200%, and 400% for **2c**. Each cycle consisted of loading to the target strain followed by unloading back to zero stress at a strain rate of 20% L_0/min. The specimens were allowed to recover completely between cycles. Stress-

strain hysteresis loops were recorded for each cycle to evaluate the energy dissipation behavior and mechanical resilience of the gel networks. The area enclosed by each hysteresis loop was calculated to quantify the dissipated energy at different strain levels. Fixed-strain cyclic tensile tests were also performed on **2a** and **2c** at a maximum strain of 400% for 7 consecutive cycles under the same testing conditions. For these fixed-strain cyclic tests, the hysteresis ratio was calculated as $(W_{loading} - W_{unloading})/W_{loading} \times 100\%$, where $W_{loading}$ and $W_{unloading}$ are the areas under the loading and unloading curves, respectively. The residual strain was determined as the strain at which the stress returned to zero during unloading, and the recovery efficiency was calculated as $(\varepsilon_{max} - \varepsilon_{residual})/\varepsilon_{max} \times 100\%$, where ε_{max} is the maximum applied strain. For time-dependent recovery tests, the same specimens were stored under vacuum for 1 day and then re-tested under identical cyclic conditions (**2a** at 750% strain and **2c** at 400% strain).

Computational details and definition of collective variables

Model construction

To isolate the intrinsic binding behavior of the pyrene core toward γ-cyclodextrin (γ-CD), a minimal molecular model, **1^{1}OH** (where n in 1^n denotes the number of EO group on each side), consisting of a pyrene core bearing two *p*-phenoxyethanol moieties on the 1 and 6 positions. This simplified structure retains the essential chemical features governing host–guest interactions, while minimizing unnecessary structural complexity. Building on prior reports of dt-inclusion complexes formed by pyrene dimers and γ-CD, the binding process was modeled using a dimeric **1^{1}OH** species. Accordingly, all binding free energy calculations were performed using a pre-associated **1^{1}OH** dimer as the guest species.

Free energy calculations: Free energy surfaces were calculated using the adaptive biasing force (ABF) method with geometric restraints to enhance sampling of the binding process, as implemented in PySAGES[4].

Definition of collective variables and geometric restraints

Primary binding: The free energy profile for the primary binding event between a **1^{1}OH** dimer and a single γ-CD was computed as a function of the axial projection distance between the center of mass of the **1^{1}OH** dimer core, defined as the pyrene and extended phenolic groups, and the center of the narrow rim of γ-CD. As illustrated in **Figure 2a**, this coordinate was obtained by projecting the corresponding vector along the principal symmetry axis of the γ-CD ring. The axial projection distance was calculated as $z_{proj} = (\vec{r}_{dimer} - \vec{r}_{CD,rim}) \cdot \vec{u}_{CD,axis}$, where

$\vec{r}_{dimer}$ is the center of mass of the **1¹OH** dimer core, $\vec{r}_{CD,rim}$ is the center of the γ-CD narrow rim, and $\vec{u}_{CD,axis}$ is the unit vector along the central axis of γ-CD.

To facilitate the sampling, we applied three types of geometric restraints:

1. RMSD restraint: a biasing potential was applied to all atoms in γ-CD:

$$U_{RMSD} = \frac{1}{2}k_{RMSD}\left(\xi_{RMSD} - \xi_{RMSD,0}\right)^2, \qquad if\ \ \xi_{RMSD} > \xi_{RMSD,0}$$

where ξ_{RMSD} is the root mean squared displacement (RMSD) value of γ-CD atoms with respect to its crystal structure, $\xi_{RMSD,0} = 1.5\ nm$.

2. General funnel shape restraint: a biasing potential was applied to atoms of the **1¹OH** core:

$$U_{Funnel} = \frac{1}{2}k_{Funnel}\left(\xi_{\perp} - f(\xi_{\parallel})\right)^2, \qquad if\ \xi_{\perp} > f(\xi_{\parallel})$$

where $\xi_{\perp}$ and $\xi_{\parallel}$ is the projection and the perpendicular projection of the center of mass of **1¹OH** core on the principal symmetry axis of the γ-CD, respectively. For simplicity, we chose $f(\xi_{\parallel}) = R_{cyl}$, with $R_{cyl} = 0.5\ nm$.

3. Alignment restraint: a biasing potential was applied to atoms of each **1¹OH** core:

$$U_{align} = \frac{1}{2}k_{align}\left(\xi_{\theta} - \xi_{\theta,0}\right)^2, \qquad if\ \xi_{\theta} < \xi_{\theta,0}$$

where $\xi_{\theta} = \cos^2(\theta)$, θ is the angle between the long axis of the **1¹OH** core and the principal symmetry axis of the γ-CD, with $\xi_{\theta,0} = 0.85$.

Secondary binding: To characterize the second binding step leading to the formation of the 2:2 complex, a two-dimensional free energy surface was constructed using two collective variables:

- **CV1** was defined as the axial separation between the center of mass of the second binding γ-CD and the center of the narrow rim of the first bound γ-CD.
- **CV2** was defined as the axial separation of the **1¹OH** dimer core (center of mass) relative to the center of the narrow rim of the first bound γ-CD.

Both distances were projected along the principal symmetrical axis of the first bound γ-CD ring (**Figure 2b**). These CVs were chosen to directly capture the relative positioning of the two γ-CD rings around the **1^{1}OH** dimer.

To facilitate the sampling, we applied three types of geometric restraints,

1. RMSD restraint: Same as the RMSD restraint in primary binding. This restraint was applied to each γ-CD ring.
2. General funnel shape restraint: Same as the general funnel shape restraint in primary binding. This restraint was applied to each **1^{1}OH** core as well as the second binding γ-CD.
3. Alignment restraint: Same as the alignment restraint in primary binding. This alignment restraint was applied to each **1^{1}OH** core as well as the second binding γ-CD. When it was applied to the second binding γ-CD, θ is the angle between the principal symmetry axis of two γ-CD rings, with the wide rim of both γ-CD rings facing each other.

NOESY calculation details: The atomistic simulation of the 2:2 complex (**1^{9}Acr$_2$:γ-CD$_2$**) was conducted with explicit TIP3P water, where **1^{9}Acr** indicates acrylate end-capped pyrene derivative containing nine ethylene glycol (EG) units and terminal acrylate groups on both ends of the phenyl pyrene core). The q4md-CD force field[5] was used to model the γ-CD molecules, and the generalized Amber force field (GAFF2)[6] was used to model the **1^{9}Acr** molecules. The simulated system contained a 2:2 complex and approximately 47000 water molecules in a simulation cell with approximate dimensions 11 × 11 × 11 nm^3. The system was first relaxed with the NPT ensemble (T = 298 K, P = 1 atm) for 1 ns, and switched to the NVT ensemble (T = 298 K) for 100 ns for the production run. The simulation was performed by OpenMM[7] with an integration timestep Δt = 1 fs. From the production run, we calculated $\langle r^{-6} \rangle$ as an estimate of the NOESY spectrum[8], where $\langle ... \rangle$ indicates the ensemble average, and r is the distance between protons.

Synthesis of acrylate end-capped pyrene thread (1^9Acr)

Synthesis of mono-tosylated nonaethylene glycol

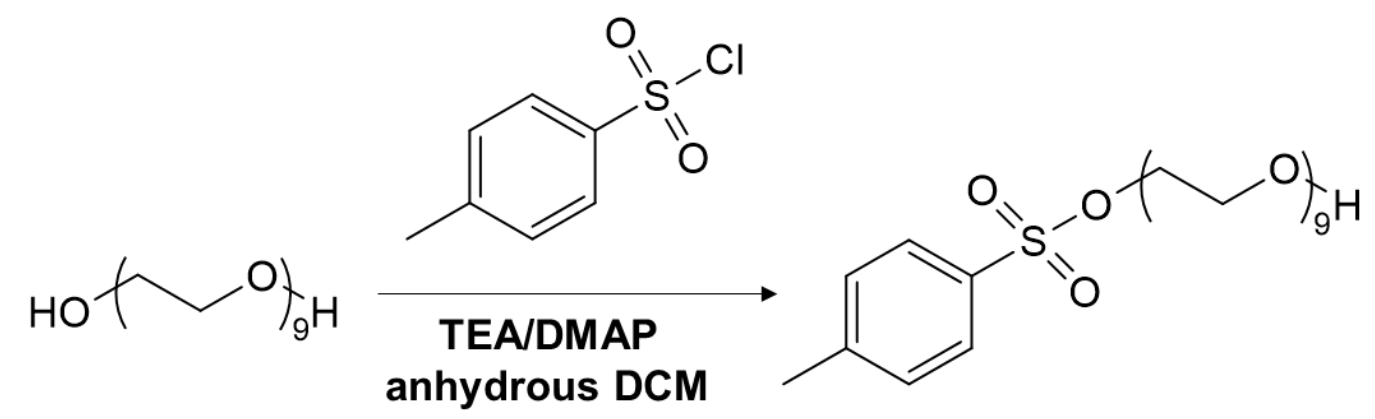

Figure S1**.** Synthesis of mono-tosylated nonaethylene glycol.

Nonaethylene glycol (12.5 g, 30.2 mmol), triethylamine (2.94 mL, 21.1 mmol), and 4-dimethylaminopyridine (0.123 g, 1.01 mmol) were dissolved in anhydrous dichloromethane (DCM) (110mL) in a flame-dried, two-neck round-bottom flask under argon. The solution was cooled to 0°C in an ice-water bath, and a solution of 4-toluenesulfonyl chloride (3.07 g, 16.1 mmol) in dry DCM (40 mL) was added dropwise over 16 h using a syringe pump. After the addition was complete, the mixture was allowed to warm to room temperature and stirred for an additional 8 h. The mixture was diluted with DCM (100 mL), washed with brine (1 × 250 mL), and the aqueous layer was extracted with DCM (3 × 150 mL). The combined organic phases were dried over sodium sulfate, filtered, and concentrated under vacuum. The crude product was purified by silica gel chromatography (0–10% methanol in ethyl acetate) to afford mono-tosylated nonaethylene glycol as a clear oil (5.87 g, 10.3 mmol, 64% isolated yield). ^{1}H NMR (500 MHz, $CDCl_3$): δ 7.80 (d, J = 8.1 Hz, 2H), 7.34 (d, J = 8.0 Hz, 2H), 4.16 (t, J = 4.8 Hz, 2H), 3.74 – 3.56 (m, 34H), 2.78 (t, J = 6.2 Hz, 1H), 2.45 (s, 3H). ^{13}C NMR (126 MHz, $CDCl_3$): 144.61, 133.09, 129.89, 128.03, 72.62, 70.80, 70.67, 70.62, 70.61, 70.59, 70.57, 70.39, 69.32, 68.73, 61.77, 21.70. MALDI-TOF MS ($[M + Na]^+$): found *m/z* 591.13 ($C_{25}H_{44}NaO_{12}S$ calcd. for *m/z* 591.25)

Synthesis of 1,6-di(4’-hydroxyphenyl)pyrene (**PyPh-OH**)[9]

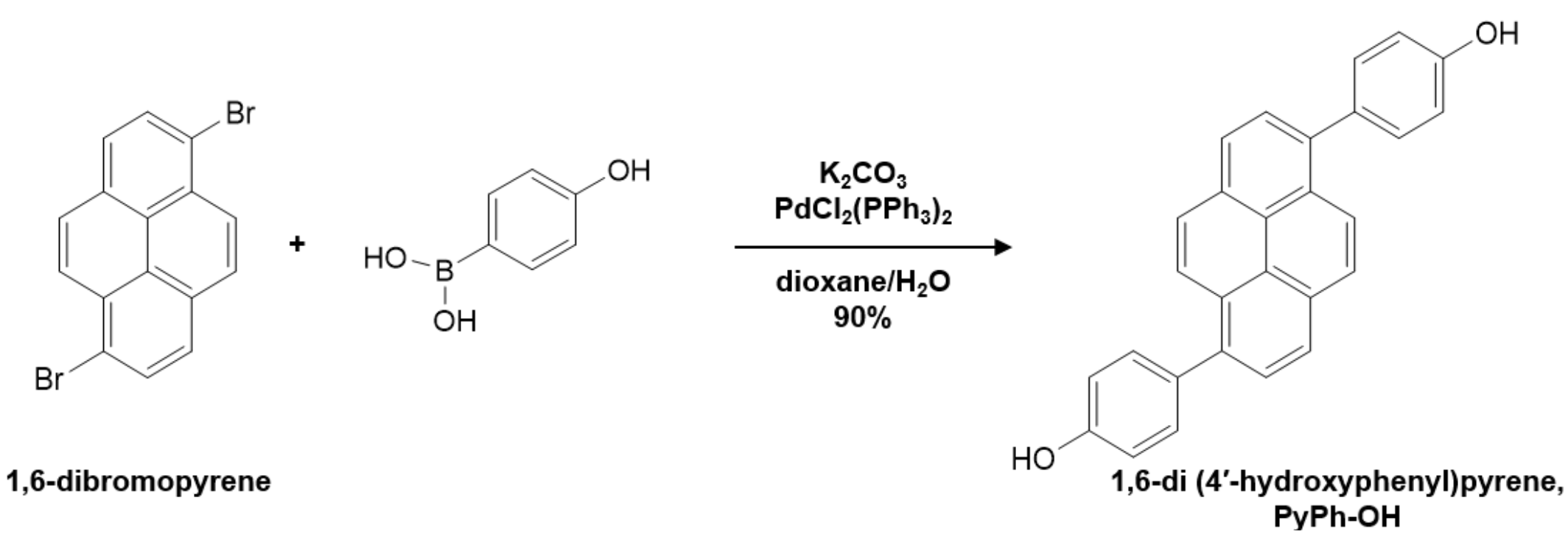

Figure S2. Synthesis of **PyPh-OH**

A mixture of 1,6-dibromopyrene (1.00 g, 2.78 mmol), 4-hydroxyphenylboronic acid (1.15 g, 8.45 mmol), K_2CO_3 (3.84 g, 27.8 mmol), and $PdCl_2(PPh_3)_2$ (234 mg, 0.334 mmol) in a mixture of 1,4-dioxane/H_2O (4:1, 55 mL) was refluxed under an argon atmosphere for 24 h. The reaction mixture was poured into water after cooling to room temperature and extracted with ethyl acetate (3 × 150 mL). The combined organic layers were dried over $MgSO_4$, filtered, and concentrated under reduced pressure. The resulting residue was purified by flash column chromatography on silica gel (ethyl acetate/hexanes) to afford **PyPh-OH** (964.40 mg, 2.50 mmol, 90% isolated yield). ^1H NMR (400 MHz, $CDCl_3$): δ 8.19 (m, 4H), 8.03 (d, J = 9.3 Hz, 2H), 7.96 (d, J = 7.8 Hz, 2H), 7.52 (m, 4H), 7.04 (d, J = 8.5 Hz, 4H). MALDI-TOF MS ($[M]^+$): found m/z 386.192 ($C_{28}H_{18}O_2$ calcd. for m/z 386.131).

Synthesis of photo-curable pyrene thread (**1⁹Acr**)

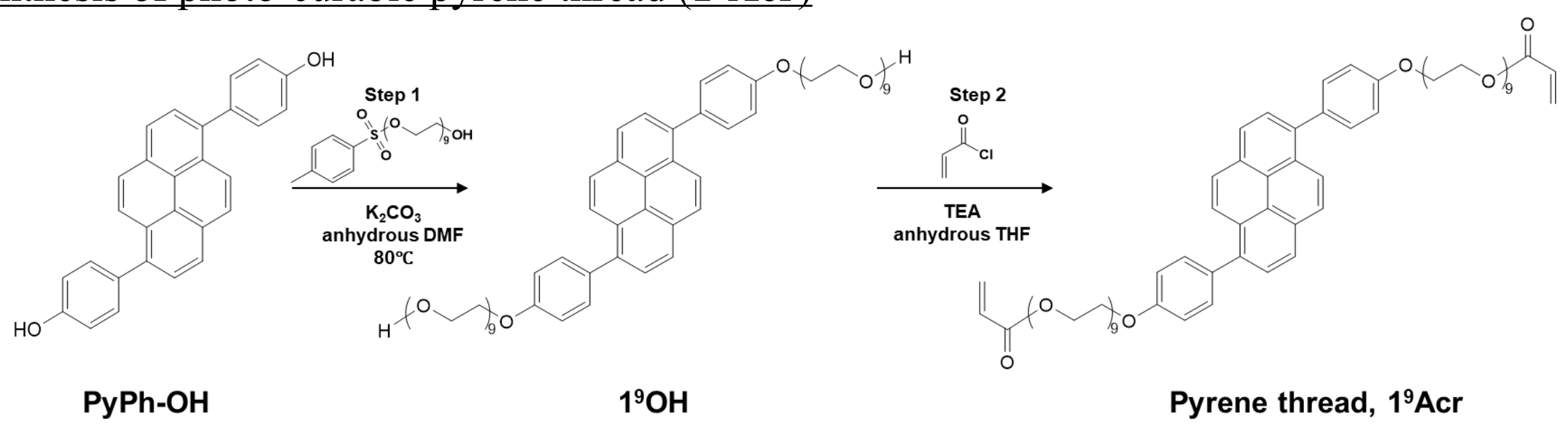

Figure S3. Synthesis of **1^9Acr**.

Step 1: A solution of mono-tosylated nonaethylene glycol (4.10 g, 7.21 mmol), **PyPh-OH** (930 mg, 2.40 mmol), and K_2CO_3 (797 mg, 5.77 mmol) in dry N,N-dimethylformamide (12 mL) was stirred at 75 °C for 24 h. The reaction mixture was diluted with chloroform, and the precipitate was removed by filtration. The filtrate was concentrated under reduced pressure and purified by silica gel chromatography (1% methanol in chloroform) to afford **1^9OH** (2.10 g, 1.78 mmol, 74% isolated yield). ^1H NMR (500 MHz, $CDCl_3$): δ 8.18 (d, J = 9.2 Hz, 2H), 8.17 (d, J = 7.9 Hz, 2H), 8.02 (d, J = 9.2 Hz, 2H), 7.95 (d, J = 7.9 Hz, 2H), 7.55 (d, J = 8.7 Hz, 4H), 7.12 (d, J = 8.4 Hz, 4H), 4.26 (t, J = 4.9 Hz, 4H), 3.94 (t, J = 4.9 Hz, 4H), 3.58–3.81 (m, 64H). ^{13}C NMR (126 MHz, $CDCl_3$): δ 158.36, 137.58, 133.98, 131.76, 130.36, 129.08, 127.95, 127.49, 125.42, 125.34, 124.52, 114.70, 72.68, 71.04, 70.86-70.60, 70.43, 69.96, 67.73, 61.85. MALDI-TOF MS ($[M + Na]^+$): found m/z 1201.606 ($C_{64}H_{90}O_{20}Na$ calcd. for m/z 1201.591).

Step 2: **1^9OH** (1.00 g, 0.85 mmol) and triethylamine (0.21 g, 2.13 mmol) were placed in a round-bottom flask purged with argon, and anhydrous THF (30 mL) was added. Acryloyl chloride (0.19 g, 2.13mmol) was then added dropwise to the flask, and the mixture was stirred for 6 hours. Afterward, THF was removed in vacuo. The resulting residue was dissolved in

ethyl acetate and extracted using water three times, followed by drying with $MgSO_4$. Insoluble materials were filtered off, and the filtrate was further purified by silica gel chromatography (0-2% methanol in chloroform) to afford **1^9Acr** (0.56 g, 0.44 mmol, isolated yield 52%). ^{1}H NMR (500 MHz, $CDCl_3$) δ 8.18 (dd, *J* = 8.6, 4.4 Hz, 4H), 8.02 (d, *J* = 9.3 Hz, 2H), 7.95 (d, *J* = 7.9 Hz, 2H), 7.55 (d, J = 8.7 Hz, 4H), 7.12 (d, J = 8.4 Hz, 4H), 6.42 (dd, *J* = 17.3, 1.6 Hz, 2H), 6.14 (dd, *J* = 17.5, 10.4 Hz, 2H), 5.82 (dd, *J* = 10.5, 1.6 Hz, 2H), 4.33–4.23 (m, 8H), 3.94 (t, *J* = 4.8 Hz, 4H), 3.79 (dd, *J* = 6.0, 3.5 Hz, 4H), 3.75–3.59 (m, 56H). ^{13}C NMR (126 MHz, DMSO) δ 165.63, 158.20, 137.23, 132.80, 131.84, 131.63, 129.86, 128.37, 128.27, 128.11, 127.74, 124.90, 124.73, 114.86, 114.83, 72.50, 70.16, 70.02, 69.98, 69.97, 69.95, 69.93, 69.92, 69.87, 69.17, 68.37, 67.48, 63.62, 60.36. MALDI-TOF MS ($[M+H]^+$): found m/z 1286.516 ($C_{70}H_{94}O_{22}$ calcd. for m/z 1286.624).

Host-guest complexation experiments

Fluorescence titration of γ-cyclodextrin into **1^9Acr** in aqueous solution

An aqueous solution of **1^9Acr** was prepared at a concentration of 0.2 mM (1.0 mL, 0.2 μmol) and was titrated by stepwise addition of a concentrated γ-CD stock solution (2 mM) containing 0.2 mM **1^9Acr**, thereby maintaining the **1^9Acr** concentration constant throughout the experiment. After each addition, the mixture was gently stirred and analyzed by fluorescence spectroscopy to monitor host–guest complex formation. The titration was carried out until a final **γ-CD/1^9Acr** molar ratio of 8 was reached.

NMR titration of γ-cyclodextrin into **1^9Acr** in aqueous solution

An aqueous solution of **1^9Acr** was prepared at a constant concentration of 2 mM and titrated by stepwise addition of a concentrated γ-CD stock solution containing 2 mM **1^9Acr**, thereby maintaining the **1^9Acr** concentration constant throughout the experiment. After each addition, the mixture was gently stirred and analyzed by ^{1}H NMR spectroscopy in D_2O to monitor host–guest complex formation. The titration was carried out until a final **γ-CD/1^9Acr** molar ratio of 8 was reached.

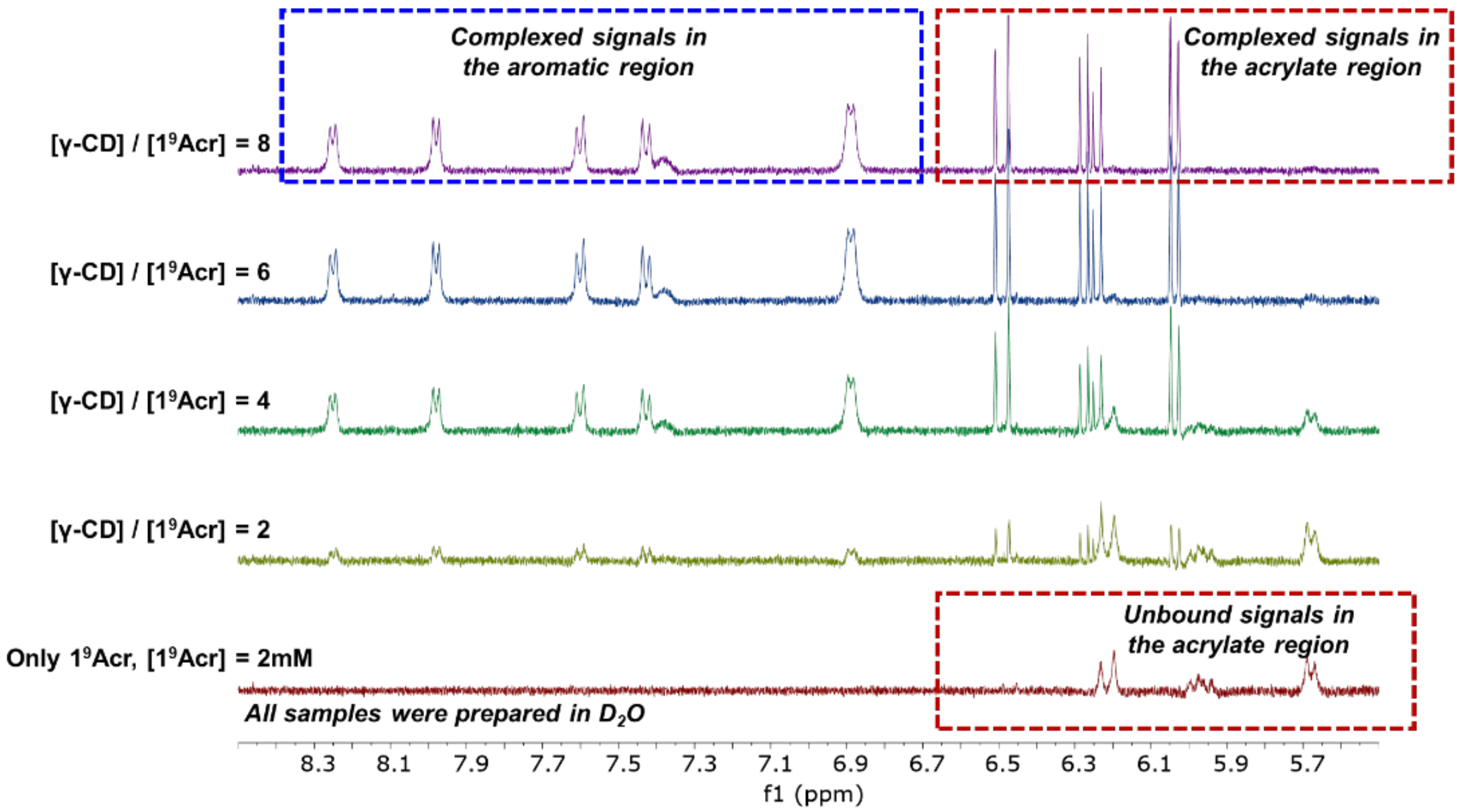

Figure S4. ^{1}H NMR spectra (500 MHz, 25 °C, D_2O) of **1^9Acr** (2 mM) with increasing γ-CD equivalents (0–8 equivalents, bottom to top), showing gradual disappearance of unbound acrylate signals and emergence of complexed signals.

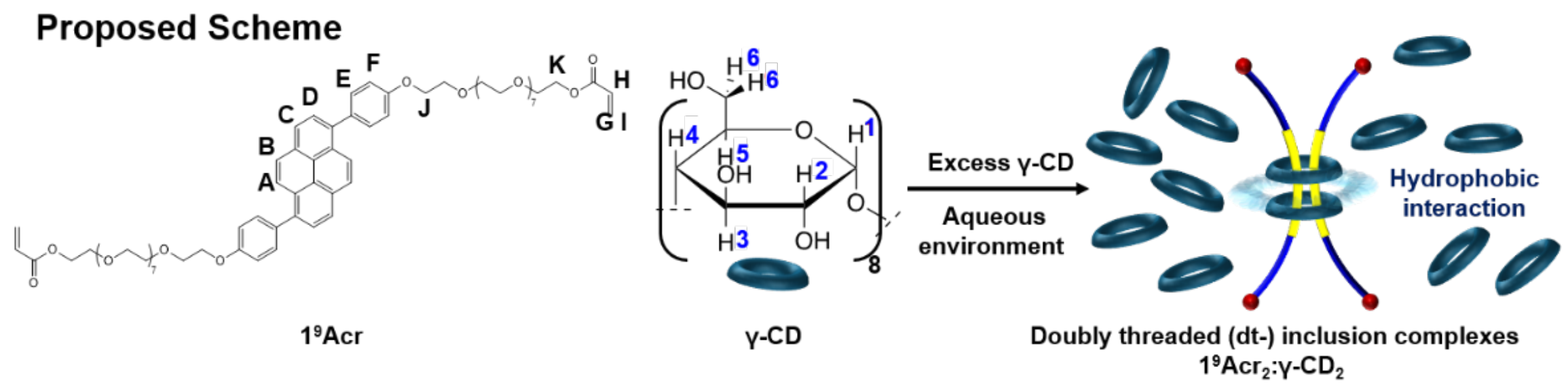

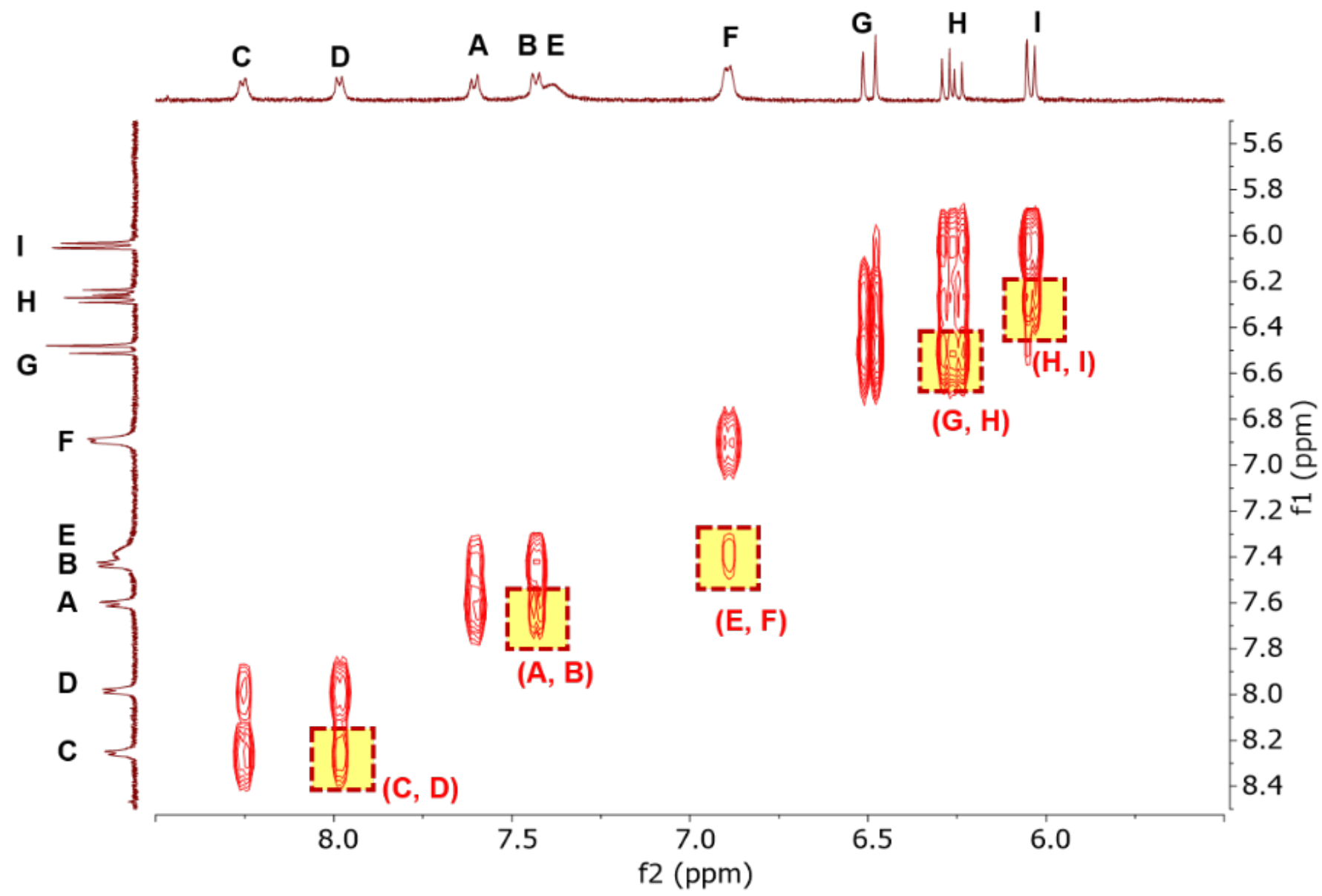

Figure S5. COSY NMR spectrum of the **1^9Acr**/γ-CD complex in D_2O (500 MHz, 25 °C) for the assignment of aromatic and acrylate-region protons (A–I) of **1^9Acr**.

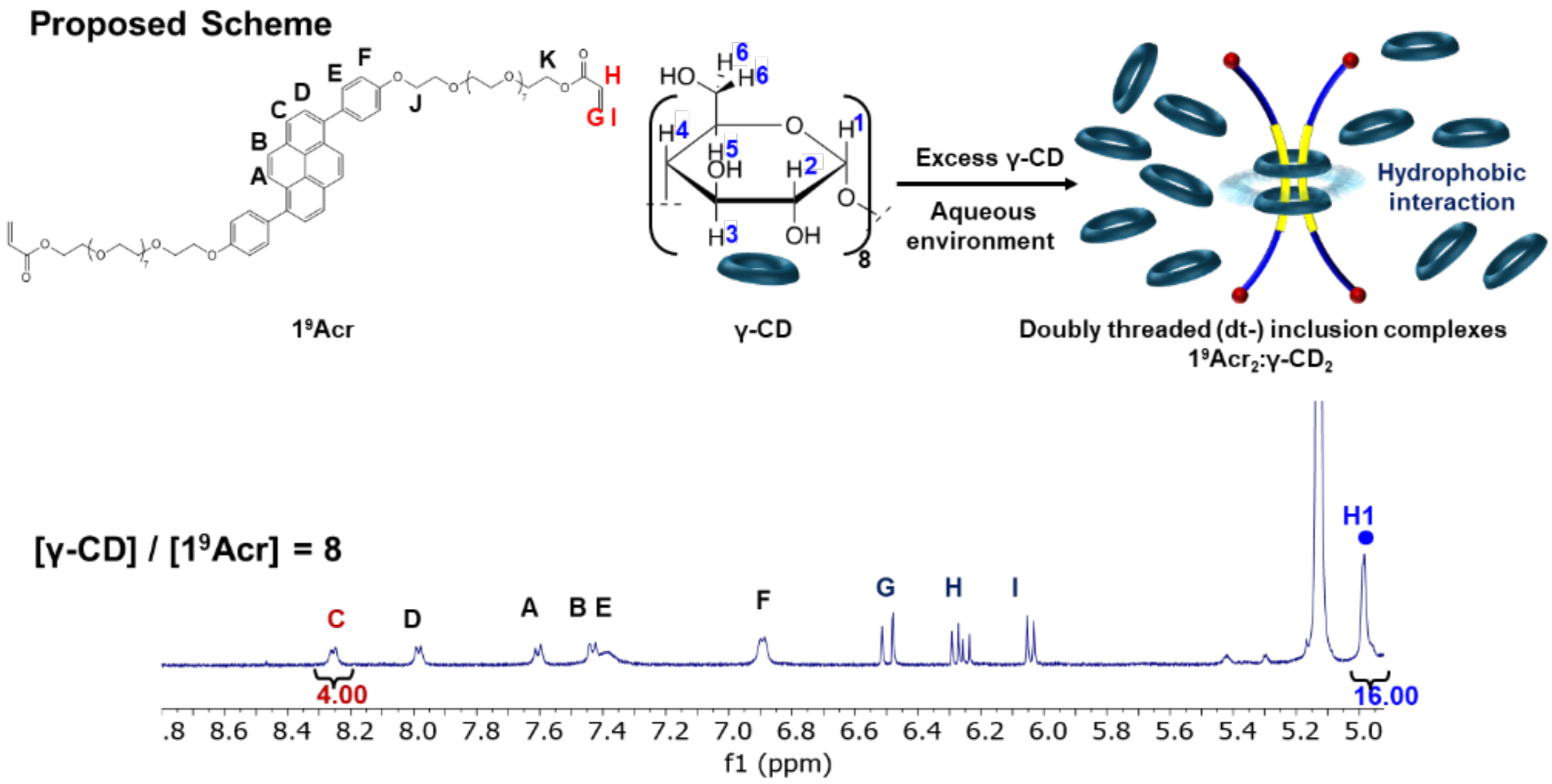

Figure S6. ^{1}H NMR spectrum of the inclusion complex between **1^9Acr** and γ-CD in D_2O (500 MHz, 25 °C). The integration ratio between **1^9Acr** (H_C, 4.00H) and γ-CD (, 16.00H) confirms an equimolar (n:n) complexation stoichiometry. The schematic above illustrates the proposed doubly threaded 2:2 assembly consistent with this stoichiometry.

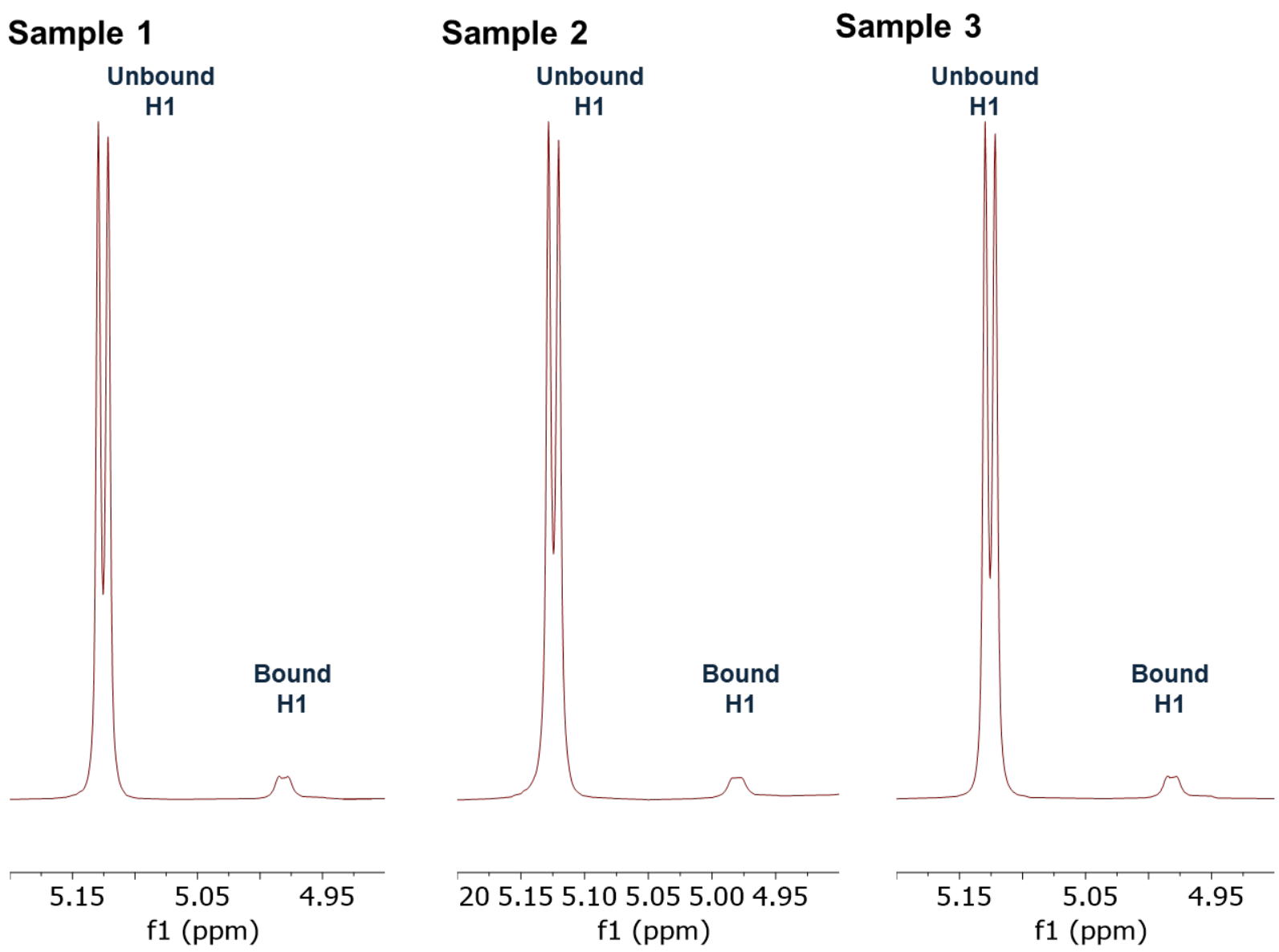

Figure S7. Partial ^{1}H NMR spectra highlighting the H_1 proton signals of γ-cyclodextrin in three independent samples. Both unbound and bound γ-CD signals are observed, and the ratio of bound to unbound γ-CD, determined from the average H_1 integrals, was approximately 1:7.

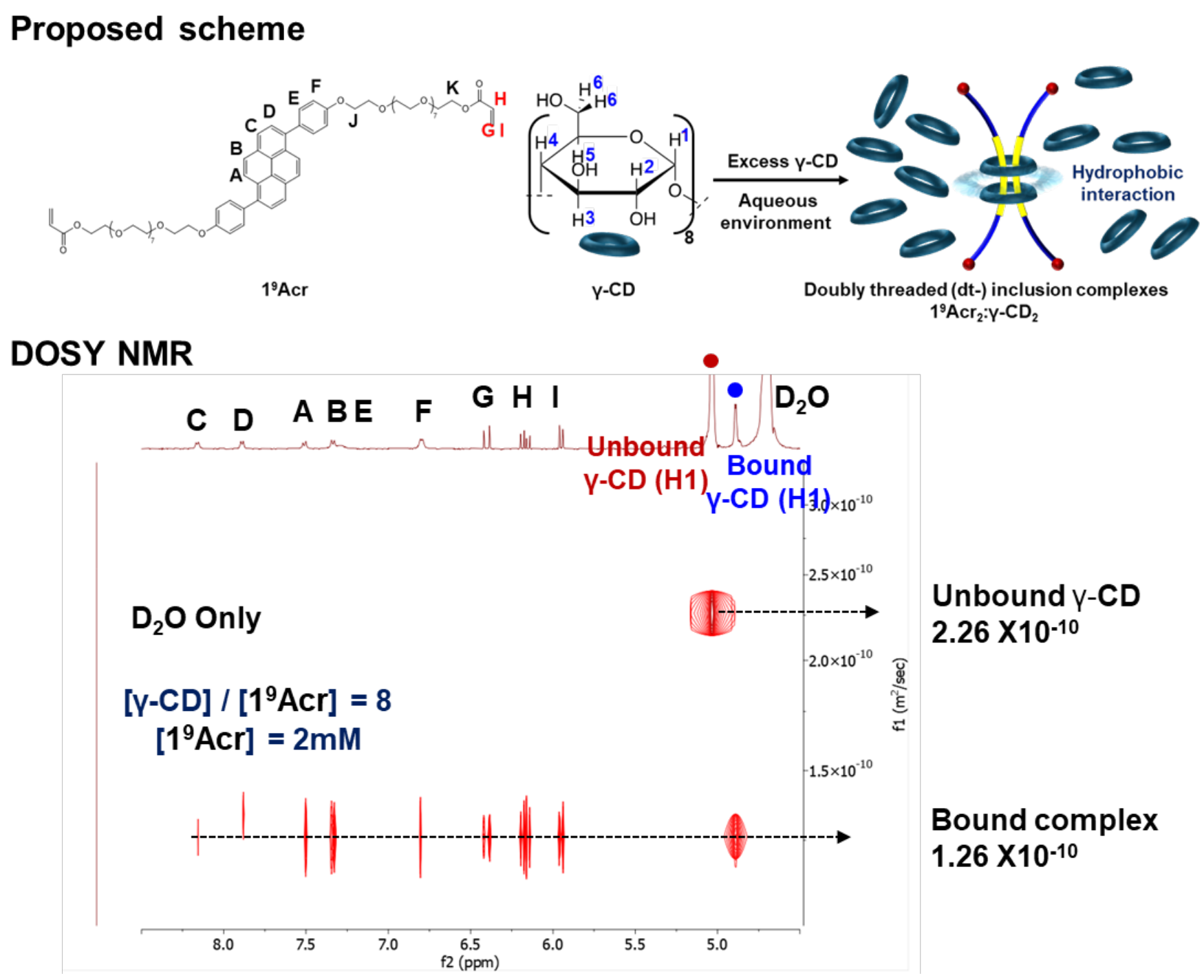

Figure S8. DOSY ^{1}H NMR spectra recorded under conditions of [γ-CD] / [**1^9Acr**] = 8, [**1^9Acr**] = 2 mM. The diffusion coefficients of the bound γ-CD (H1) and the aromatic protons of **1^9Acr** ($H_{A\text{-}I}$) are nearly identical ($D \approx 1.26 \times 10^{-10}$ m^2/s), confirming complex formation. In contrast, unbound γ-CD displays a substantially higher diffusion coefficient ($D \approx 2.26 \times 10^{-10}$ m^2/s).

<u>Comparison of NOESY spectrum between free **1^9Acr** and **1^9Acr/γ-CD** complex</u>

To further investigate the complexation behavior, a control solution of **1^9Acr** in DMSO-d_6, which represents the unbound state, was prepared at 2 mM concentration and its NOESY spectrum was compared with that of the **1^9Acr/γ-CD** complex.

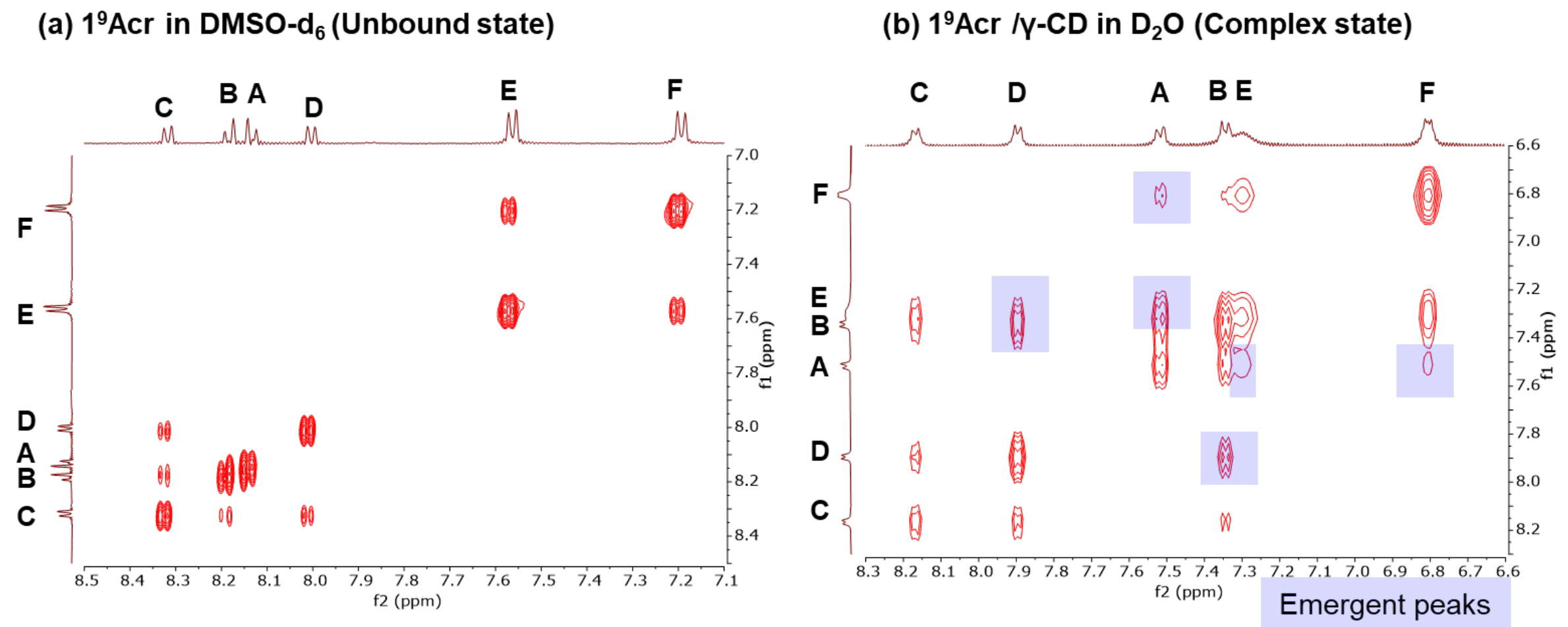

Figure S9. NOESY spectral comparison of (a) unbound **1^9Acr** and (b) **1^9Acr**/γ-CD complex in the aromatic region. Upon addition of γ-CD to **1^9Acr** in aqueous solution, **1^9Acr** dimers were bound within γ-CD, demonstrating emergent intermolecular cross-peaks (highlighted in blue).

Simulated NOESY spectra for free **1^{9}Acr** and **1^{9}Acr$_2$:γ-CD$_2$** complex

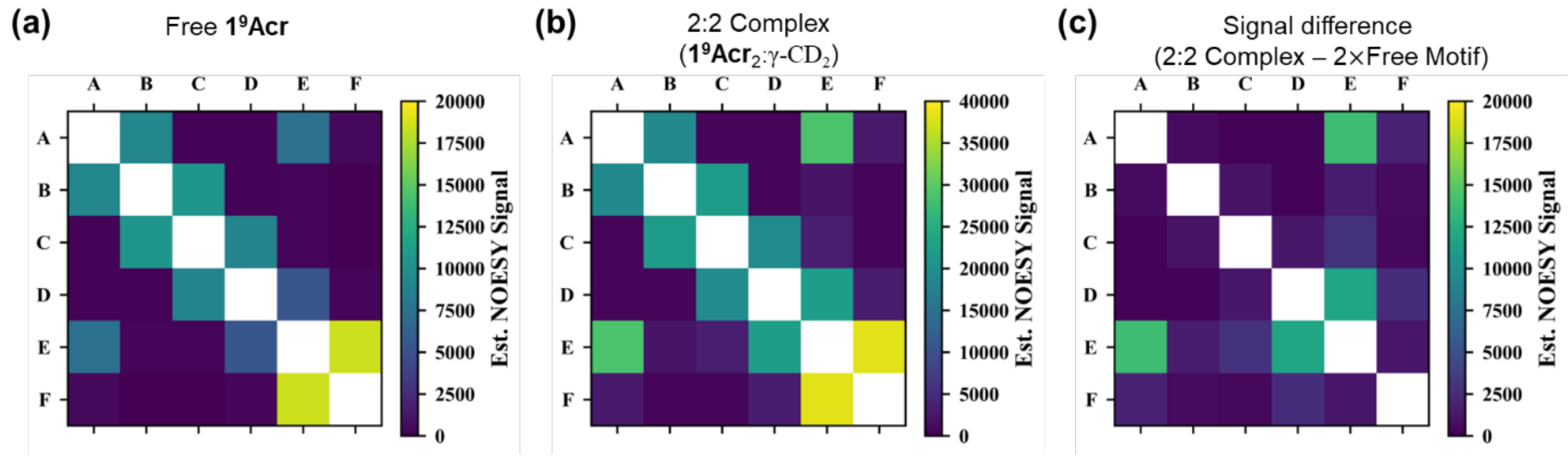

Figure S10. Estimated NOESY signal from aromatic protons of the **1^{9}Acr** motif for (a) a free **1^{9}Acr**, and (b) an inclusion complex (**1^{9}Acr$_2$**:γ-CD$_2$). Panel (c) shows the difference in NOESY signals between the 2:2 complex and two free motifs, revealing that complexation brings the A–E and D–E protons into closer spatial proximity. This observation suggests the formation of π–π stacking between the pyrene cores in the complex.

Fluorescence titration of pregel with varying HEA/water ratios

To investigate the effect of HEA content on the threading assembly, fluorescence spectroscopy was used to monitor solutions prepared with different HEA/water ratios while maintaining constant concentrations of **1^{9}Acr** and γ-CD. For this purpose, aqueous and HEA-containing stock solutions were prepared such that both **1^{9}Acr** and γ-CD were present at the same concentrations and at a fixed molar ratio of 1:8. By mixing these two solutions in different proportions, the HEA/water ratio was systematically varied without altering the overall concentrations of **1^{9}Acr** or γ-CD. Fluorescence emission spectra were recorded at room temperature using an excitation wavelength of 375 nm. As shown in **Figure 5c**, at low HEA content (HEA/water = 11/89 v/v), the emission maximum was centered at ca. 450 nm, consistent with a predominantly doubly threaded state. As the HEA fraction increased, the emission progressively shifted toward shorter wavelength with increasing monomer-like character around ca. 420 nm, indicating disruption of the doubly threaded assembly and the formation of less-associated or singly threaded species.

Preparation of 1^{9}Acr/γ-CD in DMSO-d$_6$/D$_2$O mixtures for NMR analysis

Fluorescence and NMR spectra of **1^{9}Acr/γ-CD** in DMSO-d$_6$/D$_2$O mixtures

To examine the effect of solvent composition on the threading topology by NMR spectroscopy, DMSO-d$_6$/D$_2$O mixtures were prepared with volume ratios matched to those used in the HEA/water pregel formulations, replacing HEA/water with DMSO-d$_6$/D$_2$O to avoid spectral overlap. A stock solution of 1^{9}Acr and γ-CD in DMSO-d$_6$ was first prepared at a [γ-CD]/[1^{9}Acr] ratio of 8. This stock solution was divided into three aliquots, and different

volumes of D_2O were added to each aliquot to obtain the final DMSO-d_6/D_2O volume ratios of 11/89, 19/81, and 30/70 v/v for samples A–C, respectively. The final samples had [1^9Acr] = 0.50 mM, 0.91 mM, and 1.41 mM for samples A–C, respectively, calculated from the total DMSO-d_6/D_2O volume, while maintaining a constant [γ-CD]/[1^9Acr] ratio of 8. The fluorescence emission spectra of these samples were first recorded to confirm that the DMSO-d_6/D_2O mixtures reproduce the same solvent-dependent trends observed in the corresponding HEA/water pregel systems (**Figure S11**). ^{1}H NMR spectra were recorded at 500 MHz and 25 °C (**Figure S12**), and chemical shifts were referenced to residual solvent peaks.

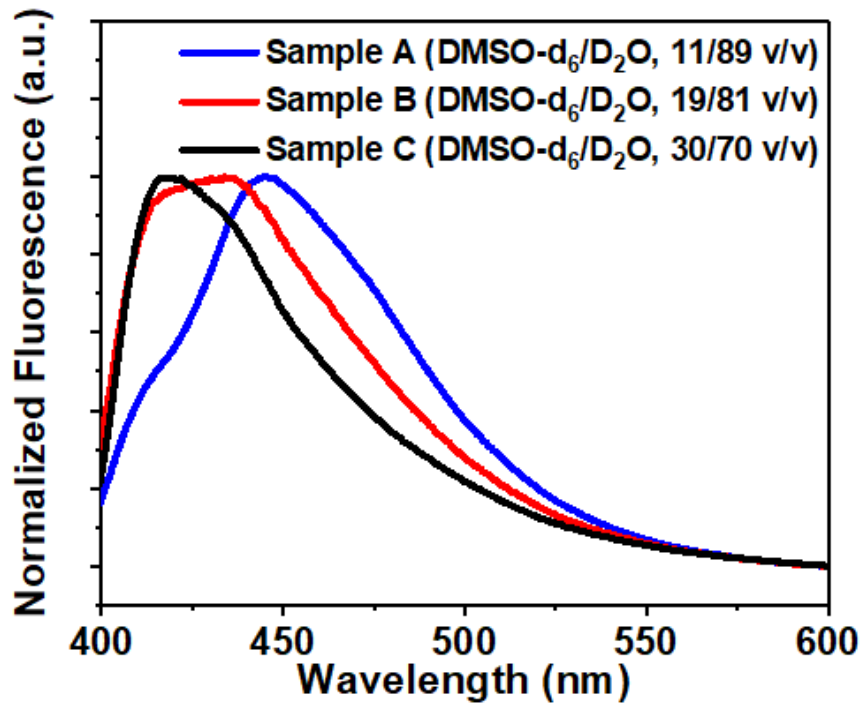

Figure S11. Normalized fluorescence spectra of **1^9Acr**/γ-CD complexes in DMSO-d_6/D_2O mixtures prepared using the same solvent volume ratios as those employed in the HEA/water pre-gel formulations.

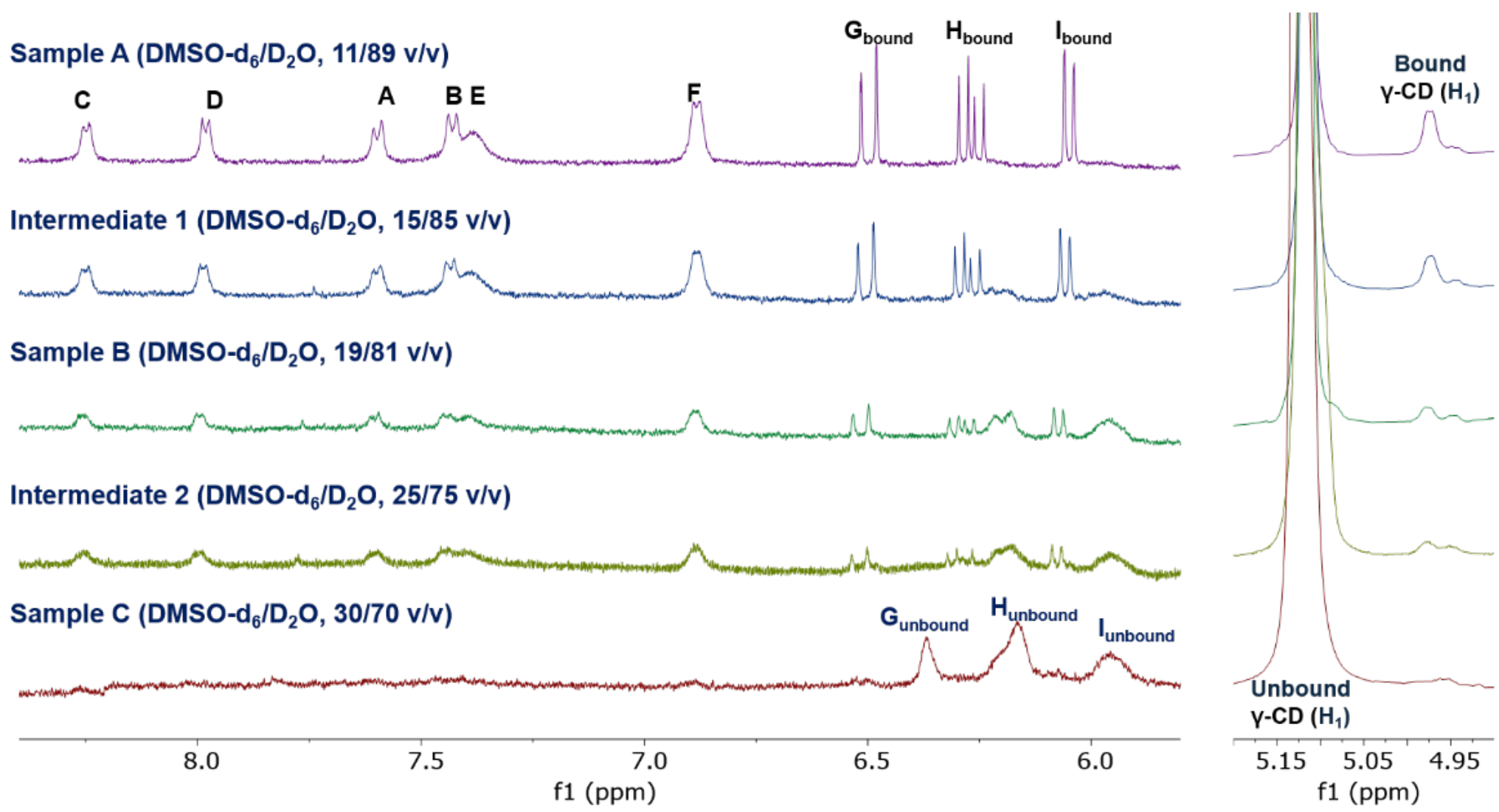

Figure S12. ^{1}H NMR spectra of **1^9Acr**/γ-CD complexes in DMSO-d_6/D_2O mixtures with varying solvent compositions. As the DMSO-d_6 ratio increases, the acrylate signals progressively broaden and shift from the bound chemical shifts, consistent with the gradual decomplexation of the doubly threaded species.

NOESY measurements of **1^{9}Acr/γ-CD** in DMSO-d_6/D_2O mixtures

2D NOESY experiments were carried out on Sample C (DMSO-d_6/D_2O = 30/70 v/v) to probe spatial proximities between γ-CD and pyrene protons. Spectra were recorded at 25, 15, and 5 °C with a mixing time of 600 ms (**Figure S13**). A control NOESY spectrum was also recorded for the **1^{9}Acr/γ-CD** system in neat DMSO-d_6 under otherwise identical conditions (**Figure S14**).

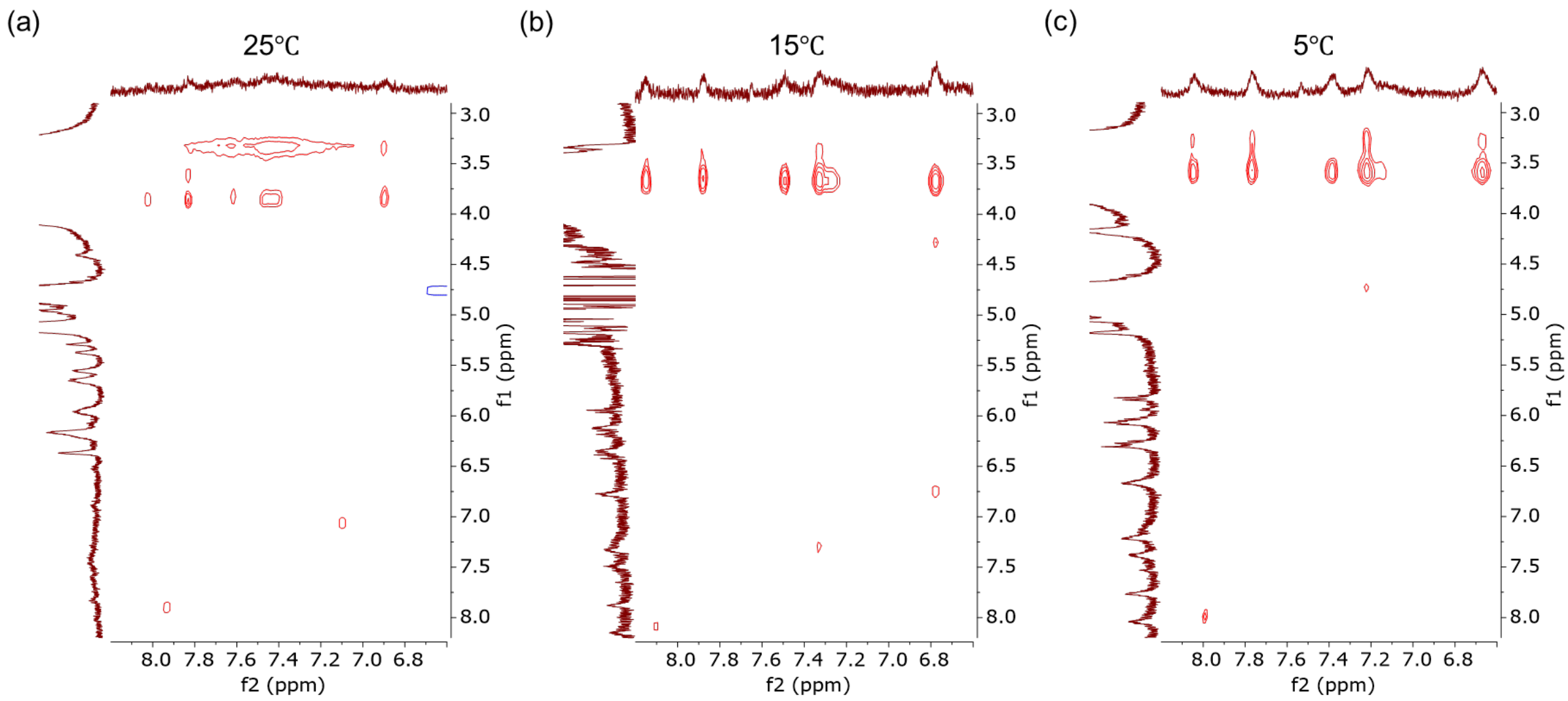

Figure S13. Temperature-dependent 2D NOESY spectra of **1^{9}Acr**/γ-CD in DMSO-d_6/D_2O (30/70 v/v; [**1^{9}Acr**] = 1.41mM, [γ-CD] / [**1^{9}Acr**] = 8), recorded at (a) 25, (b) 15, and (c) 5 °C.

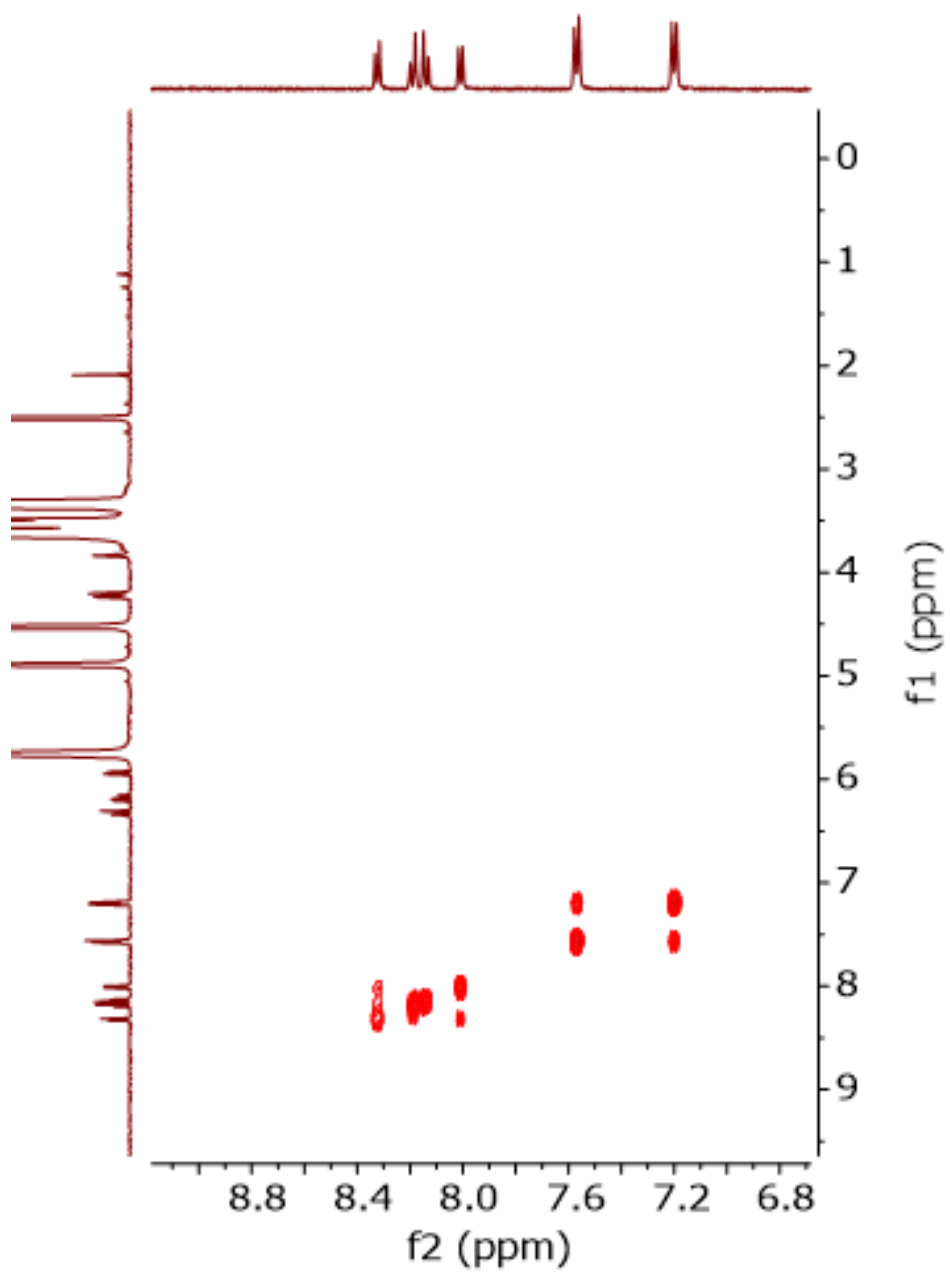

Figure S14. 2D NOESY spectra of **1^{9}Acr**/γ-CD in neat DMSO-d_6, recorded at 25 °C ([**1^{9}Acr**] = 1.41mM, [γ-CD] / [**1^{9}Acr**] = 8).

Preparation of representative pregel formulations (pregel A-C)

Representative pregel formulations were prepared to examine the spectroscopic characteristics of mixtures with distinct HEA/water ratios. In each case, the amounts of **1⁹Acr** (0.3 μmol, 1equiv.), γ-CD (2.4 μmol, 8 equiv.), and 2-hydroxyethyl acrylate (HEA, 0.6 mmol, 2000 equiv.) were kept constant, while only the water content was varied to adjust the HEA/water volume ratio. The molar ratio of **1⁹Acr** to γ-CD was fixed at 1:8 throughout. The resulting samples are denoted as pregel A (HEA/water = 11/89 v/v), pregel B (19/81 v/v), and pregel C (30/70 v/v) (Table S1). Calculated on the basis of the total volume of HEA and water, the corresponding concentrations of **1⁹Acr** were 0.50 mM, 0.91 mM, and 1.41 mM for pregel A, B, and C, respectively. Emission spectra were recorded at room temperature using an excitation wavelength of 375 nm, and the corresponding spectra are shown in Figure 5d.

	**Pregel A**	**Pregel B**	**Pregel C**
**1⁹Acr**, MW 1287.50 g/mol	0.3 μmol	0.3 μmol	0.3 μmol
γ-CD, MW 3145 g/mol	2.4 μmol	2.4 μmol	2.4 μmol
2-hydroxy ethyl acrylate (HEA), MW116.12 g/mol	0.6 mmol (63 μL)	0.6 mmol (63 μL)	0.6 mmol (63 μL)
water	534 μL	267 μL	150 μL
HEA/water (v/v)	11:89	19:81	30:70

Table S1. Composition of pregel solutions.

Fabrication of topology engineered networks

Three pregel solutions were prepared with **1⁹Acr**, γ-CD, water, and liquid 2-hydroxyethyl acrylate (HEA) resin containing 0.5 wt% of a water-soluble photo-initiator (lithium phenyl-2,4,6-trimethylbenzoylphosphinate, LAP) following the composition as depicted in Table S1. Furthermore, an additional pregel solution was prepared for a pyrene containing non-interlocked control covalent gel without γ-CD (**3a**). This control solution comprised 0.3 μmol of **1⁹Acr**, 0.6 mmol of HEA, and 150 μL of DMSO in place of water, which prevents pyrene aggregation in aqueous media and enables the gel to possess similar topology to **2c** from pregel C. The pregel solutions were dropped onto a 1H,1H,2H,2H-perfluoro octyl trichlorosilane (PFOCTS) coated glass slides[10] with a spacer thickness of 600 μm, and they were covered with another PFOCTS-coated glass slide. Subsequently, these solutions were irradiated with long-wavelength ultraviolet light (Wavelength: 390–500 nm,

Intensity: 200 mW/cm^2) using a filtered light source (Bluepoint 4 Ecocure from Honle UV America) for a total of 120minutes, consisting of 60minutes of upward irradiation followed by 60minutes of downward irradiation. The UV irradiation of the pregel solution initiated free-radical acrylate polymerization, generating distinct crosslinked networks (**2a** derived from doubly threaded pregel A, **2b** derived from mixed threaded pregel B, **2c** derived from singly threaded pregel C, and **3a**). The weight of each dried gel network was recorded after drying the gel under vacuum for 24 hours.

To remove the uncrosslinked fraction, sequential washing with methanol followed by water was conducted, with three washing cycles performed for each solvent. The gels were first immersed in large beakers containing 100 mL of methanol at 60 °C on hot plates. A cover glass was placed on top of the beaker to prevent solvent evaporation, and the gels were subjected to iterative washing with fresh 100 mL of methanol every 10 hours for a total duration of 30 hours (3 washing cycles). The gels were subsequently washed with fresh 100 mL of water under the same conditions for an additional three washing cycles. The extracted fractions were utilized for quantification of γ-CD content in subsequent analyses. After drying the gels under vacuum for 24 hours, the final gel networks **2a**, **2b**, **2c,** and **3a** were obtained. To evaluate possible HEA-derived background network formation, control formulations containing HEA at the same HEA/water ratios as those used for **2a**–**2c** were photopolymerized under identical conditions in the absence of γ-CD and **1^{9}Acr**. After the same methanol/water washing procedure, no insoluble gel fraction was obtained, indicating that HEA-derived self-crosslinking or chain-transfer-induced branching alone is insufficient to generate a stable gel under the present conditions.

<u>Gel fraction of topology engineered networks</u>

The gel fraction (GF) was calculated using the following Equation:

$$\text{GF (wt\%)} = \frac{W_{dry,after\ methanol/water\ washing}}{W_{dry,crude}} \times 100$$

Where $\boldsymbol{W_{dry,\ after\ methanol/water\ washing}}$ represents the weight of the dried gel after methanol/water washing, and $\boldsymbol{W_{dry,\ crude}}$ represents the weight of the dried gel after photocrosslinking.

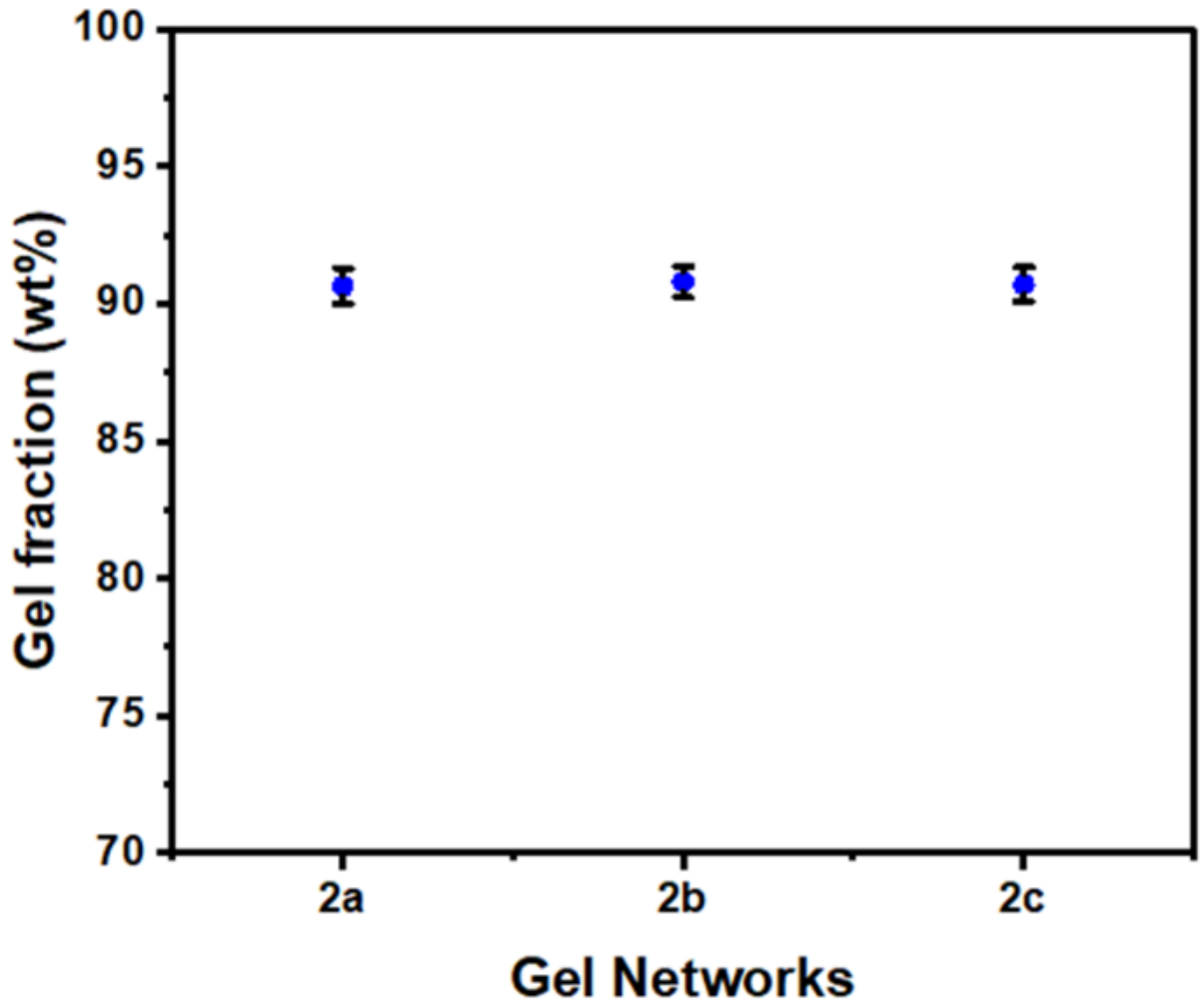

Figure S15. Gel fraction of the gel networks with distinct topology. Monomer compositions for gel synthesis are listed in Table S1.

Characterization of these gel networks

Quantification of γ-CD content in topological gel networks

To characterize the topology of these fabricated gel networks (**2a**-**2c**), it is crucial to determine the amount of γ-CD remaining within the gel networks after methanol/water washing (soluble fraction). The incorporated γ-CD content was determined using external standard NMR methodology, by comparing the molar ratio of a reference molecule (thymol) to γ-CD in the soluble fractions from each gel network. To quantify the mole number of γ-CD in each soluble fraction, a control solution was prepared using the identical mixture compositions as the original pregel solutions of **1^9Acr** (0.3 µmol) and γ-CD (2.4 µmol, 8 equivalents relative to **1^9Acr**). Since all pregel solutions contained identical γ-CD amounts, comparing the integral ratios of γ-CD in the soluble fraction to those in the control solution enabled quantitative determination of the remaining γ-CD amount. This approach accounts for the selective removal of uncomplexed γ-CDs during the washing process, providing insight into the threading topology in each gel network. Each soluble fraction was dried under vacuum and subsequently re-dissolved in 550 µL of DMSO-d_6 before transferring to NMR tubes. For external standard sample, the thymol (NMR *Trace*CERT®, 0.0060 g) was also dissolved in 100 µL of DMSO-d_6 and then transferred to Wilmad® coaxial insert tube. The same coaxial insert tube was added to each NMR tube containing either the control solution or the soluble fractions to ensure consistent quantification conditions across all measurements.

The number of γ-CD molecules in each soluble fraction was quantified via ^{1}H NMR spectroscopy using external standard thymol as the diagnostic reference. In the control solution, the integral of the proton H_1 peak of γ-CD (4.89 ppm, 8H) was compared to the aromatic proton H_A of thymol (6.95 ppm, 1H). When the integral of the H_A peak was normalized to 100.00, the γ-CD signal corresponded to an integral of 48.02 in the pregel control solution. In the soluble fractions obtained after methanol/water washing of the gels, the γ-CD integral was consistently measured at approximately 42.00 relative to the same thymol reference. Comparing the γ-CD/thymol integral ratios between the control solution and the post-washing soluble fractions indicates that approximately 7 equivalents (2.1 µmol) of the initial 8 equivalents (2.4 µmol) of γ-CD were removed during washing. This analysis further demonstrates that the majority of unbound γ-CD was efficiently removed during washing, whereas γ-CD species incorporated through host–guest complexation were preferentially retained within the gel network, independent of the threading stoichiometry. The retained γ-CD content therefore corresponds to complexed species incorporated into network rather than physically trapped free γ-CD.

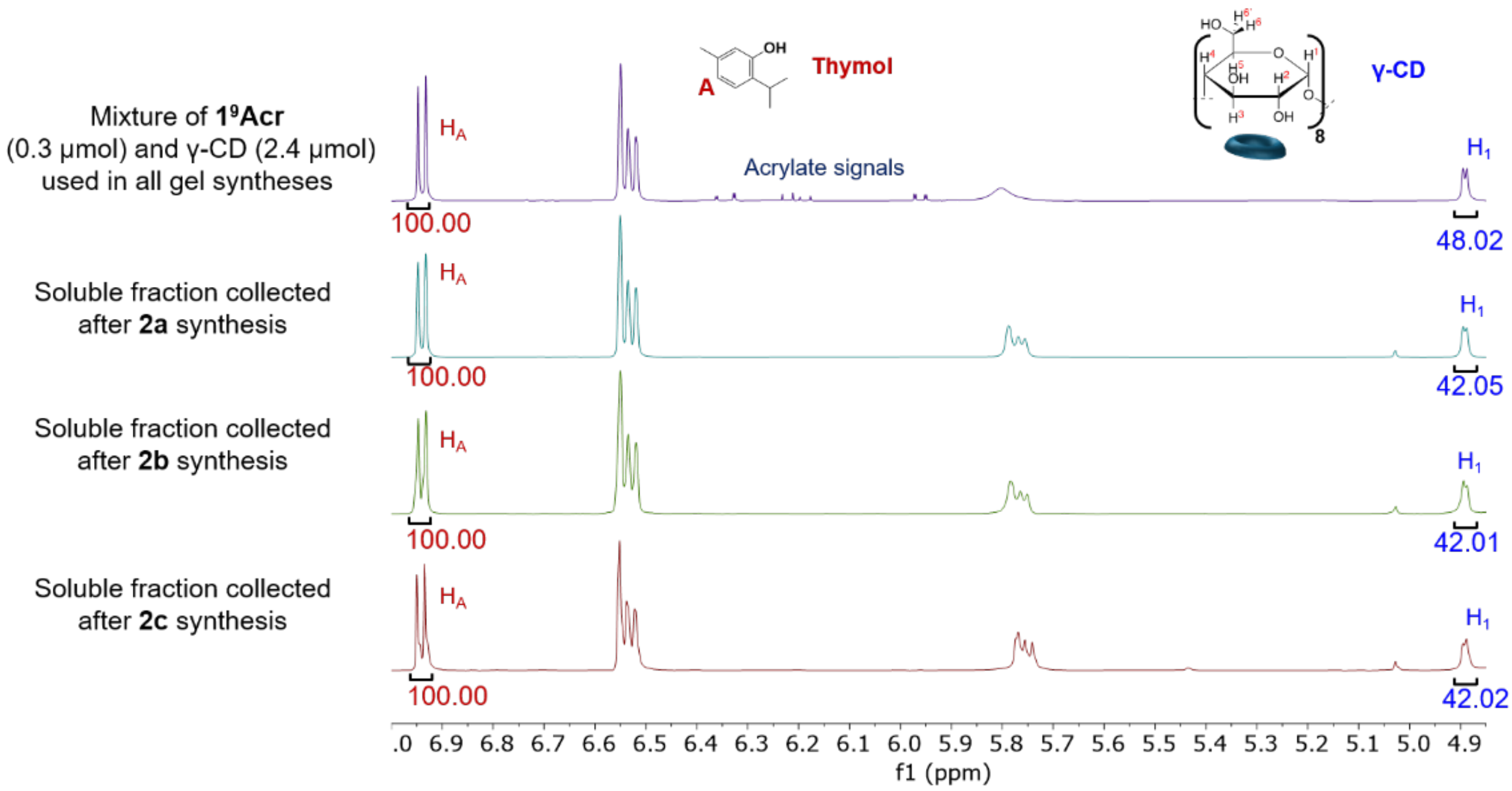

Figure S16. Quantification of γ-CD content in soluble fractions by external standard ^{1}H NMR spectroscopy. Representative NMR spectra of a control solution (top) and soluble fractions collected after **2a**, **2b**, and **2c** synthesis and washing show the diagnostic thymol H_A proton (6.95 ppm) and the anomeric H_1 proton of γ-CD (4.89 ppm), with integrals normalized to the thymol signal (100.00).

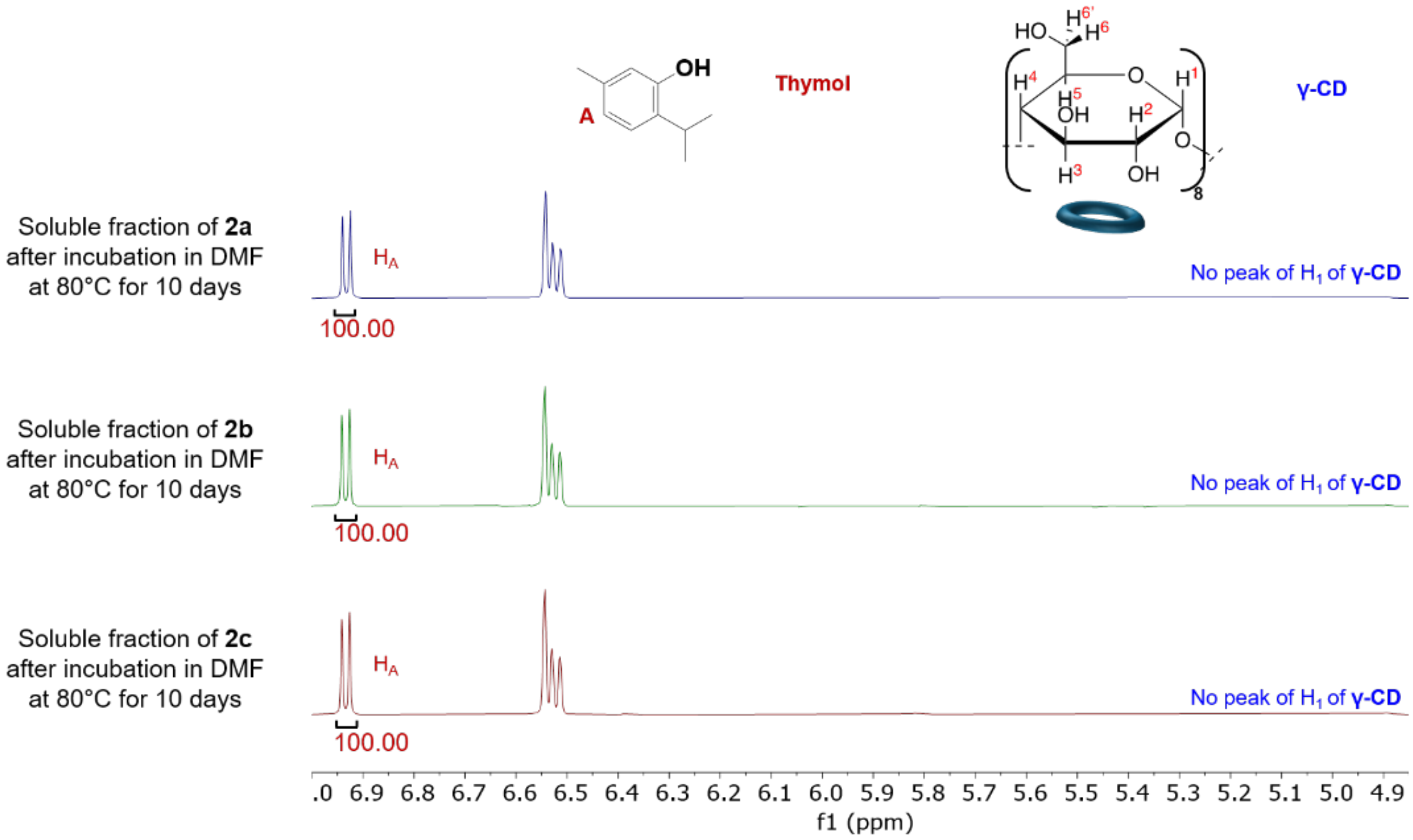

Figure S17. ^{1}H NMR spectra of soluble fractions collected after incubation in DMF at 80°C for 10 days, analyzed using thymol as an external reference (H_A, 6.95 ppm). No detectable proton of γ-CD (H_1, 4.89 ppm) was observed in any sample.

<u>Swelling behavior of these topological gel networks</u>

To ensure consistent and reproducible quantification of gel swelling behavior, a standardized vacuum-assisted sieve filtration method was employed to remove excess surface solvent [11]. The mass of a clean 316 stainless steel wire sieve (250 × 250 mesh, 0.0024" opening) was recorded as W_1. Gel samples were dried under vacuum at ~25 °C overnight, and their dry mass was measured as W_0. The dried gels were then immersed in 60 mL of solvent at room temperature for 30 h to allow them to reach swelling equilibrium. Fully swollen gels were subsequently transferred onto the pre-weighed wire sieve, and vacuum was applied to remove residual surface solvent. The total mass of the sieve and swollen gel was recorded as W_2. The swelling ratio (wt%) was calculated using the following Equation:

$$\text{Swelling Ratio (wt\%)} = \frac{W_2 - W_1 - W_0}{W_0} \times 100$$

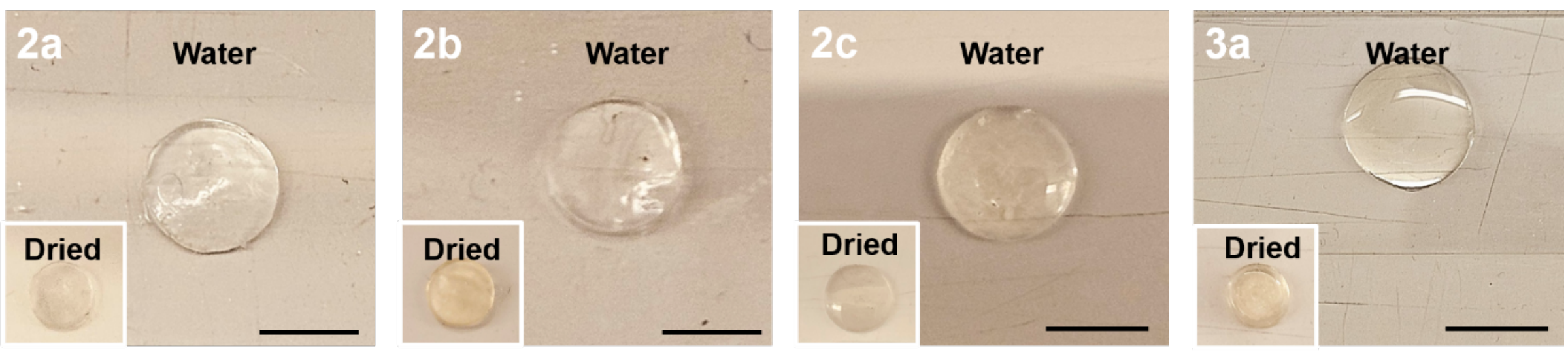

Figure S18. Optical images of dried and water-swollen gel networks.

Crosslinking density analysis

To provide a quantitative basis for the crosslinking density differences discussed in the main text, the effective number of monomers between crosslinks (N_x) was estimated using a simplified structural model. **Figure S19a** depicts the network architecture of **2a**, in which N_x denotes the number of monomer units between adjacent crosslinking junctions. The network synthesis was carried out using 4000 equivalents of 2-hydroxyethyl acrylate (HEA) monomers per **1^9Acr$_2$:γ-CD$_2$** crosslinker unit (**Figure S19b**). Given that the molecular length of the **1^9Acr** segment is significantly shorter than that of the long HEA-based linear chain segments, the doubly threaded ring crosslinks can be reasonably approximated as point-like crosslinking junctions for the purpose of network analysis. However, the effective functionality of the doubly threaded junction is not necessarily fixed at f = 8. As illustrated in **Figure S19c**, when all acrylates generate independent strand connections, the junction can be described as f = 8. In contrast, because the acrylate groups are located in close spatial proximity within the same doubly threaded crosslinker, two nearby acrylates may become incorporated into the same network strand, reducing the number of topologically independent chain connections and giving lower effective functionality values such as f = 6 or f = 4. To account for this structural ambiguity, an intermediate effective functionality value of $f_{eff} = 6$ was adopted for estimating N_x in **2a** (**Figure S19d**). Assuming that the HEA-to-crosslinker feed ratio used in the pregel formulation is reflected in the simplified repeating-segment model, the stoichiometric ratio of "HEA in the feed" : "crosslinker in the feed" can be equated to the corresponding ratio of "HEA in the repeating segment" : "crosslinker in the repeating segment". Based on the stoichiometric relationship between the monomer and crosslinker contents (4000 equiv. HEA per **1^9Acr$_2$:γ-CD$_2$** unit in **2a**), the average number of monomer repeat units between crosslinks can be approximated as: $4000 : 1 = 6 \times (N_x/2) : 1$, which yields an estimated value of N_x ca. 1333 monomer repeat units between adjacent crosslinks. This value represents the longest effective network strand length among the gel systems studied.

In contrast, complete dissociation of the doubly threaded pseudo[4]rotaxane crosslink in **2c** produces two independent singly threaded **1^9Acr:γ-CD** junctions. Unlike the doubly threaded junction in **2a**, whose effective functionality depends on how spatially proximal acrylates are incorporated into the network, each **1^9Acr:γ-CD** in **2c** can be more straightforwardly treated as an f = 4 junction because each acrylate is incorporated into a separate polymer strand. Using **1^9Acr:γ-CD** as the crosslinker unit, the feed ratio corresponds to HEA:crosslinker = 4000 : 2. Assuming this feed ratio is reflected in the simplified repeating

segment model, the average number of monomer repeat units between crosslinks can be approximated as: $4000 : 2 = 4 \times (N_x/2) : 1$, giving an estimated N_x of ca. 1000 repeat units (**Figure S20**). Because **3a** was designed as a covalent analogue of **2c** without γ-CD, it is likewise expected to possess a comparable crosslinking density and a similar N_x value. Since N_x is inversely related to effective crosslink density, these connectivity-based estimates indicate that **2a** has a lower effective crosslink density than **2c** and **3a**, with **2b** expected to be intermediate. These values should be viewed as relative model-based estimates rather than absolute chemical crosslink densities, because network defects, incomplete strand incorporation, and solvent-controlled junction mobility can also affect swelling and mechanical properties.

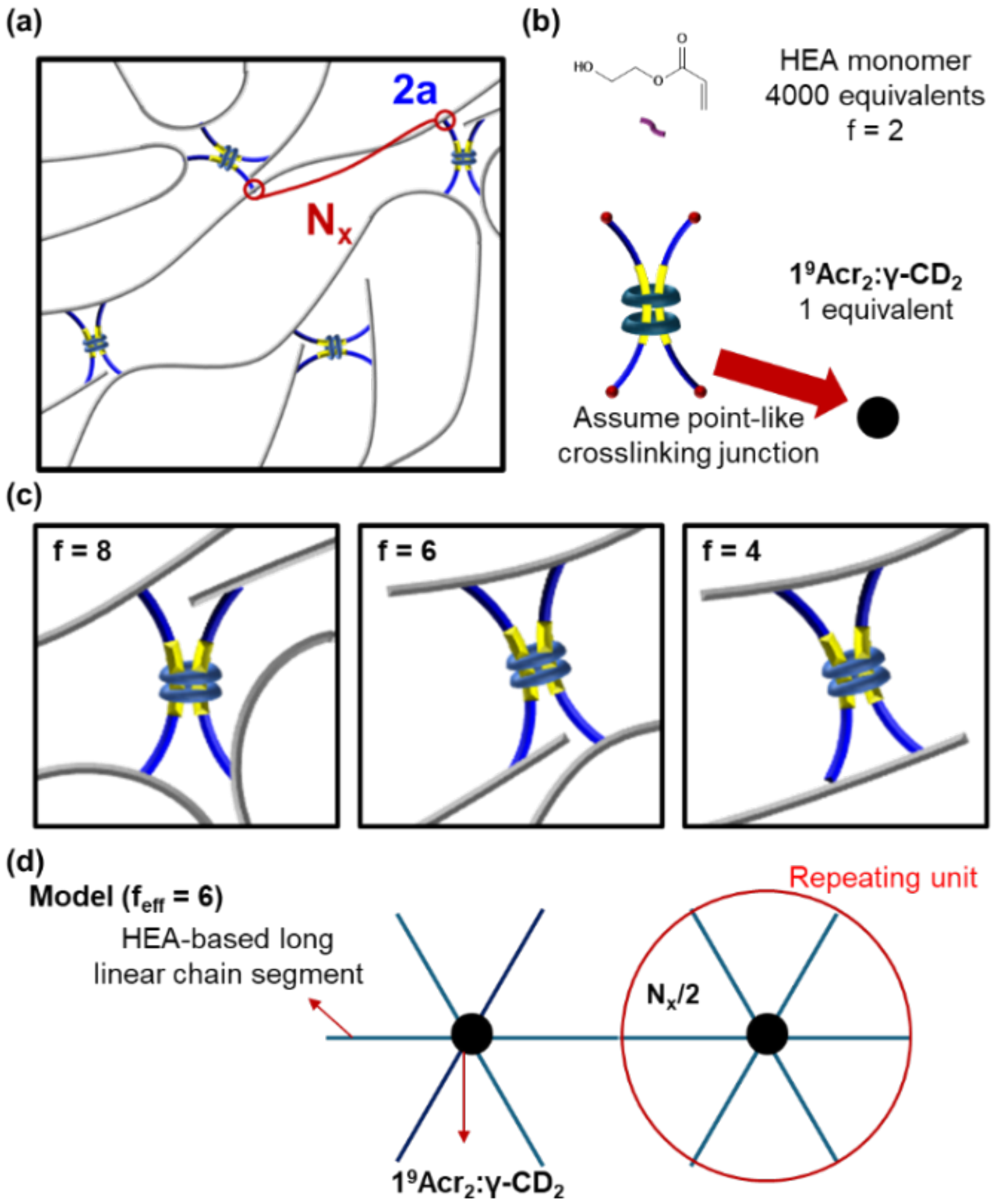

Figure S19. Connectivity models used to estimate N_x for **2a** (a) Schematic representation of **2a**, where N_x denotes the average number of monomer repeat units between adjacent crosslinking junctions. (b) HEA monomers (4000 equiv.) and $\mathbf{1^9Acr_2}$:γ-CD_2 crosslinker (1 equiv.) used for **2a**; the crosslinker was approximated as a point-like junction for the purpose of structural analysis. (c) Possible effective junction functionalities for the doubly threaded crosslinker (f = 8, 6, and 4), depending on how spatially proximal acrylates are incorporated into the network. (d) Simplified model used to estimate N_x in **2a** using an intermediate effective functionality value of $f_{eff} = 6$.

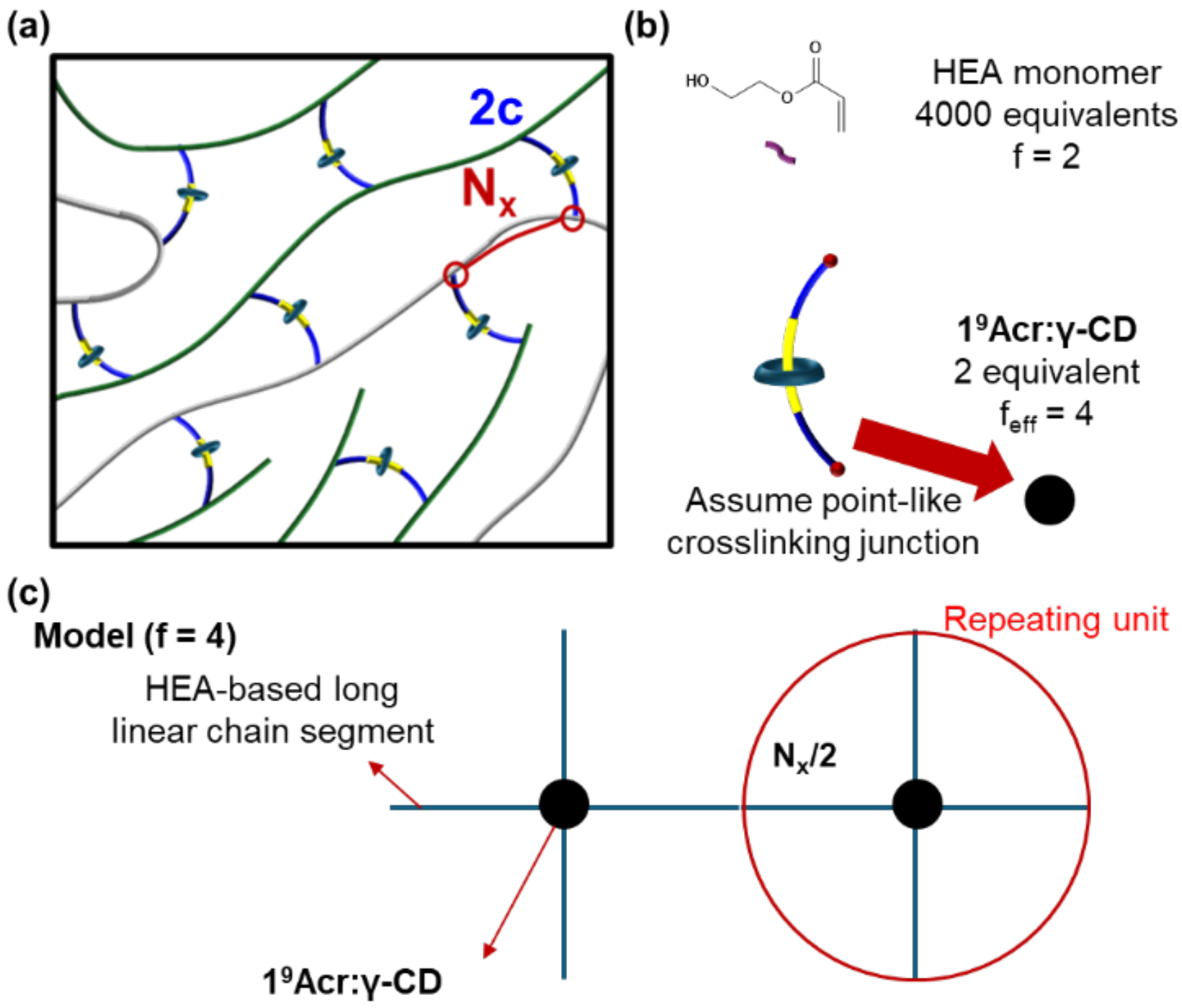

Figure S20. Connectivity models used to estimate N_x for **2c** (a) Schematic representation of **2c**, where N_x denotes the average number of monomer repeat units between adjacent crosslinking junctions. (b) HEA monomers (4000 equiv.) and **1[9]Acr**:γ-CD crosslinker (2 equiv.) used for **2c**; the crosslinker was approximated as a point-like junction for the purpose of structural analysis. (c) Simplified model used to estimate N_x in **2c** using a functionality value of f = 4.

Viscoelastic properties of gel networks

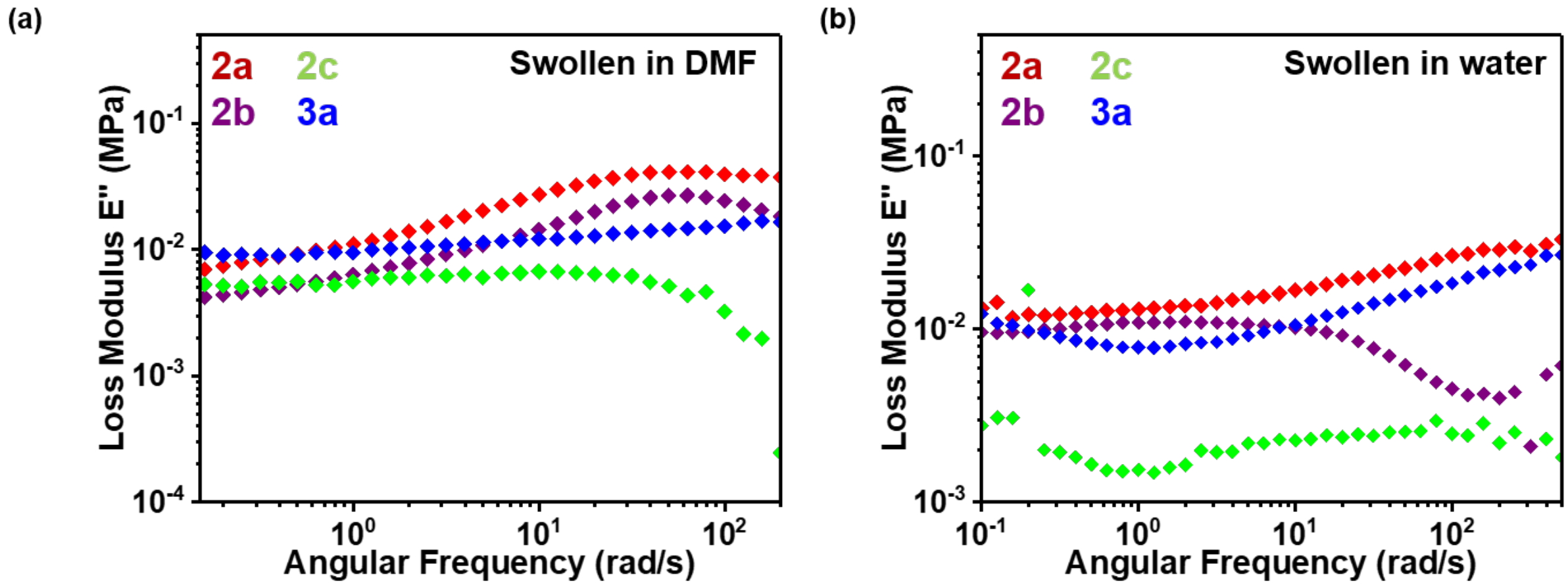

Figure S21. Loss moduli E” of the gel networks swollen in (a) DMF and (b) water.

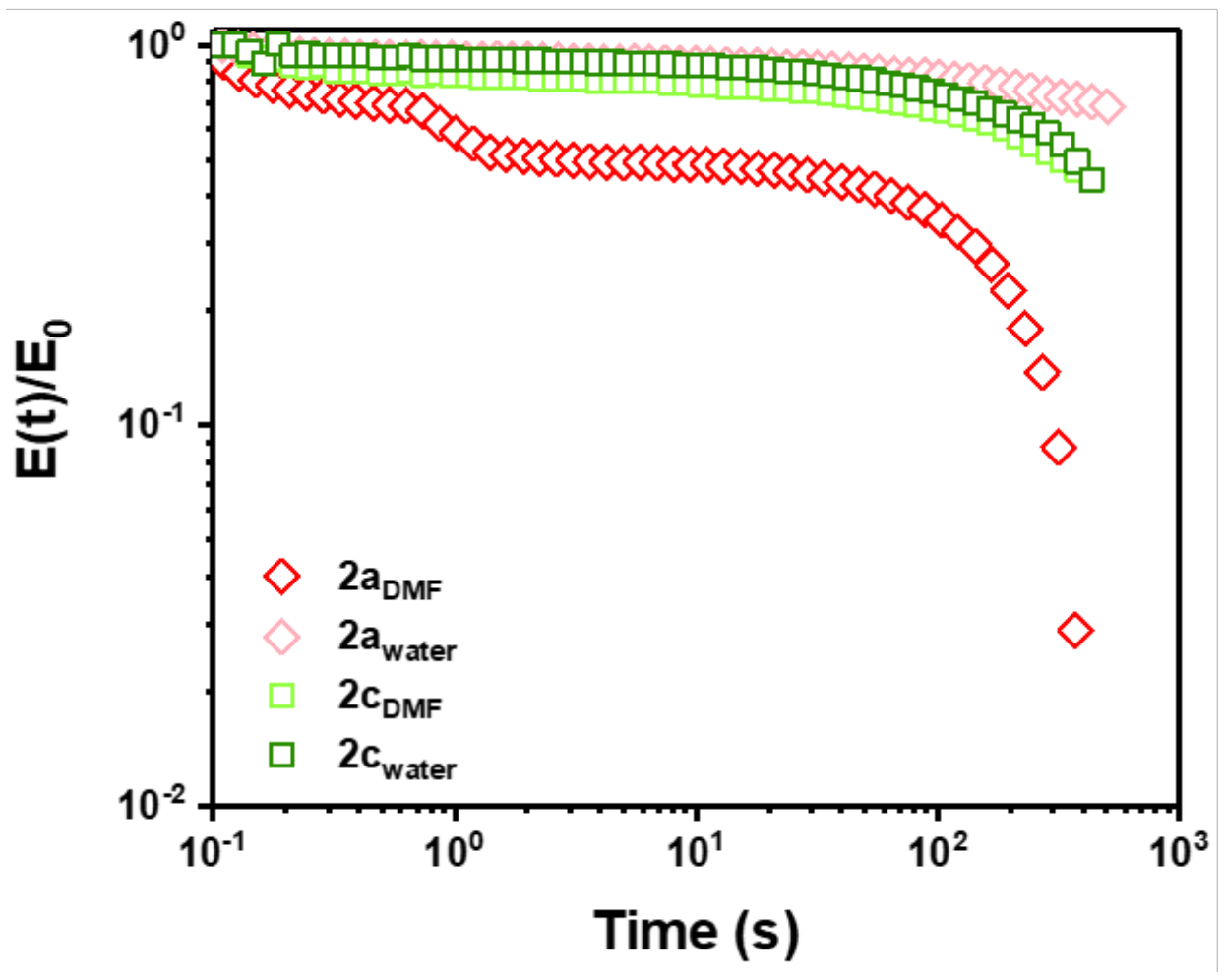

Figure S22. Compressive stress relaxation behavior of representative gel networks (**2a** and **2c**) fully swollen in DMF or water. E(t) denotes the relaxation modulus at time t, and E_0 denotes the initial relaxation modulus at t = 0; the data are plotted as the normalized relaxation modulus, $E(t)/E_0$.

Characterization of corresponding dried networks

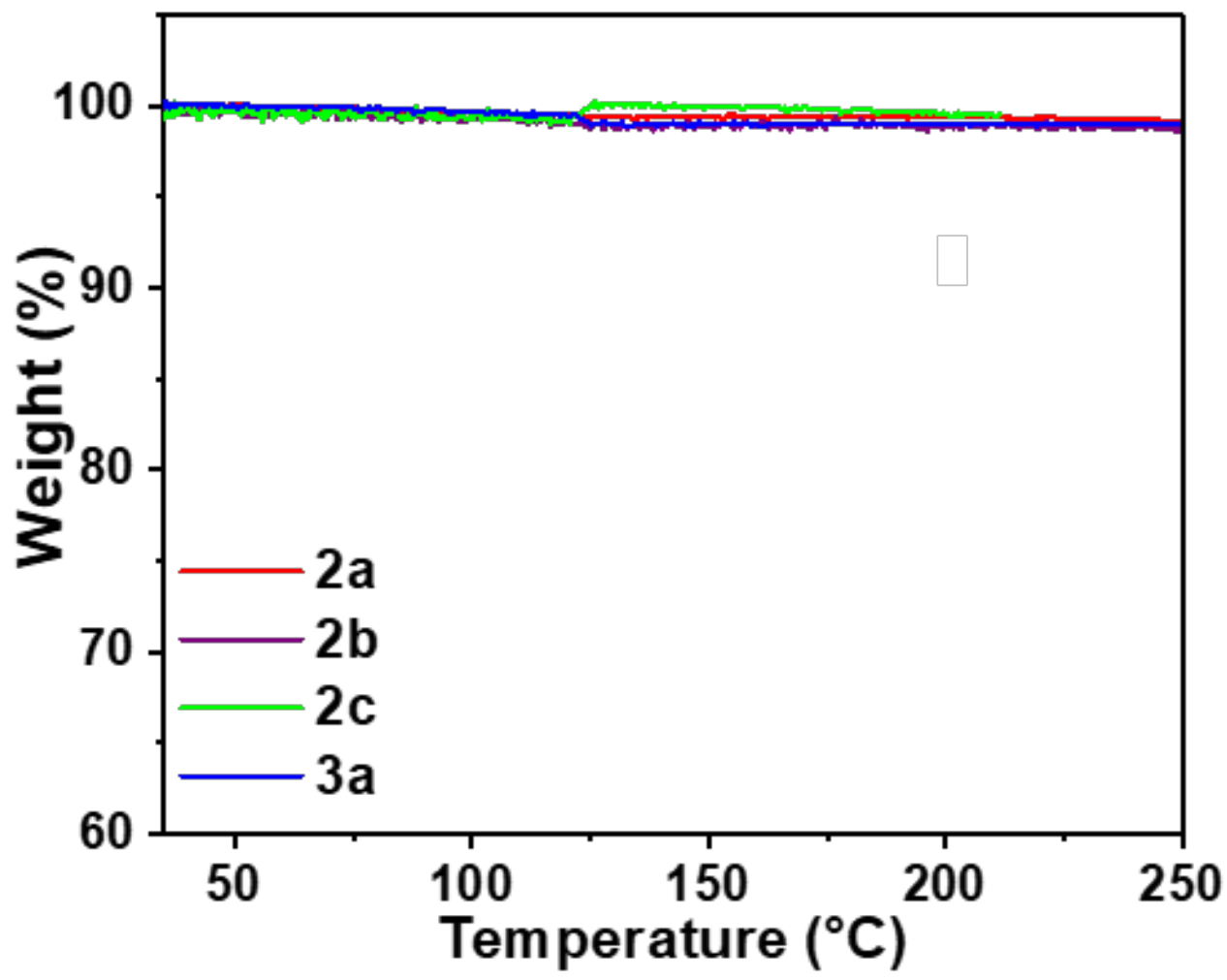

Figure S23. Thermogravimetric analysis (TGA) confirming complete residual solvent removal from dried gel networks.

	Rupture strain (%)		Young's Modulus (MPa)		Toughness (MJ/m^3)	
	Mean	SD	Mean	SD	Mean	SD
**2a**	1079.64	23.60	0.673	0.044	3.392	0.211
**2b**	770.66	81.57	1.148	0.149	2.933	0.326
**2c**	499.42	28.67	1.724	0.079	2.348	0.152
**3a**	427.61	60.99	1.315	0.086	1.668	0.162

Table S2. Tensile properties of dried gel networks (average of three specimens).

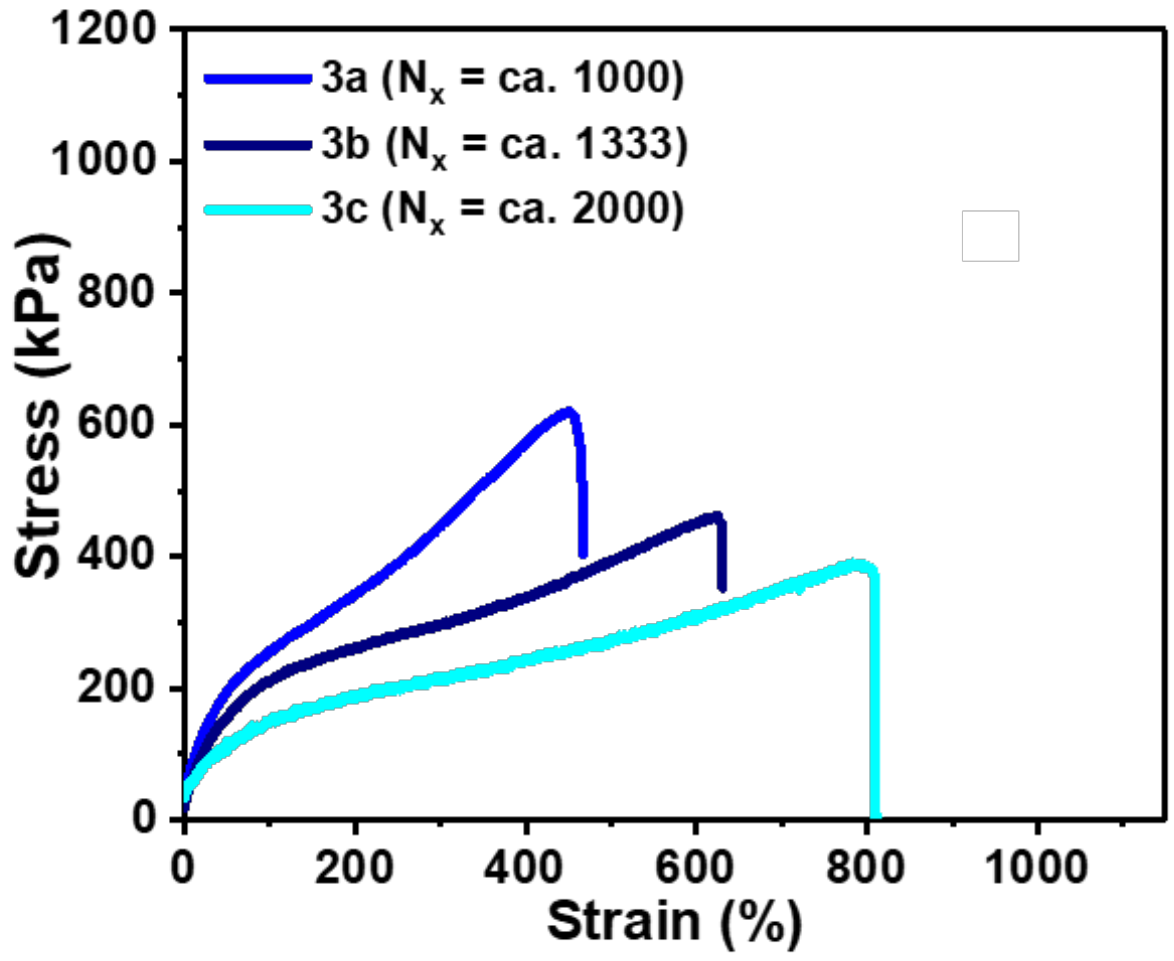

Figure S24. Tensile stress–strain curves of the additional covalent control networks (**3a**, **3b**, and **3c**) prepared at HEA-to-**1**[9]**Acr** molar feed ratios of 2000:1, 2667:1, and 4000:1, corresponding to estimated N_x values of ca. 1000, 1333, and 2000, respectively.

	Rupture strain (%)	Young's Modulus (MPa)	Toughness (MJ/m^3)
**3a**	427.61	1.315	1.668
**3b**	631.11	0.986	1.909
**3c**	809.72	0.761	1.987

Table S3. Tensile properties of the additional covalent control networks (**3a**, **3b**, and **3c**) with different estimated numbers of repeat units between crosslinks (N_x).

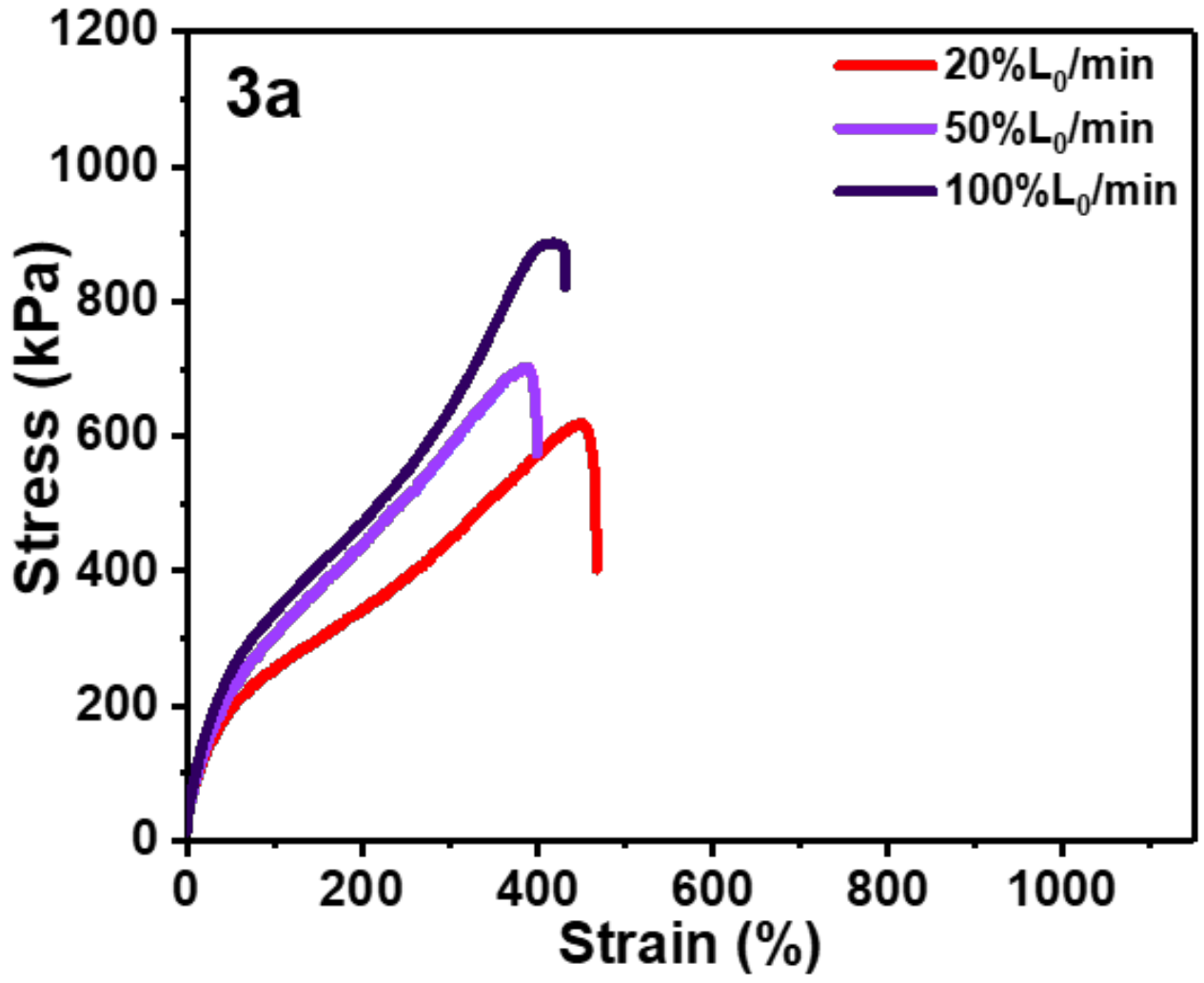

Figure S25. Strain rate-dependent tensile behavior of **3a** tested at 20%, 50%, and 100% L_0/min.

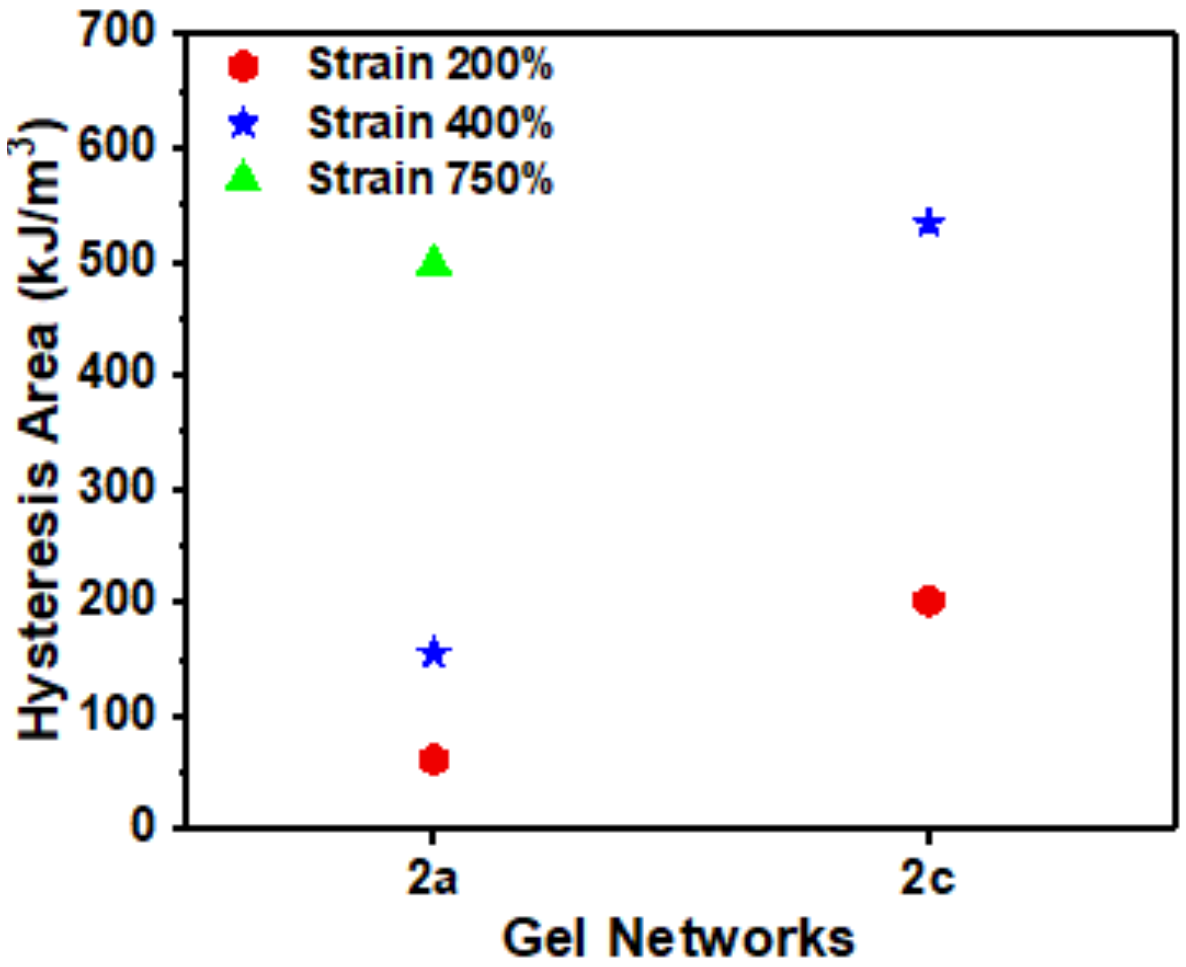

Figure S26. Hysteresis area of **2a** and **2c** at maximum strains of 200%, 400%, 750%, calculated from the loading–unloading curves in **Figures 8c** and **8d**. Cyclic tensile experiments were performed at a strain rate of 20% L_0/min.

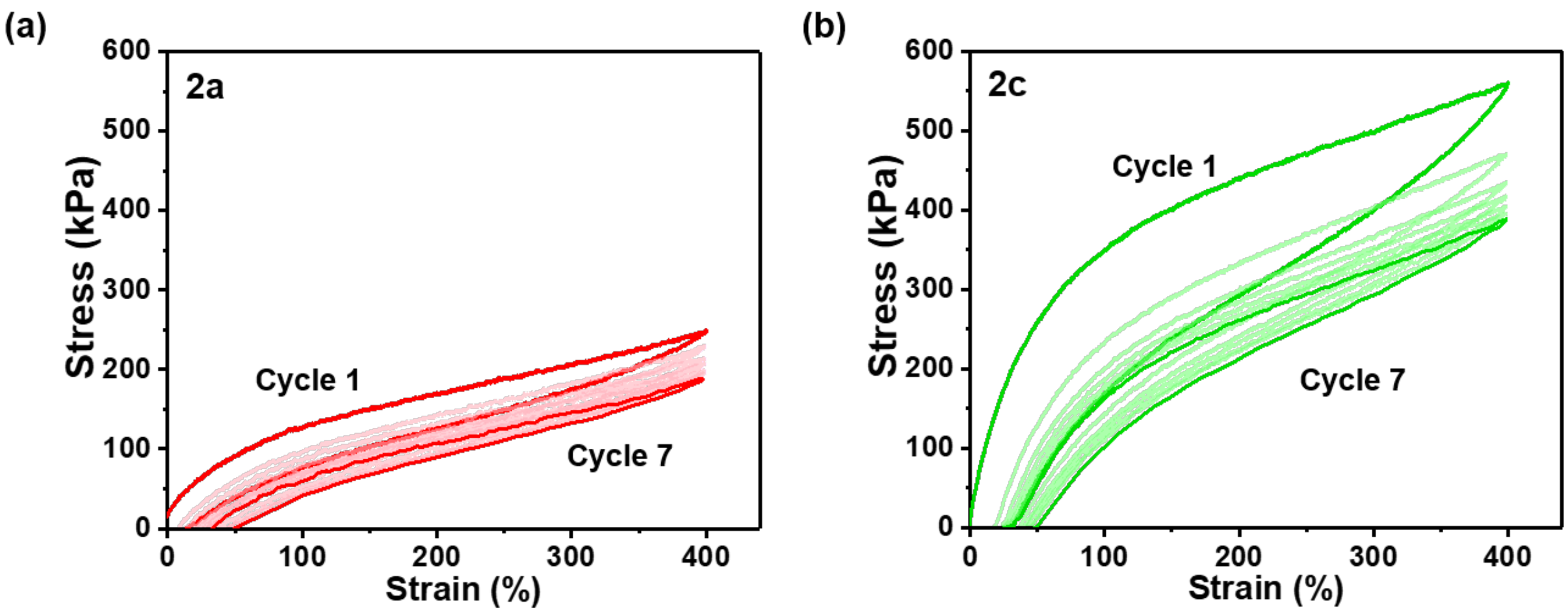

Figure S27. Fixed-strain cyclic tensile tests of dried (a) **2a** and (b) **2c** at a maximum strain of 400% for 7 consecutive cycles.

Sample	Cycle	Loading energy (kJ/m^3)	Unloading energy (kJ/m^3)	Hysteresis area (kJ/m^3)	Hysteresis ratio (%)	Residual strain (%)	Recovery efficiency (%)
**2a**	1	649.0	494.0	155.0	24.0	14.0	96.0
	7	392.0	335.0	57.0	15.0	50.0	88.0
**2c**	1	1636.0	1105.0	530.0	32.0	32.0	92.0
	7	922.0	774.0	149.0	16.0	50.0	88.0

Table S4. Quantitative analysis of fixed-strain cyclic tensile tests of dried 2a and 2c at 400% strain for 7 consecutive cycles. Hysteresis ratio was calculated as $(W_{loading} - W_{unloading})/W_{loading} \times 100\%$, and recovery efficiency was calculated as $(\varepsilon_{max} - \varepsilon_{residual})/\varepsilon max \times 100\%$, where ε_{max} is the maximum applied strain.

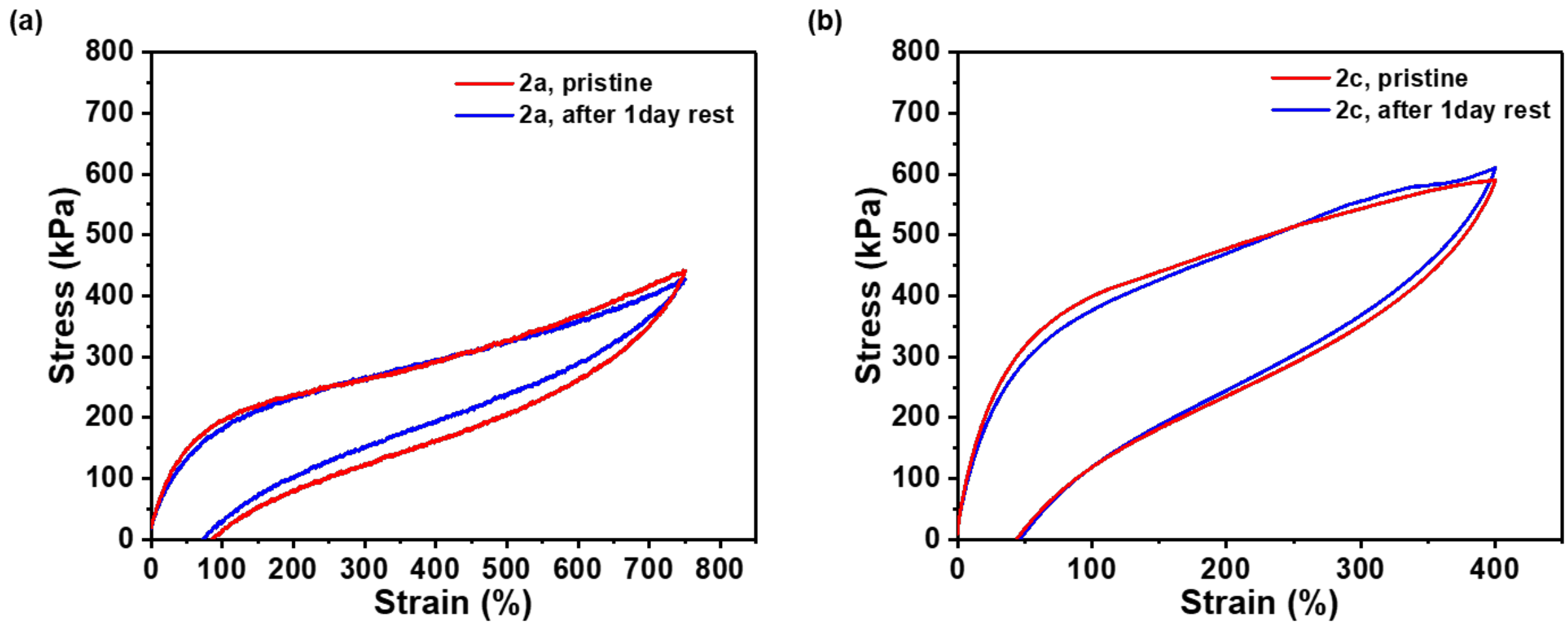

Figure S28. Time-dependent stability of cyclic tensile response. Cyclic stress–strain loops of (a) dried **2a** (750% strain amplitude) and (b) dried **2c** (400% strain amplitude) measured on the same specimens immediately after testing ("pristine") and upon repeating the measurement after 1 day rest under vacuum.

Captions for movies

Movie S1. Tensile deformation of **2a** at 100% L_0/min. Video is played at 50× speed.

Movie S2. Tensile deformation of **2a** at 20% L_0/min. Video is played at 100× speed.

Movie S3. Tensile deformation of **2c** at 100% L_0/min. Video is played at 50× speed.

Movie S4. Tensile deformation of **2c** at 20% L_0/min.. Video is played at 100× speed.

Reference

[1] A. Jerschow, N. Müller, *J. Magn. Reson.* **1996**, *123*, 222-225
[2] A. Jerschow, N. Müller, *J. Magn. Reson.* **1997**, *125*, 372-375.
[3] *Bruker Topspin 1D and 2D Experiments Step-by-step Tutorial; Advanced Experiments User Guide* **2006**.
[4] G. R. PÃŠrez-Lemus, C. A. Menendez, Y. Xu, P. F. Z. Rico, Y. Jin, J. J. de Pablo, *arXiv preprint arXiv:2504.13575* **2025**.
[5] C. Cézard, X. Trivelli, F. Aubry, F. Djedaïni-Pilard, F.-Y. Dupradeau, *Physical Chemistry Chemical Physics* **2011**, *13*, 15103-15121.
[6] X. He, V. H. Man, W. Yang, T.-S. Lee, J. Wang, *The Journal of chemical physics* **2020**, *153*.
[7] P. Eastman, M. S. Friedrichs, J. D. Chodera, R. J. Radmer, C. M. Bruns, J. P. Ku, K. A. Beauchamp, T. J. Lane, L.-P. Wang, D. Shukla, *Journal of chemical theory and computation* **2013**, *9*, 461-469.
[8] K. A. Feenstra, C. Peter, R. M. Scheek, W. F. van Gunsteren, A. E. Mark, *Journal of biomolecular NMR* **2002**, *23*, 181-194.

[9] D. Xu, T. A. P. Seery, Y. Gao, L. Ding, C. Zhou, Z. Wang, Z. Jiang, H. Zhang, *Journal of Applied Polymer Science* **2020**, *137*, 48379.

[10] H. Teisala, P. Baumli, S. A. L. Weber, D. Vollmer, H.-J. Butt, *Langmuir* **2020**, *36*, 4416-4431.

[11] K. Zhang, W. Feng, C. Jin, *MethodsX* **2020**, *7*, 100779.
